# Supplementary material for: Pif1-Family Helicases Support Fork Convergence during DNA Replication Termination in Eukaryotes
Source: Mol Cell. 2019 Apr 18;74(2):231–244.e9. doi: 10.1016/j.molcel.2019.01.040 (PMC6477153; doi:10.1016/j.molcel.2019.01.040)
Supplement: Document S1. Figures S1–S7 and Tables S1–S5 [file mmc1.pdf]

Molecular Cell, Volume *74*

Supplemental Information

Pif1-Family Helicases Support Fork Convergence during DNA Replication Termination in Eukaryotes

Tom D. Deegan, Jonathan Baxter, María Ángeles Ortiz Bazán, Joseph T.P. Yeeles, and Karim P.M. Labib

Figure S1, related to Figure 1

Convergence of reconstituted replisomes is defective over a wide range of experimental conditions. (**A**) Plasmids used in this study, with restriction sites that were used for linearisation. (**B**) Reaction scheme for standard *in vitro* replication reactions. (**C**) *S. cerevisiae* DNA replication proteins were purified as described in STAR Methods, and then visualised by SDS-PAGE and coomassie staining. (**D**) Fork convergence is defective during replication of different plasmid templates (see STAR methods and (A) for details). Reaction products were linearised before native agarose gel analysis with the indicated enzymes. (**E**) Replication of the 3.2 kb plasmid template in the indicated concentrations of potassium glutamate. The reaction products were purified, linearised with SbfI and then analysed in a native agarose gel. (**F**) The 3.2 kb plasmid was replicated for 20' in the presence ^{32}P-dCTP, before addition of a chase of 600 μM unlabelled dNTPs. Samples were taken at the indicated times, before analysis by digestion and native agarose gel electrophoresis, indicating that LRIs persist stably and thus represent an endpoint of the replication reaction under these conditions. (**G**) The 3.2 kb template was replicated for the indicated times in the presence of a 'minimal' replisome (Yeeles et al., 2015). The reactions lacked Ctf4, Csm3-Tof1, Mrc1, Top2, Pol δ, RFC, PCNA, ligase and Fen1. As before, the purified samples were analysed by SmaI digestion and native agarose gel electrophoresis.

Figure S2, related to Figure 2

Replication of plasmids with a single active origin of DNA replication. (**A**) Micrococcal nuclease digestion of the chromatinized 3.2 kb plasmid template

used in Figure 2A. The products were analysed in a 1.5% native agarose gel with ethidium bromide staining. (**B**) Replication of the circular (1) and linearised (2-3) versions of the 5.5 kb plasmid template shown in Fig. 2B. The reactions were performed in the absence of Polδ, ligase and Fen1, and were analysed by denaturing agarose gel electrophoresis. The figure indicates the positions of the ~2.75 kb leading strand products for the circular vector and the ~5.5 kb leading strand products for the linearised plasmids.

Figure S3, related to Figure 4

The ability of accessory DNA helicases to support fork convergence in the reconstituted DNA replication system. (**A**) Control experiment, in which the 3.2 kb plasmid was incubated with Sgs1, Top3-Rmi1 and RPA, showing that purified Sgs1 and Top3-Rmi1 are active for relaxation and catenation of dsDNA. The positions of the various plasmid isoforms are indicated. Sgs1 and Top3-Rmi1 were omitted as indicated. (**B**) Analogous reactions to those in Figure 4C were performed in the presence of the indicated concentrations of Sgs1-Top3-Rmi1 (Sgs1-T-R), before analysis by native agarose gel electrophoresis. (**C**) Samples from A were analysed by denaturing agarose gel electrophoresis, illustrating that higher concentrations of Sgs1-Top3-Rmi1 inhibited DNA synthesis in the reconstituted replication system. (**D**-**H**) The 3.2 kb plasmid template was replicated in the presence of varying concentrations of the indicated proteins. The reaction products were purified, digested, and analysed in native agarose gels.

Figure S4, related to Figure 4

Pif1 supports fork converge on chromatinised and linearised plasmid templates. (**A**) A chromatinised versions of the 3.2 kb plasmid was prepared as for Figure 2A, and replicated in the presence of FACT, with or without Pif1 as indicated. The products were digested with SpeI and analysed in a native agarose gel. (**B**) Pulse-chase replication reaction conducted as depicted in Figure 3A with 9.7 kb plasmid template. Pif1 was added as indicated, concomitantly with SmaI, to test whether Pif1 could promote fork convergence on a linearised DNA template. Those samples that were not linearised during the experiment (1-2) were subsequently digested with SmaI before native gel analysis.

Figure S5, related to Figure 5

Pif1-Rrm3 support fork convergence but CMG is still essential for replication. (**A**) The 9.7 kb plasmid template pZN3 was replicated in the presence of ^{32}P-dCTP for 2’ 50”, before addition of a chase of cold dNTPs, together with Pif1+Rrm3 as indicated. Pol δ, ligase and Fen1 were omitted from these reactions, so that un-ligated leading strands could be monitored. Samples were removed and quenched at the indicated times, before analysis in a denaturing agarose gel. (**B**) Replication reactions containing Pif1 but lacking the indicated factors that are required for CMG assembly and activation. Products were analysed by digestion and native agarose gel electrophoresis. (**C**) Similar reactions containing Rrm3 but lacking the indicated factors.

Figure S6, related to Figure 6

Type II topoisomerase activity makes a modest contribution to fork convergence in the reconstituted replication system. (**A**) Replication of the 3.2 kb plasmid template in the presence of the indicated topoisomerases from *S. cerevisiae* (*S.c.*) or *E. coli* (*E.c.*). Products were analysed by SbfI digestion and native agarose gel electrophoresis. (**B**) Similar experiments to (A) were performed three times. The percentage full-length products was quantified in each case, and the figure presents the mean values with standard deviations (**C**) Analogous experiments to the one in Figure 6A were performed three times, and then quantified as above.

Figure S7, related to Figure 7

Pif1 and Rrm3 have a partially redundant role in promoting fork convergence during plasmid replication *in vivo*. (**A**) Control cells (W303-1a), *rrm3Δ* (yBH131), *pif1Δ* (yMO294) and *pif1-m2 rrm3Δ* (yMO291) were grown and analysed as in Figure 7A-B. The positions of the various plasmid isoforms are indicated, and the red arrow denotes late replication intermediates (LRIs), which accumulated to a greater extent in *pif1-m2 rrm3Δ* cells compared to the *rrm3Δ* single mutant. (**B**) The plasmid pRS425, illustrating the SnaBI restriction site that was used for linearization in Figure 7C, and the Late Replication Intermediate (LRI) that results from transient stalling of forks during DNA replication termination *in vivo*.

Figure S1, related to Figure 1

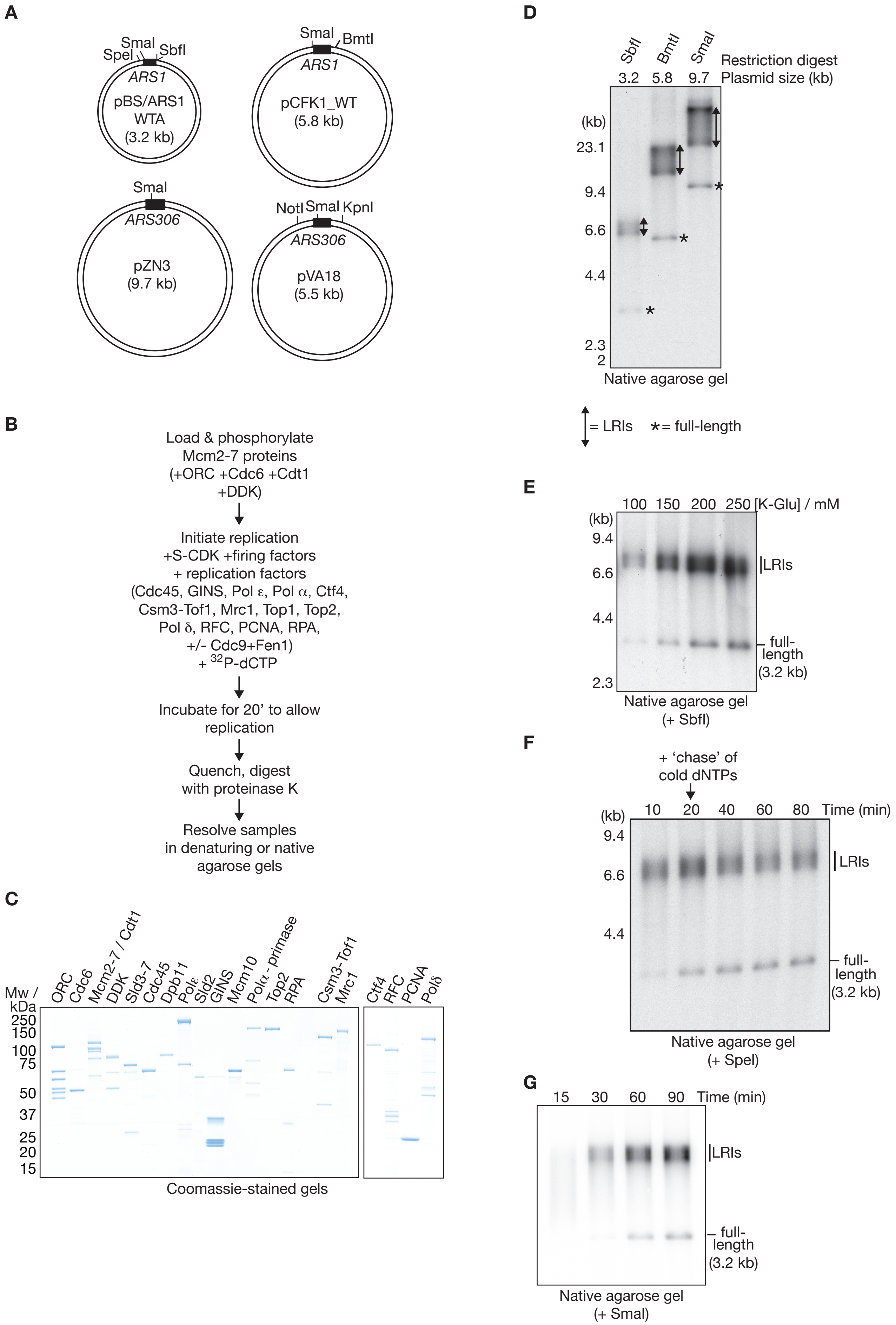

Figure S2, related to Figure 2

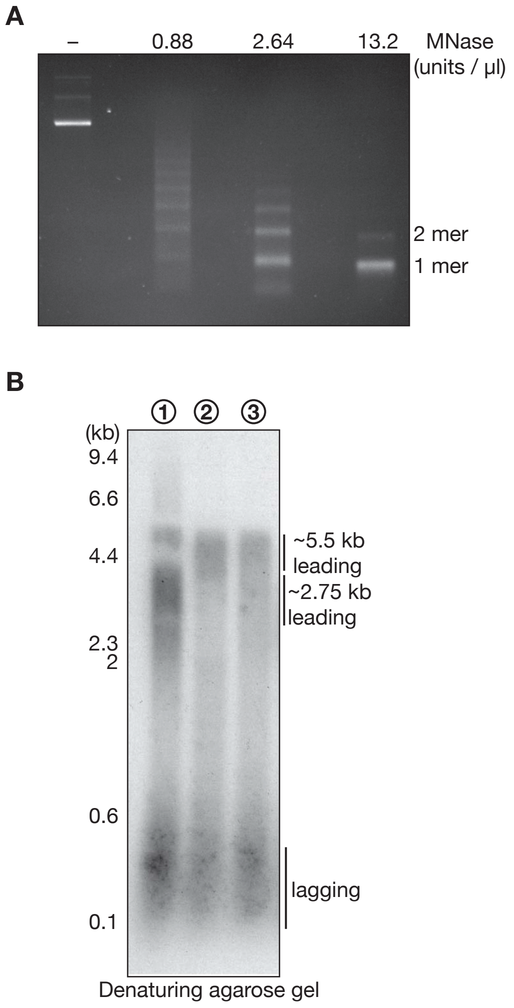

① Circular template

② NotI-linearised template (origin left end)

③ KpnI-linearised template (origin right end)

Figure S3, related to Figure 4

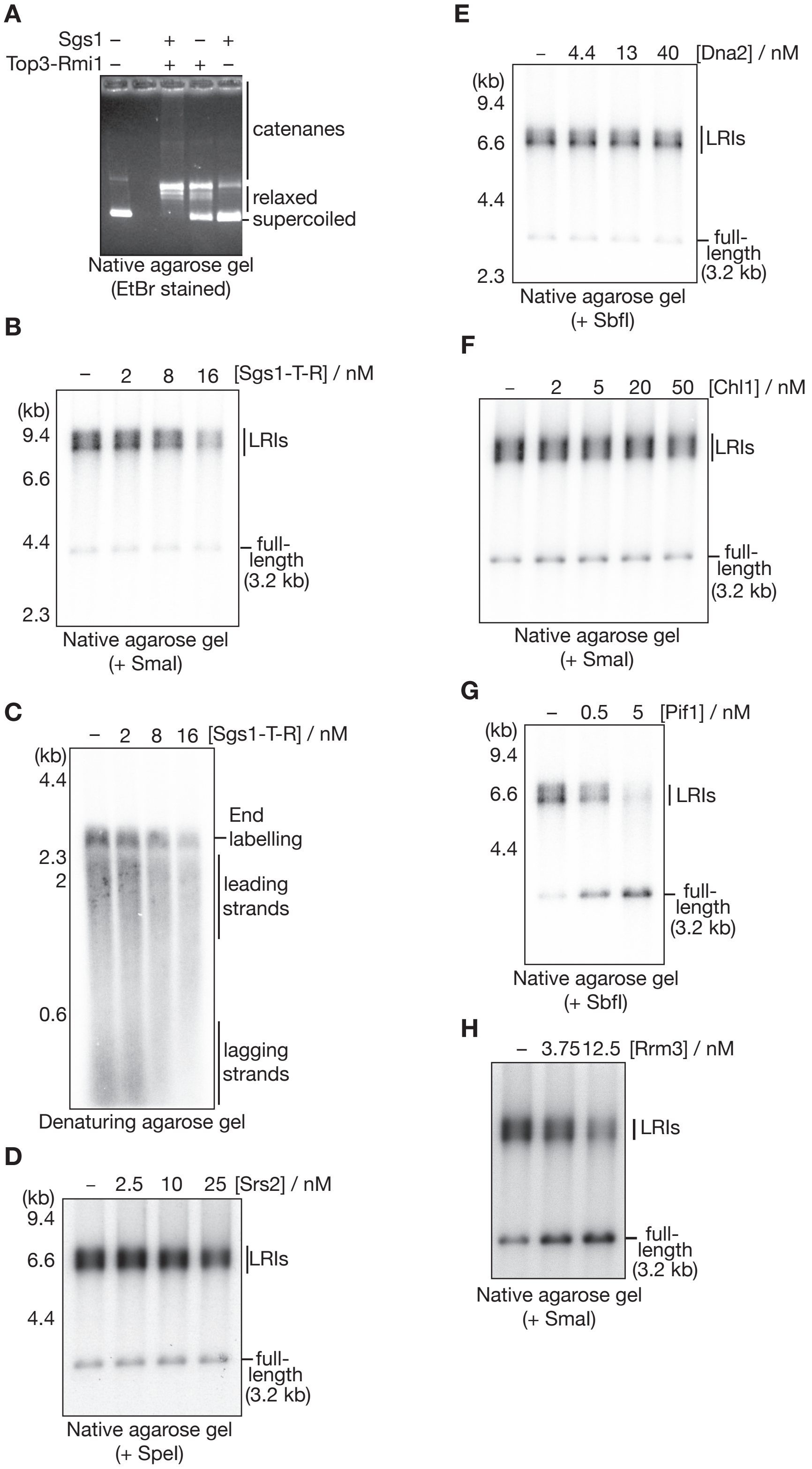

Figure S4, related to Figure 4

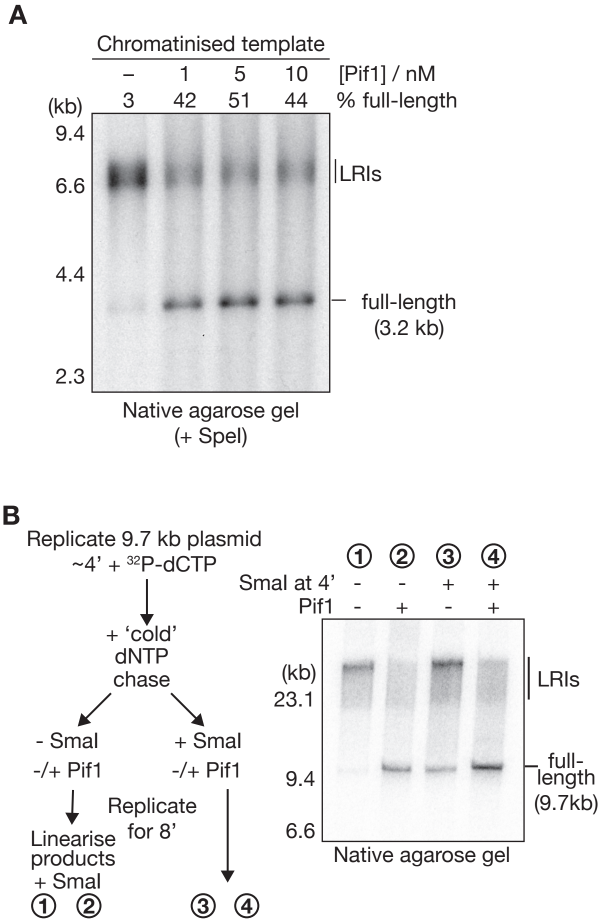

Figure S5, related to Figure 5

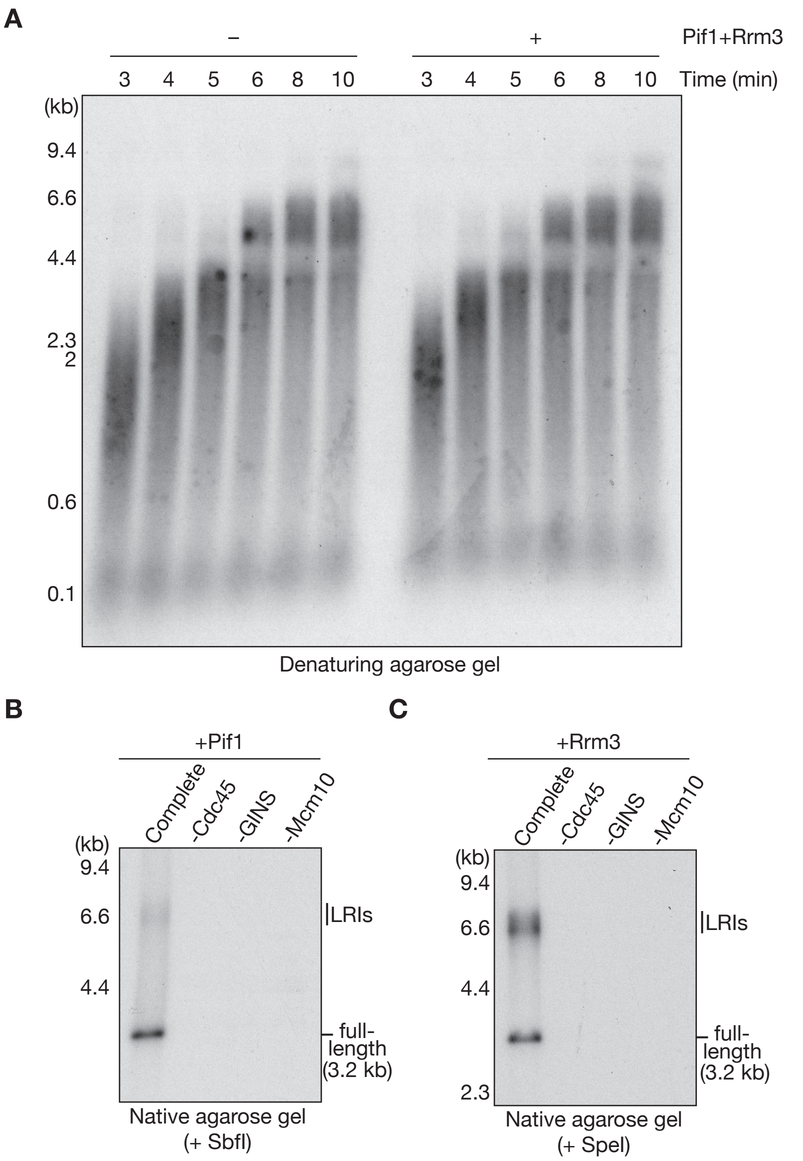

Figure S6, related to Figure 6

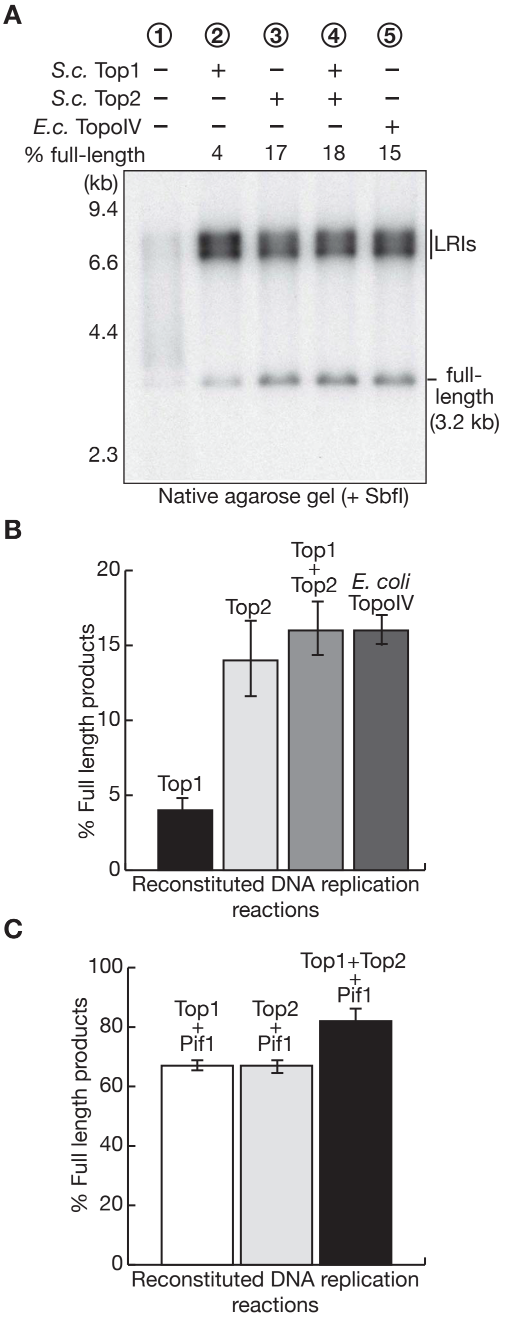

Figure S7, related to Figure 7

A

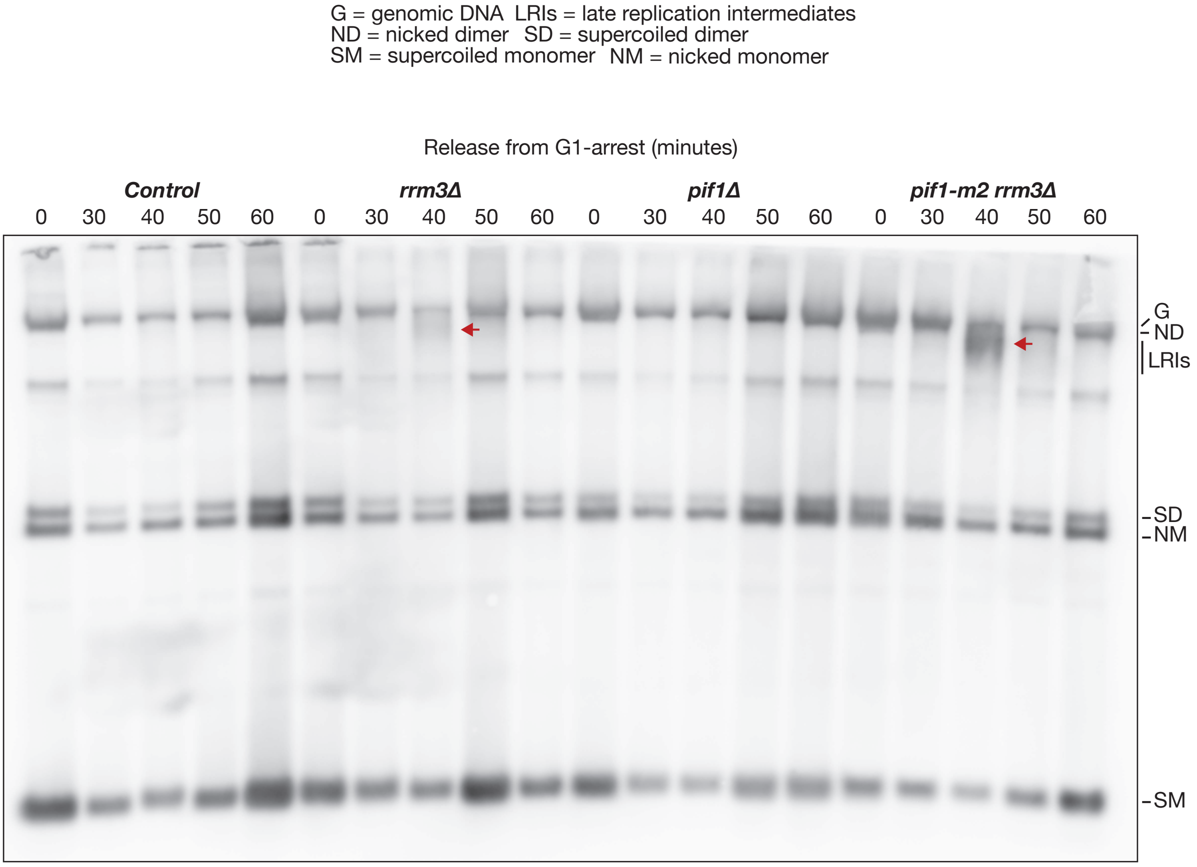

Native agarose gel (plasmid pRS425)

B

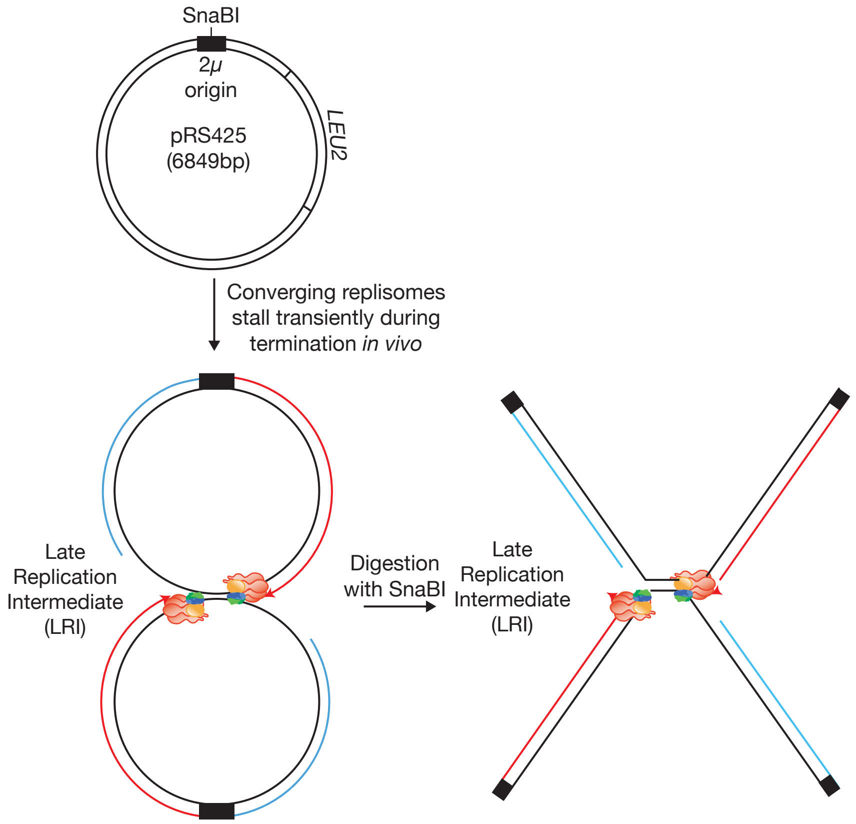

Plasmid	Original vector	Insert	Plasmid construction
pTDK4	pRS305	*Tof1-GAL1,10-CBP-TEV-Csm3*	Synthetic SacI-XmaI construct
pTDK8	pRS305	*Gal1,10-CBP-TEV-Top1*	Synthetic SacI-XmaI construct
pTDK13	pTDK8	*CBP-TEV-Sgs1*	Synthetic SpeI-XmaI construct
pTDK15	pTDK8	*3Flag-TEV-Rrm3*	Synthetic SpeI-XmaI construct
pTDK18	pTDK8	*Fen1-2Flag*	Synthetic SpeI-XmaI construct
pTDK19	pTDK8	*Cdc9-2Flag*	Synthetic SpeI-XmaI construct
pTDK34	pTDK15	*3Flag-TEV-Rrm3 K260A*	Site directed mutagenesis of pTDK15 using primers 7813 and 7814
pTDK10	pET28c	*Pif1 nuclear isoform (Pif1Δ2-40)*	PCR amplification of Pif1Δ2-40 from W303 genomic DNA using primers 6912 and 6913 cloned 5' NdeI, 3' SalI
pTDK24	pTDK10	*Pif1 nuclear isoform (Pif1Δ2-40) K264A*	Site directed mutagenesis of pTDK10 using primers 7103 and 7104
pTDK31	pET28c	*Pif1 from Bacteroides sp 2 1 16 (BacPif1)*	Synthetic NdeI-NotI construct
pFV36	pKL120	*PrA-CBP-TEV-Dna2*	Synthetic SpeI-XmaI construct

Table S1, Related to STAR Methods. Protein expression plasmids generated in this study. Codon usage of genes in pTDK4, pTDK8, pTDK13, pTDK15, pTDK18, pTDK19, pTDK34 and pFV36 was optimised for protein expression in *S. cerevisiae* as described previously (Yeeles et al., 2015).

Oligonucleotide name	Usage	Sequence
6912	Pif1 forward primer for construction of pTDK10	ACGCACATATGAGTAGTCGTGGTTTCAGG
6913	Pif1 reverse primer for construction of pTDK10	TAGTCGTCGACTTATTCTAAGATGTGGTCTTCG
7080	Oligonucleotide used for annealing to M13 ssDNA for generation of helicase assay substrate with 40bp duplex	GATCGACTACGTCAGCTCTAGAGGATCCCCGGGTACCGAGCTCGAATTCGTAATC
7103	Forward primer for mutagenesis of Pif1 to generate pTDK24	GGGAGTGCCGGTACCGGTGCATCCATTCTTTTACGTG
7104	Reverse primer for mutagenesis of Pif1 to generate pTDK24	CACGTAAAAGAATGGATGCACCGGTACCGGCACTCCC
7272	Forward primer for generation of molecular weight markers	ATGGTTACTAAGCCATCCC
7273	Reverse primer for generation of 2.9 kb molecular weight marker	GCTTGTCAATACCCATACC
7274	Reverse primer for generation of 3 kb molecular weight marker	CAGTAGGAGTAGTTACCG
7275	Reverse primer for generation of 3.1 kb molecular weight marker	CTTGTTCAAGTGCTTTTCC
7813	Forward primer for mutagenesis of Rrm3 to generate pTDK34	GGTTCCGCTGGTACTGGTGCTTCTGTTATTTTGCAAACC
7814	Reverse primer for mutagenesis of Rrm3 to generate pTDK34	GGTTTGCAAAATAACAGAAGCACCAGTACCAGCGGAACC
8371	Oligonucleotide used for annealing to M13 ssDNA for generation of helicase assay substrate with 25bp duplex	ACTACGTCAGTACCGAGCTCGAATTCGTAATCATG

Table S2, Related to STAR Methods. Oligonucleotides used in this study. All oligonucleotides were generated for this study.

Protein	Tag	Purification steps
ORC	CBP-TEV (Orc1)	1. Calmodulin affinity purification<br>2. HiTrap Q chromatography
Cdc6	GST	1. GST affinity purification<br>2. Elution by cleavage with 3C protease<br>3. Hydroxyapatite chromatography
Cdt1-Mcm2-7	CBP-TEV (Mcm3)	1. Calmodulin affinity purification<br>2. Gel filtration (Superdex 200)
DDK	CBP-TEV (Dbf4)	1. Calmodulin affinity purification<br>2. Dephosphorylation (Lambda protein phosphatase)<br>3. Gel filtration (Superdex 200)
S-CDK	CBP-TEV (Clb5)	1. Calmodulin affinity purification<br>2. Elution by cleavage with TEV protease<br>3. Gel filtration (Superdex 200)
Sld3-7	TEV-CBP-PrA (Sld3)	1. IgG affinity purification<br>2. Elution by cleavage with TEV protease<br>3. Gel filtration (Superdex 200)
Cdc45	Internal 2FLAG	1. Anti-FLAG affinity purification<br>2. HiTrap Q chromatography
Dpb11	3FLAG	1. Anti-Flag affinity purification<br>2. MonoS chromatography
Pol ε	TEV-CBP (Dpb4)	1. Calmodulin affinity purification<br>2. HiTrap heparin chromatography<br>3. Gel filtration (Superdex 200)
Sld2	3FLAG	1. Ammonium sulphate precipitation<br>2. Anti-Flag affinity purification<br>3. HiTrap SP chromatography
GINS	6HIS (Psf3)	1. Ni-NTA affinity purification<br>2. HiTrap Q chromatography<br>3. Gel filtration (Superdex 200)
Mcm10	6HIS	1. Ni-NTA affinity purification

		2. MonoS chromatography (two rounds)<br>3. Gel filtration (Superdex 200)
Ctf4	CBP-TEV	1. Calmodulin affinity purification<br>2. Gel filtration (Superdex 200)
Top2	TEV-CBP	1. Calmodulin affinity purification<br>2. Gel filtration (Superdex 200)
PCNA	Untagged	1. Polymin P precipitation of nucleic acids<br>2. Ammonium sulphate precipitation of proteins<br>3. HiTrap SP and HiTrap heparin chromatography (in tandem)<br>4. HiTrap DEAE chromatography<br>5. HiTrap Q chromatography<br>6. Gel filtration (Superdex 200)
RPA	CBP-TEV (Rfa1)	1. Calmodulin affinity purification<br>2. HiTrap heparin chromatography<br>3. HiTrap Q chromatography
ISW1	3FLAG (Ioc3)	1. Anti-Flag affinity purification<br>2. MonoQ chromatography
Nap1	GST	1. GST affinity purification<br>2. Elution by cleavage with 3C protease<br>3. MonoQ chromatography
Nhp6	Untagged	1. Trichloroacetic acid precipitation<br>2. HiTrap SP chromatography
FACT	HIS	1. TALON affinity purification<br>2. MonoQ chromatography
Histones	Untagged	1. HiTrap heparin chromatography<br>2. Gel filtration (Superdex 200)

Table S3, Related to STAR Methods. Protein purification strategies used in this study.

Protein	Source
ORC	(Frigola et al., 2013)
Cdc6	(Frigola et al., 2013)
Cdt1-Mcm2-7	(Coster et al., 2014)
DDK	(On et al., 2014)
Sld3/7	(Yeeles et al., 2015)
Cdc45	(Yeeles et al., 2015)
Dpb11	(Yeeles et al., 2015)
Sld2	(Yeeles et al., 2015)
Pol ε	(Yeeles et al., 2015)
GINS	(Yeeles et al., 2015)
S-CDK	(Yeeles et al., 2015)
Mcm10	(Yeeles et al., 2015)
Pol α	(Yeeles et al., 2015)
RPA	(Yeeles et al., 2015)
Ctf4	(Yeeles et al., 2015)
Mrc1	(Yeeles et al., 2017)
Csm3-Tof1	This study
RFC	(Yeeles et al., 2017)
PCNA	(Yeeles et al., 2017)
Pol δ	(Yeeles et al., 2017)
Top1	This study
Top2	(Yeeles et al., 2015)
Fen1	This study
Cdc9	This study
ISWI	(Kurat et al., 2017)
Nap1	(Kurat et al., 2017)
Nhp6	(Kurat et al., 2017)
FACT	(Kurat et al., 2017)
Histones	(Kurat et al., 2017)
Sgs1	Kowalczykowski lab
Top3-Rmi1	Kowalczykowski lab
Pif1	This study
BacPif1	This study
Rrm3	This study
Chl1	This study
Dna2	This study
Srs2	This study
TopoIV	Inspiralis (T4001)

Table S4, Related to STAR Methods. Recombinant proteins used in this study.

Strain	Source
*W303-1a: MAT***a** *ade2-1 ura3-1 his3-11,15 trp1-1 leu2-3,112 can1-100*	Labib Laboratory
*yJF1: MAT***a** *ade2-1 ura3-1 his3-11,15 trp1-1 leu2-3,112 can1-100 bar1Δ::hphNT pep4Δ::kanMX*	(Frigola et al., 2013)
*ySDORC (ORC purification): MAT***a** *ade2-1 ura3-1 his3-11,15 trp1-1 leu2-3,112 can1-100 bar1::hphNT pep4::kanMx his3::pRS303-ORC3+ORC4 ura3::pRS306-CBP-TEV-ORC1+ORC2 trp1::pRS304-ORC5+ORC6*	(Frigola et al., 2013)
*yAM33 (Cdt1-Mcm2-7 purification): MAT***a** *ade2-1 ura3-1 his3-11,15 trp1-1 leu2-3,112 can1-100 bar1::hphNT pep4::kanMx his3::pRS303-CDT1+GAL4 ura3::pRS306-MCM2+CBP-TEV-MCM3 trp1::pRS304-MCM4+MCM5 leu2::pRS305-MCM6+MCM7*	(Coster et al., 2014)
*ySDK8 (DDK purification): MAT***a** *ade2-1 ura3-1 his3-11,15 trp1-1 leu2-3,112 can1-100 bar1::hphNT pep4::kanMX trp1::pRS304-CDC7+CBP-TEV-DBF4*	(On et al., 2014)
*yTD6 (Sld3-7 purification): MAT***a** *ade2-1 ura3-1 his3-11,15 trp1-1 leu2-3,112 can1-100 bar1::hphNT pep4::kanMX leu2::pRS305-SLD7 his3::pRS303-SLD3-TCP+GAL4*	(Yeeles et al., 2015)
*yTD8 (Sld2 purification): MAT***a** *ade2-1 ura3-1 his3-11,15 trp1-1 leu2-3,112 can1-100 bar1::hphNT pep4::kanMX his3::pRS303-SLD2-3FLAG(nat-NT2)+GAL4*	(Yeeles et al., 2015)
*yJY13 (Cdc45 purification): MAT***a** *ade2-1 ura3-1 his3-11,15 trp1-1 leu2-3,112 can1-100 bar1::hphNT pep4::kanMX his3::pRS303-CDC45-iFLAG2+GAL4*	(Yeeles et al., 2015)
*yJY26 (Dpb11 purification): MAT***a** *ade2-1 ura3-1 his3-11,15 trp1-1 leu2-3,112 can1-100 bar1::hphNT pep4::kanMX his3::pRS303-DPB11-3FLAG(nat-NT2)+GAL4*	(Yeeles et al., 2015)
*yAJ2 (Pol ε purification): MAT***a** *ade2-1 ura3-1 his3-11,15 trp1-1 leu2-3,112 can1-100 bar1::hphNT pep4::kanMX trp1::pRS304-POL2+DPB4-TEV-CBP ura3::pRS306DPB2+DPB3*	(Yeeles et al., 2015)
*yAE37(S-CDK purification): MAT***a** *ade2-1 ura3-1 his3-11,15 trp1-1 leu2-3,112 can1-100 bar1::hphNT pep4::kanMX ura3::pRS306-CKS1+CDC28 his3::pRS303-CBP-TEV-CLB5+GAL4*	(Yeeles et al., 2015)
*yAE40 (Ctf4 purification): MAT***a** *ade2-1 ura3-1 his3-11,15 trp1-1 leu2-3,112 can1-100 bar1::hphNT pep4::kanMX his3::pRS303-CBP-TEV-CTF4+GAL4*	(Yeeles et al., 2015)
*yJY23 (Pol α / primase purification): MAT***a** *ade2-1 ura3-1 his3-11,15 trp1-1 leu2-3,112 can1-100 bar1::hphNT pep4::kanMX trp1::pRS304-POL1+POL12 ura3:: pRS306-CBP-TEV-PRI1+PRI2*	(Yeeles et al., 2015)

*yAE34 (Pol δ purification): MAT***a** *ade2-1 ura3-1 his3-11,15 trp1-1 leu2-3,112 can1-100 bar1::hphNT pep4::kanMX ura3::pRS306-POL31+POL3 his3::pRS303-POL32-CBP+GAL4*	(Yeeles et al., 2017)
*yAE41 (RFC purification): MAT***a** *ade2-1 ura3-1 his3-11,15 trp1-1 leu2-3,112 can1-100 bar1::hphNT pep4::kanMX ura3::pRS306-RFC2+CBP-RFC3 trp1::pRS304-RFC4+RFC5 his3::pRS303-RFC1+GAL4*	(Yeeles et al., 2017)
*yAE71 (Mrc1 purification): MAT***a** *ade2-1 ura3-1 his3-11,15 trp1-1 leu2-3,112 can1-100 bar1::hphNT pep4::kanMX his3::pRS303-MRC1-5FLAG*	Diffley laboratory
*yAE31 (RPA purification): MAT***a** *ade2-1 ura3-1 his3-11,15 trp1-1 leu2-3,112 can1-100 bar1::hphNT pep4::kanMX his3::pRS303-CBP-TEV-RFA1+GAL4 ura3::pRS306-RFA2+RFA3*	(Yeeles et al., 2017)
*yAE46 (Top2 purification): MAT***a** *ade2-1 ura3-1 his3-11,15 trp1-1 leu2-3,112 can1-100 bar1::hphNT pep4::kanMX trp1::pRS304-TOP2-TEV-CBP+GAL4*	(Yeeles et al., 2015)
*yCFK1 (Isw1a purification): MAT***a** *ade2-1 ura3-1 his3-11,15 trp1-1 leu2-3,112 can1-100 bar1Δ::hphNT pep4Δ::kanMX IOC3-3FLAG(nat-NT2)*	(Kurat et al., 2017)
*yTDK4 (Csm3-Tof1 purification): MAT***a** *ade2-1 ura3-1 his3-11,15 trp1-1 leu2-3,112 can1-100 bar1Δ::hphNT pep4Δ::kanMX leu2::pRS305-TOF1+CBP-TEV-CSM3*	This study
*yTDK9 (Rrm3 purification): MAT***a** *ade2-1 ura3-1 his3-11,15 trp1-1 leu2-3,112 can1-100 bar1Δ::hphNT pep4Δ::kanMX leu2::pRS305-3FLAG-TEV-RRM3*	This study
*yTDK17 (Rrm3-K260A purification): MAT***a** *ade2-1 ura3-1 his3-11,15 trp1-1 leu2-3,112 can1-100 bar1Δ::hphNT pep4Δ::kanMX leu2::pRS305-3FLAG-TEV-rrm3-K260A*	This study
*yTDK6 (Top1 purification): MAT***a** *ade2-1 ura3-1 his3-11,15 trp1-1 leu2-3,112 can1-100 bar1Δ::hphNT pep4Δ::kanMX leu2::pRS305-CBP-TEV-TOP1*	This study
*yFV43 (Dna2 purification): MAT***a** *ade2-1 ura3-1 his3-11,15 trp1-1 leu2-3,112 can1-100 pep4Δ::ADE2 ura3::pRS306-ProteinA-CBP-TEV-DNA2*	This study
*yTDK18 (Fen1 purification): MAT***a** *ade2-1 ura3-1 his3-11,15 trp1-1 leu2-3,112 can1-100 bar1Δ::hphNT pep4Δ::kanMX leu2::pRS305-FEN1-2FLAG*	This study
*yTDK19 (Cdc9 purification): MAT***a** *ade2-1 ura3-1 his3-11,15 trp1-1 leu2-3,112 can1-100 bar1Δ::hphNT pep4Δ::kanMX leu2::pRS305-CDC9-2FLAG*	This study
*yBH131: MAT***a** *ade2-1 ura3-1 his3-11,15 trp1-1 leu2-3,112 can1-100 rrm3Δ::hphNT*	(Hodgson et al., 2007)
*yMO294: MAT***a** *ade2-1 ura3-1 his3-11,15 trp1-1 leu2-3,112 can1-100 pif1Δ::URA3CP*	This study
*yMO291: MAT***a** *ade2-1 ura3-1 his3-11,15 trp1-1 leu2-3,112 can1-100 pif1-m2 rrm3Δ::hphNT*	This study

Table S5, Related to STAR Methods. *S. cerevisiae* strains used in this study.
