# Supplementary material for: Pif1-Family Helicases Support Fork Convergence during DNA Replication Termination in Eukaryotes
Source: Mol Cell. 2019 Apr 18;74(2):231–244.e9. doi: 10.1016/j.molcel.2019.01.040 (PMC6477153; doi:10.1016/j.molcel.2019.01.040)
Supplement: Document S2. Article plus Supplemental Information [file mmc2.pdf]

Article

Molecular Cell

Pif1-Family Helicases Support Fork Convergence during DNA Replication Termination in Eukaryotes

Graphical Abstract

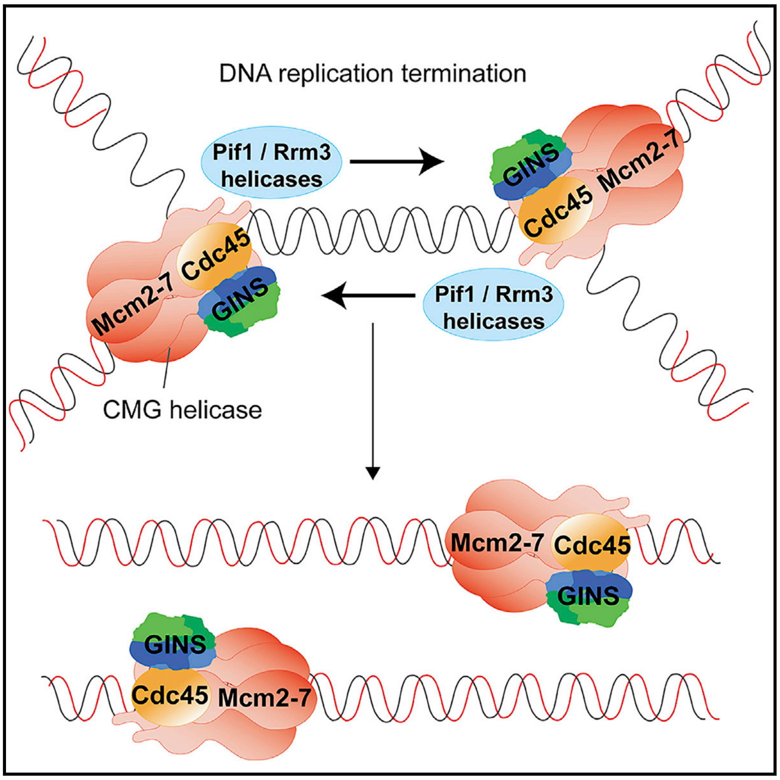

Highlights

- *In vitro* reconstitution of the convergence of two eukaryotic replisomes
- Converging replisomes stall at a late stage of DNA replication termination
- The budding yeast DNA helicases Pif1 and Rrm3 stimulate fork convergence *in vitro*
- Pif1 and Rrm3 promote fork convergence during DNA replication termination *in vivo*

Authors

Tom D. Deegan, Jonathan Baxter, María Ángeles Ortiz Bazán, Joseph T.P. Yeeles, Karim P.M. Labib

Correspondence

tdeegan@dundee.ac.uk (T.D.D.),
kpmlabib@dundee.ac.uk (K.P.M.L.)

In Brief

To study the mechanism of DNA replication termination, Deegan et al. reconstituted the convergence of two replisomes using purified budding yeast proteins. Surprisingly, replisome convergence is inherently inefficient but stimulated by the Pif1 and Rrm3 DNA helicases, which are also important during termination *in vivo*.

Deegan et al., 2019, Molecular Cell *74*, 231–244
April 18, 2019 © 2019 The Authors. Published by Elsevier Inc.
https://doi.org/10.1016/j.molcel.2019.01.040

CellPress

Pif1-Family Helicases Support Fork Convergence during DNA Replication Termination in Eukaryotes

Tom D. Deegan,[1,*] Jonathan Baxter,[2] María Ángeles Ortiz Bazán,[1] Joseph T.P. Yeeles,[3] and Karim P.M. Labib[1,4,*]
[1]The MRC Protein Phosphorylation and Ubiquitylation Unit, School of Life Sciences, University of Dundee, Dundee DD1 5EH, UK
[2]Genome Damage and Stability Centre, Department of Life Sciences, University of Sussex, Brighton BN1 9RQ, UK
[3]The MRC Laboratory of Molecular Biology, Cambridge Biomedical Campus, Francis Crick Avenue, Cambridge CB2 0QH, UK
[4]Lead Contact
*Correspondence: tdeegan@dundee.ac.uk (T.D.D.), kpmlabib@dundee.ac.uk (K.P.M.L.)
https://doi.org/10.1016/j.molcel.2019.01.040

SUMMARY

The convergence of two DNA replication forks creates unique problems during DNA replication termination. In *E. coli* and SV40, the release of torsional strain by type II topoisomerases is critical for converging replisomes to complete DNA synthesis, but the pathways that mediate fork convergence in eukaryotes are unknown. We studied the convergence of reconstituted yeast replication forks that include all core replisome components and both type I and type II topoisomerases. We found that most converging forks stall at a very late stage, indicating a role for additional factors. We showed that the Pif1 and Rrm3 DNA helicases promote efficient fork convergence and completion of DNA synthesis, even in the absence of type II topoisomerase. Furthermore, Rrm3 and Pif1 are also important for termination of plasmid DNA replication *in vivo*. These findings identify a eukaryotic pathway for DNA replication termination that is distinct from previously characterized prokaryotic mechanisms.

INTRODUCTION

In contrast to the initiation and elongation stages of eukaryotic DNA replication, which have been studied intensively over recent decades (Bell and Labib, 2016; Burgers and Kunkel, 2017; Deegan and Diffley, 2016), very little is known about the process of DNA replication termination, which occurs whenever two replisomes from neighboring replication origins meet each other (Dewar and Walter, 2017). The final stages of DNA synthesis involve at least five processes that are particular to termination (Dewar and Walter, 2017): the convergence and encounter of the two replisomes, gap filling between the leading strand at one fork and the lagging strand of the other (Dewar et al., 2015), regulated disassembly of the replisome (Dewar et al., 2017; Maric et al., 2014, 2017; Moreno et al., 2014; Sonneville et al., 2017), and decatenation of the sister chromatids that are the products of replication. The majority of these steps are understood very poorly, and almost nothing is known in any eukaryotic species about the mechanisms that drive fork convergence and replisome encounter.

During elongation, DNA unwinding by the replicative helicase causes torsional strain in the DNA template, which produces positive supercoils ahead of each replisome (Keszthelyi et al., 2016). The removal of these supercoils by type I or II topoisomerases is essential for continued fork progression in both prokaryotes and eukaryotes (Bermejo et al., 2007; Vos et al., 2011; Yeeles et al., 2015). When two forks converge, however, the remaining stretch of parental DNA becomes so short that supercoils cannot form (Vafabakhsh and Ha, 2012), and topoisomerases are sterically excluded (Keszthelyi et al., 2016). Therefore, the final stages of DNA unwinding and DNA synthesis are thought to require clockwise rotation of the two converging replication forks to transfer the topological stress behind the two replisomes in the form of intertwines or precatenanes between the replicated sister chromatids (Keszthelyi et al., 2016; Schalbetter et al., 2015).

During DNA replication termination in the bacterium *E. coli* and the virus SV40, the removal of precatenanes by type II topoisomerases is essential for the converging replisomes to continue unwinding and complete DNA synthesis (Hiasa and Marians, 1996; Ishimi et al., 1992; Richter et al., 1987; Snapka et al., 1988). Moreover, the resolution of converged forks during termination of SV40 replication in human cells appears to be a slow process that leads to the accumulation of a "late replication intermediate" (Seidman and Salzman, 1979; Sundin and Varshavsky, 1980). In contrast, however, type II topoisomerase activity is dispensable for the convergence of eukaryotic replisomes in budding yeast cells (Baxter and Diffley, 2008) and in *Xenopus* egg extracts (Dewar et al., 2015; Lucas et al., 2001). In addition, observations of DNA replication termination in *Xenopus* egg extracts indicated that two replisomes converge without detectable slowing or stalling (Dewar et al., 2015), in contrast to SV40 viral replication, despite the latter being dependent upon eukaryotic replication factors, apart from the viral DNA helicase T-antigen. Until now, the pathways supporting fork convergence in eukaryotes have remained enigmatic.

Here we analyze eukaryotic DNA replication termination *in vitro* using a reconstituted system based on purified budding yeast proteins that has been shown previously to support the initiation and elongation stages of chromosome duplication (Devbhandari et al., 2017; Yeeles et al., 2015, 2017). Our data identify a eukaryotic pathway for fork convergence that is

This is an open access article under the CC BY license (http://creativecommons.org/licenses/by/4.0/).

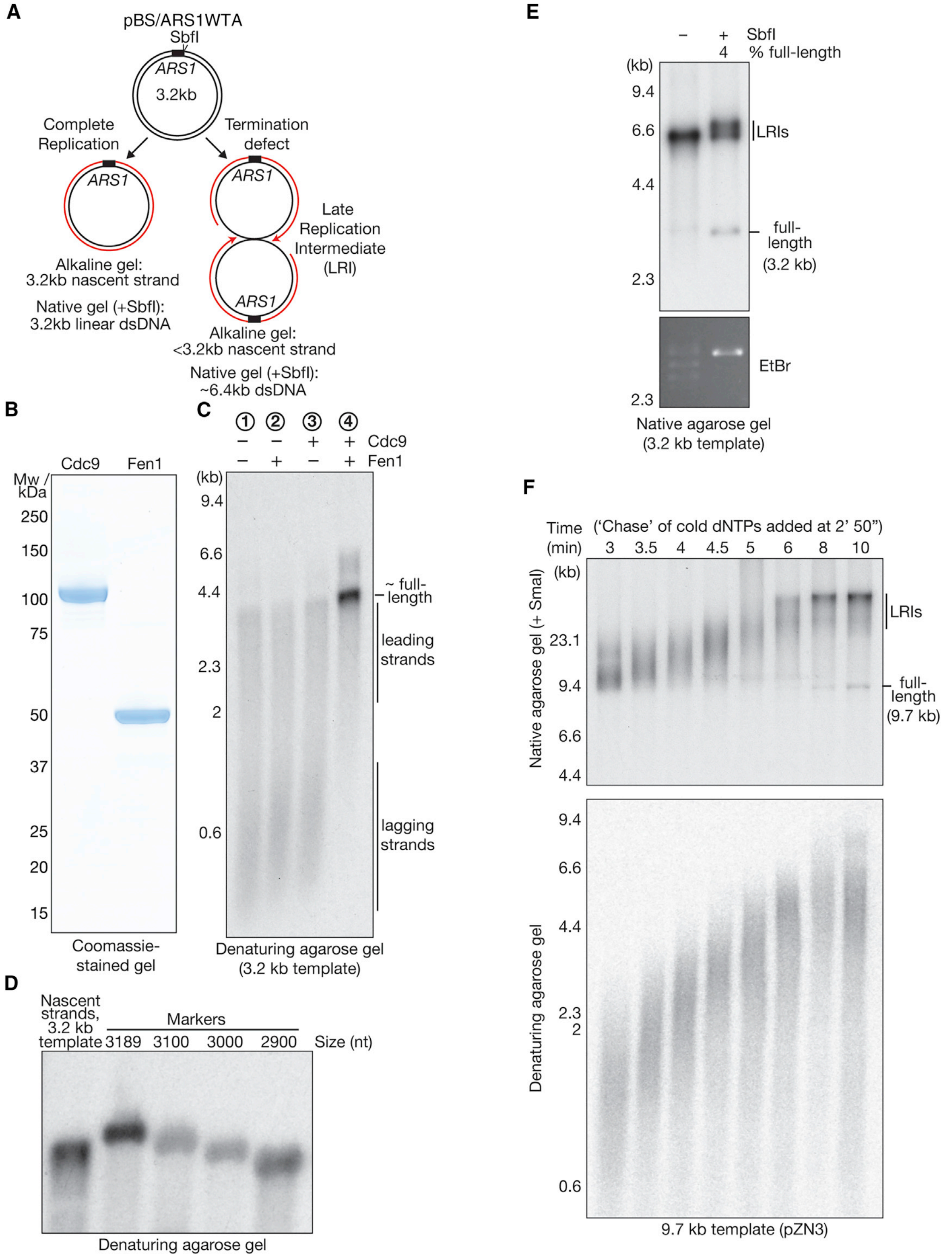

Figure 1. Converging Replisomes Stall in the Absence of Accessory DNA Helicases

(A) A 3,189-bp plasmid template (pBS/ARS1WTA) and the products of complete DNA replication (left) or a defect in DNA replication termination (right).

(B) Purified *S. cerevisiae* Cdc9 (ligase) and Fen1 were visualized by SDS-PAGE and Coomassie staining.

(C) A 3,189-bp plasmid template (pBS/ARS1WTA) was replicated according to the schematic in Figure S1B, and Fen1 and Cdc9 were included as indicated. Subsequently, the replication products were resolved in a denaturing agarose gel, and the radiolabeled nascent strands were detected by autoradiography.

(D) The products of replicating the 3,189-bp plasmid in the presence of Fen1 and ligase were analyzed in a denaturing agarose gel alongside the indicated markers.

(legend continued on next page)

mediated by Pif1-family DNA helicases and is independent of type II topoisomerase activity. Moreover, these findings lay the foundations for future studies of the mechanisms and regulation that govern DNA replication termination in eukaryotes.

RESULTS

Converging Replisomes Stall in the Absence of Accessory DNA Helicases

Previous work (Yeeles et al., 2015) established the minimal set of budding yeast proteins that is required *in vitro* to establish bidirectional forks from an origin of DNA replication on a circular plasmid template. In this system, the Mcm2-7 proteins (MCM [minichromosome maintenance]) that represent the catalytic core of the replicative helicase are first loaded as double hexamers onto double stranded DNA (dsDNA) at origins of replication and then activated in a separate step to form two CMG (Cdc45-MCM-GINS) helicases. A "minimal replisome" then assembles around CMG at each nascent DNA replication fork, with DNA polymerase α making primers for lagging-strand synthesis, whereas DNA polymerase ε extends the leading strand, and the type II topoisomerase Top2 removes supercoils to allow fork progression.

Further development of this reconstituted replication system (Yeeles et al., 2017) added components of the replisome progression complex that assembles around the yeast CMG helicase (Gambus et al., 2006), including the type I topoisomerase Top1, and also added DNA polymerase δ (Pol δ) and other factors that are required for lagging-strand synthesis. Under these conditions, the two replisomes move away from the origin at similar rates as those seen *in vivo* (Yeeles et al., 2017). Analogous to the situation at cellular replication forks, DNA polymerase α initiates every new DNA molecule, DNA polymerase ε (Pol ε) extends the leading strands, and Pol δ synthesizes each Okazaki fragment during lagging-strand synthesis (Yeeles et al., 2017).

As a first step toward addressing whether the reconstituted replisomes are able to support the completion of plasmid replication when two forks converge, we monitored nascent strand formation in reactions containing the flap endonuclease Fen1 and the DNA ligase Cdc9, which are required for Okazaki fragment processing and nascent strand ligation. Using a 3.2-kb plasmid template (Figures 1A and S1A; pBS/ARS1WTA), we observed the generation of approximately full-length nascent DNA in denaturing agarose gels, dependent on the presence of both Fen1 and ligase (Figures 1B and 1C; the replication reactions contained all of the factors indicated in Figures S1B and S1C), indicating that the reconstituted replisomes traverse the majority of the plasmid template.

Subsequently, however, a more careful analysis indicated that the ligated nascent strands were in fact between 3,000 and 3,100 nt in size (Figure 1D) and were thus about 90–190 nt shorter than the full-length plasmid. These data suggested that the majority of converging replisomes stall during the final stages of DNA replication in the reconstituted system. To explore this further, we resolved the products of similar reactions in native agarose gels. Digestion of complete replication products would produce 3.2-kb linear dsDNA (Figure 1A; Complete Replication). However, the vast majority of the digested reaction products migrated in a native gel as replication intermediates of approximately twice the plasmid size (Figure 1E; +SbfI), indicating that the replicated plasmids were still linked by a short stretch of parental dsDNA (Figure 1A, Termination defect).

Pulse-chase experiments demonstrated that the appearance of these large products was coincident with the generation of leading strands of approximately half unit length (Figure 1F; note that ligase and Fen1 were omitted in this case so that leading-strand synthesis could be monitored in denaturing gels). Overall, therefore, these data indicated that the reaction products represented late replication intermediates (LRIs), which result from stalling of converging replisomes before the final stretch of parental duplex DNA has been unwound.

LRI formation was independent of vector size or sequence (Figures S1A and S1D) and was observed over a range of salt concentrations (Figure S1E). In addition, the LRIs persisted in longer time course experiments (Figure S1F), indicating that they are a terminal product of the replication reaction under these conditions. Because the replisome contains multiple factors that modulate fork progression, such as Mrc1-Tof1-Csm3 (Calzada et al., 2005; Szyjka et al., 2005; Tourrière et al., 2005; Yeeles et al., 2017), we also performed reactions with a "minimal" replisome that lacks these factors, at the same time omitting Pol δ and other proteins that are required for complete lagging-strand synthesis (Yeeles et al., 2015). Again, however, the large majority of converging forks arrested, producing LRIs (Figure S1G). These data indicated that LRI formation is an inherent consequence of fork convergence in the reconstituted replication system.

LRI Formation Does Not Reflect the Encounter of Converging Replisomes with Inactive Mcm2-7 Double Hexamers

Although yeast plasmids are replicated from a single replication origin *in vivo*, Mcm2-7 double hexamers can also be loaded at additional sites during reconstituted reactions with naked DNA templates (Remus et al., 2009) because of the presence of additional "cryptic" binding sites for the origin recognition complex

(E) Similar reactions as those in (C) were performed in the absence of Top2 (to avoid catenation of replication products under these conditions), digested with SbfI as indicated, and then analyzed by native agarose gel electrophoresis (top). Shown at the bottom is the same gel stained with ethidium bromide to illustrate the efficiency of digestion by SbfI. The percentage of full-length products was determined by autoradiography, as described in STAR Methods. LRI, late replication intermediate.

(F) A 9.7-kb plasmid template (pZN3) was replicated for 2 h and 50 min in the presence of ^{32}P-dCTP (deoxycytidine triphosphate) before addition of a chase of cold dNTPs (see STAR Methods for details). Ligase and Fen1 were omitted from the reactions so that unligated leading strands could be monitored in denaturing agarose gels (bottom; we also omitted Pol δ from these reactions). In parallel, aliquots of the same samples were digested with SmaI and monitored in a native gel (top), revealing the accumulation of LRIs at the end of the reaction.

See also Figure S1.

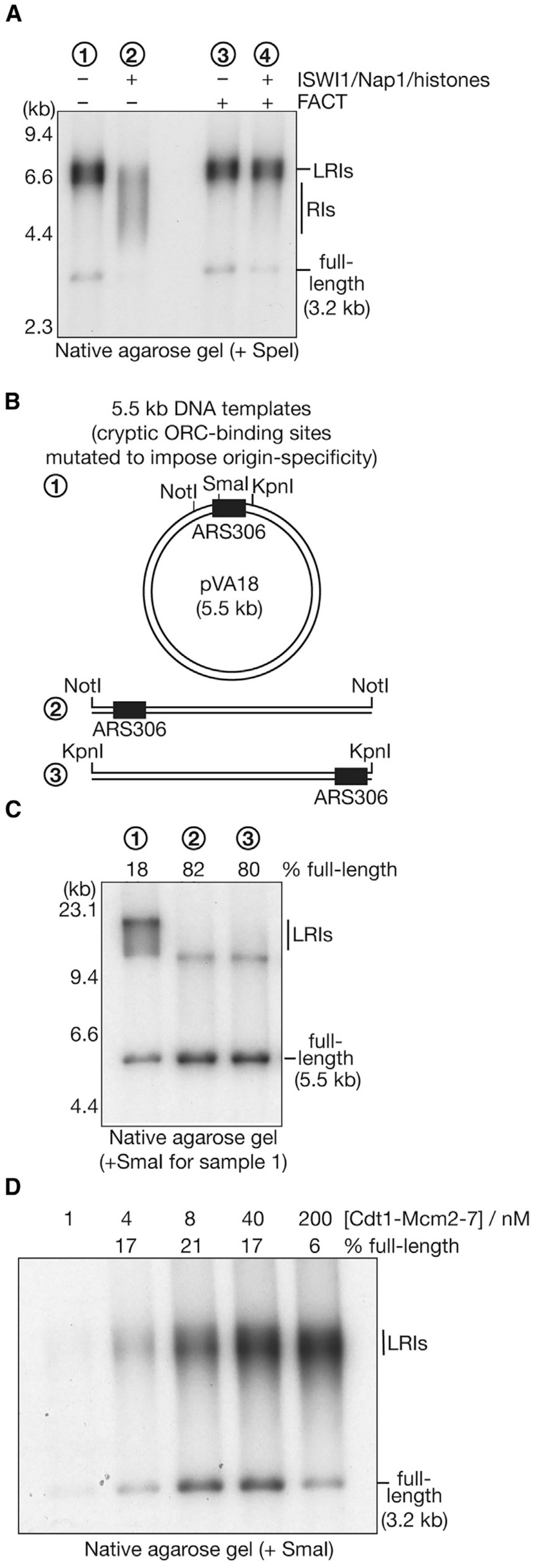

Figure 2. LRI Formation on Plasmids with a Single Origin of Replication

(A) After replication of naked (lanes 1 and 3) or chromatinized (lanes 2 and 4) versions of a 3.2-kb plasmid, the products were digested with SpeI and analyzed in a native agarose gel. Chromatin templates were generated in the presence of ISWI1, Nap1, and histones, and the histone chaperone FACT was included in the replication step as indicated. RIs = replication intermediates.

(B) A 5.5-kb plasmid template was generated that contained the origin *ARS306* but in which other putative binding sites for ORC were mutated so that replication could only initiate from a single site (1). The plasmid was linearized with the indicated enzymes (2 and 3) so that the origin was located at either end of the linearized dsDNA, as indicated.

(C) The DNA templates depicted in (B) were replicated and then analyzed by native agarose gel electrophoresis (sample 1 was digested with SmaI after purifying the replicated DNA to remove catenanes). Pol δ, ligase, and Fen1 were omitted from these reactions so that un-ligated leading strands could be analyzed in denaturing agarose gels (Figure S2B) to monitor origin specificity.

(D) The 3.2-kb plasmid template was replicated in the presence of the indicated concentrations of Cdt1-Mcm2-7 before DNA purification, digestion with SmaI, and analysis by native gel electrophoresis.

See also Figure S2.

(or ORC) (Coster and Diffley, 2017), which are normally suppressed by chromatin (Azmi et al., 2017; Devbhandari et al., 2017; Kurat et al., 2017). This raised the possibility that the two replisomes from one origin might encounter inactive Mcm2-7 double hexamers elsewhere on the plasmid during the course of elongation in our reconstituted replication reactions. Because the inactive Mcm2-7 complexes can slide along dsDNA (Evrin et al., 2009; Gros et al., 2015; Remus et al., 2009), it was conceivable that the double hexamers might be pushed ahead of the two replisomes and form a barrier to fork convergence during DNA replication termination, producing the LRIs. At present, the mechanism by which active forks lead to the displacement of inactive Mcm2-7 double hexamers is not understood.

As a first way of addressing whether an excess of loaded Mcm2-7 complexes contributes to LRI formation, we generated a chromatinized version of the 3.2-kb plasmid template used above (Figure S2A). After loading the Mcm2-7 proteins onto DNA, replication reactions were performed in the presence or absence of the histone chaperone FACT (facilitates chromatin transactions), which is part of the replisome that assembles around the CMG helicase at replication forks (Foltman et al., 2013; Gambus et al., 2006). As seen previously (Kurat et al., 2017), the replication of naked plasmid DNA was independent of FACT (Figure 2A, lanes 1 and 3), but fork progression through chromatin was impeded in the absence of FACT, producing a broad smear of replication intermediates (Figure 2A, lane 2). Importantly, the products of chromatin replication in the presence of FACT were indistinguishable from those seen upon replication of naked DNA, with the majority comprising LRIs (Figure 2A, lane 4). These findings indicated that the defect in fork convergence is still observed when Mcm2-7 loading is restricted to *ARS1* by chromatin assembly.

In a second approach, we used plasmid templates in which the cryptic ORC binding sites were mutated (pZN3 and pVA18; Figure S1A) so that replication initiates specifically from the origin *ARS306*, even in the absence of chromatin (Taylor and Yeeles, 2018). As above, LRIs still formed during DNA replication (pZN3 in Figure 1F; pVA18 in Figure 2C, lane 1). Moreover, LRI

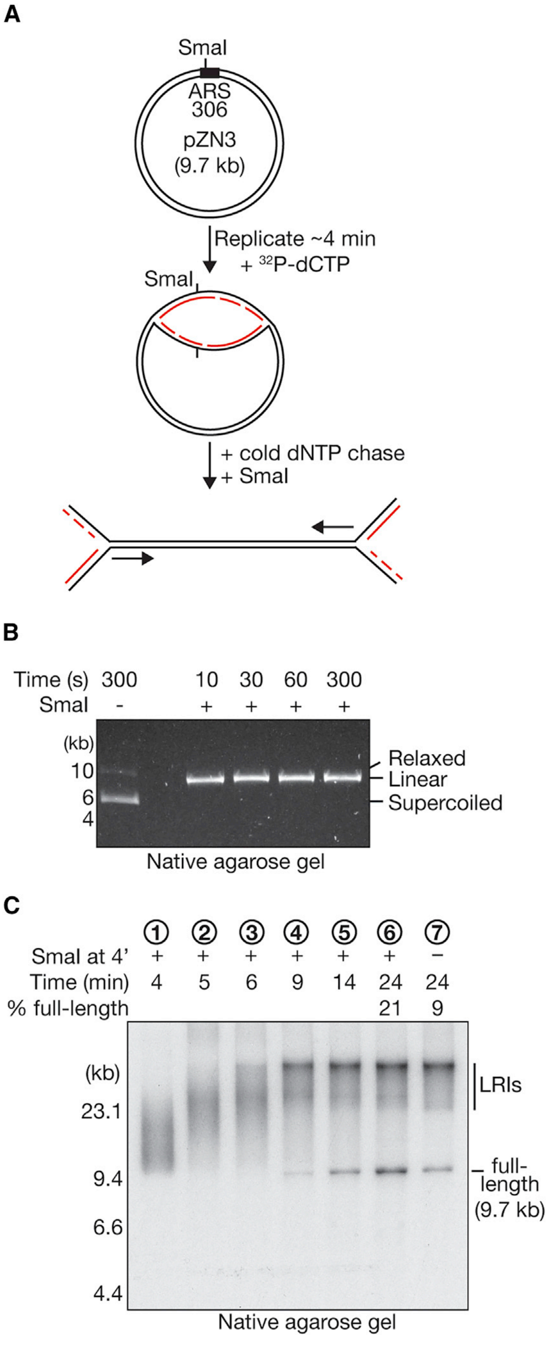

Figure 3. LRI Formation on Linearized Templates

(A) A 9.7-kb plasmid (pZN3) with a single site of Mcm2-7 loading was replicated for 3 min and 50 s in the presence of ^{32}P-dCTP to allow establishment of bi-directional replication forks. A chase of unlabeled dNTPs was then added, followed immediately by addition of SmaI to linearize the vector at the indicated site adjacent to the origin. Nascent DNA strands are shown in red. Note that Pol δ, ligase, and Fen1 were omitted in this experiment.

(B) Control experiment illustrating that SmaI digestion of the 9.7-kb plasmid at 30°C in replication reaction buffer leads to linearization within 10 s. DNA was visualized with Sybr Safe stain.

(C) The 9.7-kb plasmid template was linearized during replication as in (A), and samples were removed and quenched at the indicated times (lanes 1–6). As a control, undigested plasmid was replicated for 24 min in a parallel reaction (lane 7) before extraction of the replicated DNA and digestion with SmaI. The samples were then resolved in a native agarose gel and analyzed by autoradiography.

formation did not reflect an elongation defect under such conditions because the majority of replication products were full-length when the template was linearized close to the origin so that a single replication fork traversed almost the entire length of the plasmid (Figures 2B, 2C, and S2B, samples 2 and 3).

Finally, we investigated the effect of modulating the Mcm2-7 loading conditions for the naked 3.2-kb plasmid template that contained cryptic ORC binding sites in addition to the origin *ARS1*. We varied the concentration of Cdt1 and the Mcm2-7 proteins in the MCM loading reaction because this has been shown to influence the number of Mcm2-7 double hexamers that are loaded onto the DNA template (Douglas et al., 2018). At the highest concentration used, we found that the proportion of full-length reaction products was reduced severalfold (Figure 2D, compare 200 nM and 40 nM Mcm2-7) so that almost all of the replicated molecules comprised LRIs. This suggests that an excess of loaded Mcm2-7 complexes can indeed interfere with DNA replication termination. However, LRIs still represented over 80% of the replication products when the concentration of Mcm2-7 was lowered dramatically, to levels that reduced the efficiency of replication and should only support loading of a single Mcm2-7 double hexamer (Figure 2D, 4 nM Mcm2-7).

Taken together, these findings indicate that LRIs do not result from the encounter of converging replication forks with Mcm2-7 double hexamers nor from a defect in elongation (because an individual fork can replicate the entire length of a linearized template DNA). Instead, LRIs are produced by an inherent problem that arises when two reconstituted replisomes converge during DNA replication termination.

LRI Formation on Linear DNA

We next examined whether the LRI was specifically produced by DNA replication termination on a circular plasmid or would also result from replisome convergence on a linearized template. Using the 9.7-kb origin-specific plasmid (pZN3; Figure S1A), we pulse-labeled the two nascent replication forks for 4 min (Figure 3A). At that point, we added a cold "chase" of deoxyribonucleotide triphosphates (dNTPs) together with the SmaI restriction enzyme, which cleaves the template DNA extremely rapidly under these conditions (Figure 3B), proximal to the origin *ARS306* and, thus, behind the two active replication forks. The progression and convergence of the two forks were then monitored in native agarose gels. Although linearization of the template routinely led to an ~10% increase in the abundance of full-length products (Figure 3C, compare lanes 6 and 7), the LRIs still represented around 80% of the products synthesized under these conditions (Figure 3C, lane 6). Therefore, even when two replisomes approach each other on linearized DNA, the majority still stall upon convergence.

Eukaryotic Pif1-Family DNA Helicases Promote Fork Convergence

Although the convergence of two reconstituted replisomes produces a late replication intermediate, termination is extremely

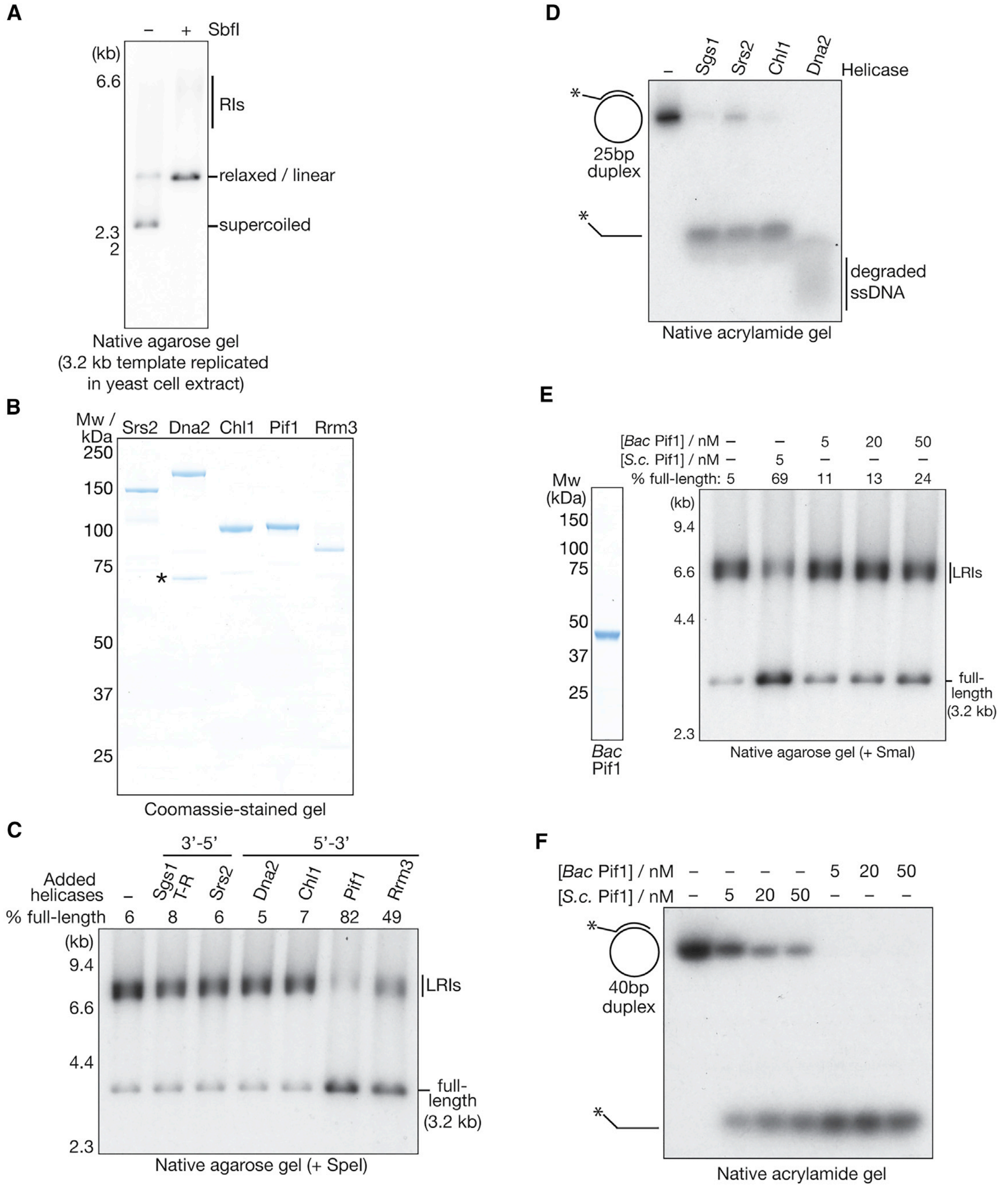

Figure 4. The Budding Yeast Pif1 and Rrm3 DNA Helicases Stimulate Fork Convergence *In Vitro*

(A) Mcm2-7 double hexamers were loaded and phosphorylated on the 3.2-kb plasmid template, which was then replicated in extracts of early S phase yeast cells (see STAR Methods for details). The reaction products were purified and then digested with SbfI as indicated before analysis in a native agarose gel.

(B) The indicated *S. cerevisiae* DNA helicases were purified and then visualized by SDS-PAGE and Coomassie staining. The asterisk denotes a contaminant in the Dna2 sample.

(C) The indicated DNA helicases (see STAR Methods for concentrations) were included in replication reactions with the 3.2-kb plasmid template. The reactions were stopped and quenched after 20 min, and then the replicated products were purified and linearized with SpeI before resolution in a native agarose gel. Pol δ, Fen1, and ligase were omitted from the reactions to prevent strand displacement synthesis by Pol δ, which has been shown previously to be enhanced by Pif1-Rrm3 (Osmundson et al., 2017; Rossi et al., 2008). Sgs1-T-R, Sgs1-Top3-Rmi1.

(D) The ability of the indicated enzymes to unwind a 25-bp DNA duplex, formed by annealing a 35-bp oligonucleotide to an M13 ssDNA template, was monitored as described in STAR Methods.

(legend continued on next page)

efficient when plasmid replication is initiated in yeast cell extracts (On et al., 2014), and around 90% of the linearized reaction products comprise full-length dsDNA (Figure 4A). This suggests that efficient fork convergence relies on additional factors that are present in cells but absent from the purified replication system. Because the formation of LRIs indicates that the two converging replisomes cannot unwind the final stretch of parental dsDNA, we considered whether additional ''accessory'' DNA helicases might be important to stimulate DNA replication termination. Therefore, we expressed and purified a range of budding yeast DNA helicases that have diverse roles in chromosome duplication (Figure 4B) and incorporated them into the reconstituted replication reactions.

Previous studies of DNA replication termination in *E. coli* showed that the RecQ helicase can unwind a late replication intermediate in the presence of Topoisomerase III and single-stranded DNA (ssDNA)-binding-protein, producing two daughter molecules that each have a short stretch of ssDNA (Suski and Marians, 2008). The yeast RecQ ortholog Sgs1 associates with Top3 and another factor known as Rmi1. This complex plays an important role in resolving recombination intermediates, including Holliday junctions (Bizard and Hickson, 2014; Larsen and Hickson, 2013), and can also catalyze the catenation and decatenation of dsDNA (Cejka et al., 2012; Figure S3A). However, addition of Sgs1-Top3-Rmi1 to the replication reactions had no effect on LRI formation, indicating that the yeast RecQ helicase was unable to promote fork convergence under these conditions (Figure 4C; Figures S3B and S3C; note that high concentrations of Sgs1-Top3-Rmi1 partially inhibited DNA replication in the reconstituted system). Similarly, the stalling of converging forks was unaffected by the presence of the anti-recombinase Srs2 (Ira et al., 2003), the Dna2 nuclease-helicase that contributes to DNA repair and Okazaki fragment processing (Stodola and Burgers, 2017), or the Chl1 helicase (Mayer et al., 2004; Samora et al., 2016; Skibbens, 2004) that plays an important but poorly defined role in the establishment of sister chromatid cohesion during chromosome duplication (Figure 4C; Figures S3D–S3F). Importantly, the purified versions of Sgs1 (3′ to 5′), Srs2 (3′ to 5′), and Chl1 (5′ to 3′) were all active as DNA helicases *in vitro* (Figure 4D; purified Dna2 was active as a nuclease, which complicated analysis of its 5′ to 3′ helicase activity).

The budding yeast Pif1 and Rrm3 proteins are paralogs with 5′ to 3′ DNA helicase activity and partially overlapping functions during elongation. Pif1 and Rrm3 jointly help the replisome to bypass barriers at many sites around the genome, such as tRNA promoters, where non-nucleosomal proteins bind tightly to DNA (Ivessa et al., 2002, 2003; Osmundson et al., 2017; Tran et al., 2017). Strikingly, addition of Pif1 to the reconstituted replication reactions greatly stimulated the formation of full-length dsDNA, with a corresponding reduction in the appearance of LRIs (Figure 4C; Figure S3G). Moreover, Pif1 could also suppress LRI formation on chromatinized or linearized DNA templates (Figures S4A and S4B). Similarly, the Rrm3 helicase also stimulated the formation of full-length molecules in analogous reactions (Figure 4C; Figure S3H). However, a bacterial ortholog of Pif1-Rrm3 was much less effective at supporting fork convergence in the reconstituted yeast replication system (Figure 4E), even when added at a 10-fold higher concentration than yeast Pif1, despite being highly active as a DNA helicase (Figure 4F; note that *Bac* Pif1 is better able to unwind a 40-bp duplex than *S.c.* Pif1). These findings indicate that the budding yeast Pif1 and Rrm3 helicases have a specific ability to promote the convergence of yeast replisomes.

The addition of Pif1 and Rrm3 to the reconstituted replication reactions did not affect the rate of elongation (Figure S5A), and replication was still dependent on the CMG helicase (Figures S5B and S5C). To test directly whether the Pif1 and Rrm3 helicases can promote fork convergence, we examined the ability of wild-type or helicase-dead mutants of Pif1 and Rrm3 (Figures 5A and 5B) to resolve pre-formed LRIs. We generated LRIs as above in the absence of Pif1-Rrm3 and then added wild-type or mutant versions of Pif1 or Rrm3 together with a cold chase of unlabeled dNTPs. As shown in Figures 5C and 5D, both Pif1 and Rrm3 were able to resolve pre-formed LRIs and stimulate the production of full-length dsDNA, dependent on their helicase activity. Moreover, Pif1 could still unwind pre-formed LRIs in the presence of the DNA polymerase inhibitor aphidicolin (Figure 5E; Rrm3 was not tested in this assay) at doses that potently inhibited DNA synthesis in the reconstituted replication system (Figure 5F). In summary, these findings indicate that the Pif1 and Rrm3 helicases are able to support fork convergence during DNA replication termination by helping to unwind the final stretch of parental DNA. Furthermore, these data demonstrate that the LRIs are *bona fide* precursors for full-length replication products.

Pif1 and Rrm3 Support a Top2-Independent Pathway for DNA Replication Termination

In *E.coli*, the type II topoisomerase TopoIV stimulates DNA replication termination by removing precatenanes behind the converging DNA replication forks, reducing torsional strain and allowing the final stages of DNA unwinding to proceed (Hiasa and Marians, 1996). To investigate whether type II topoisomerases contribute to fork convergence in the reconstituted yeast replication system, we compared the ability of yeast Top1 or Top2 and *E. coli* TopoIV to promote DNA replication termination. In the absence of any topoisomerase, forks stalled during elongation (Figure S6A, lane 1) because of the accumulation of positive supercoils in front of each replication fork (Yeeles et al., 2015). Although all three of the tested topoisomerases were able to support fork progression during elongation, the majority of reaction products were LRIs in all cases (Figure S6A, lanes 2–5). However, slightly more full-length molecules were

(E) *Bacteroides* Pif1 was expressed and purified as described in STAR Methods and then visualized by SDS-PAGE and Coomassie staining (left). The 3.2-kb plasmid template was then replicated in the presence of 5 nM yeast Pif1 (*S.c.* [*Saccharomyces cerevisiae*]) or the indicated concentrations of *Bacteroides* Pif1 (*Bac*Pif1). The reaction products were purified, digested, and analyzed in native agarose gels.

(F) The ability of the indicated concentrations of *Bac* Pif1 or *S.c.* Pif1 to unwind a 40-bp DNA duplex, formed by annealing a 55-bp oligonucleotide to an M13 ssDNA template, was monitored as above.

See also Figures S3 and S4.

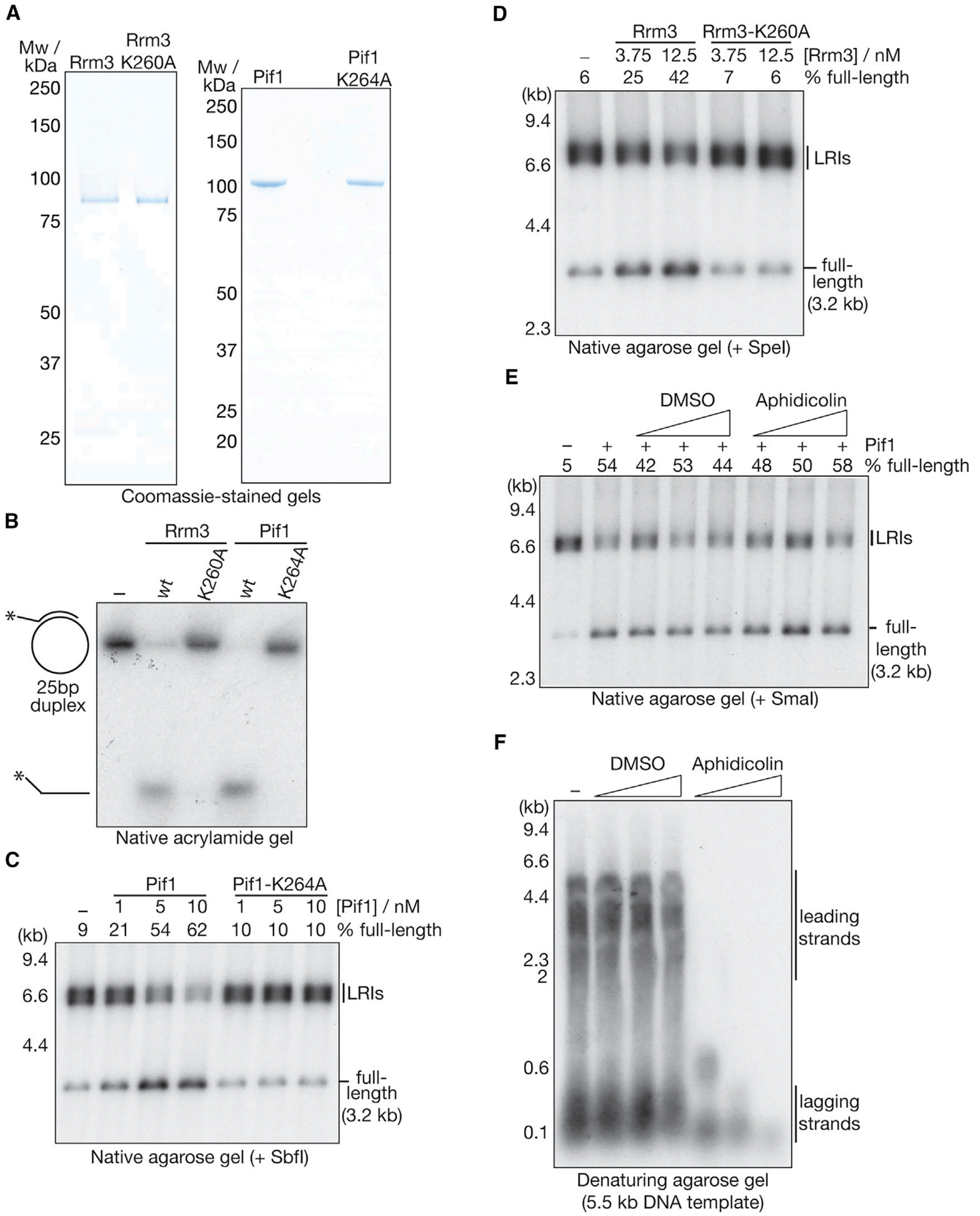

Figure 5. Pif1-Rrm3 Helicase Activity Supports LRI Resolution

(A) Wild-type or ATP-binding mutants of Pif1 and Rrm3 were purified and then visualized by SDS-PAGE and Coomassie staining.

(B) The ability of wild-type or helicase-dead versions of Rrm3 and Pif1 to unwind a 25-bp DNA duplex was monitored as in Figure 4.

(C) LRIs were generated as above before addition of the indicated concentrations of wild-type PIf1 or a helicase-dead variant (Pif1-K264A) together with a cold chase of unlabeled dNTPs. The products were digested with SbfI before analysis in a native agarose gel.

(D) Analogous reactions with wild-type Rrm3 or a helicase-dead equivalent (Rrm3-K260A). Products were digested with SpeI before native gel analysis.

(E) The ability of Pif1 to unwind pre-formed LRIs was monitored in pulse-chase experiments as in (C) and (D) in the presence of DMSO or aphidicolin (10, 40, and 100 μg/mL) as indicated.

(F) The effect of DMSO or aphidicolin (4, 10, and 40 μg/ mL) on DNA synthesis of a 5.5-kb template was monitored by denaturing agarose gel electrophoresis in reactions that lacked Pol δ, ligase, and Fen1.

See also Figure S5.

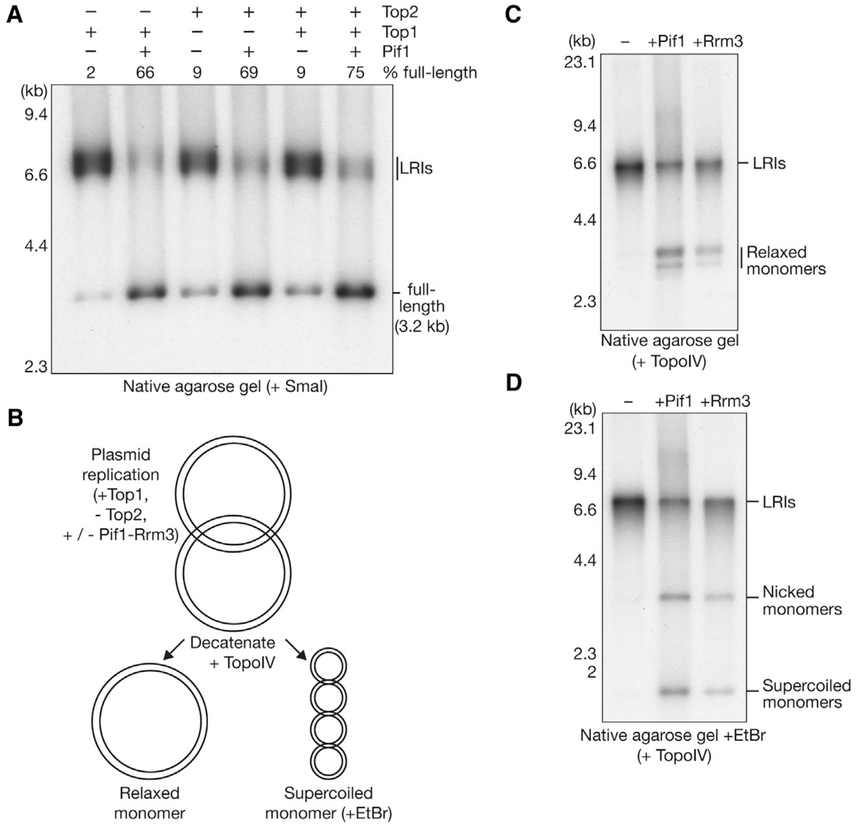

Figure 6. Pif1-Family Helicases Support a Top2-Independent Pathway for DNA Replication Termination

(A) The 3.2-kb plasmid template was replicated in the presence of the indicated topoisomerases with or without Pif1. The products were digested with SmaI before analysis in a native agarose gel.

(B) Reaction schematic for experiments to test whether Pif1 and Rrm3 can support complete plasmid duplication in the absence of Top2. Reactions were performed in the presence of Pol δ, Fen1, ligase, and Top1 with or without Pif1-Rrm3. Complete plasmid replication would produce catenated dimers, which could then be purified and decatenated by *E. coli* TopoIV.

(C) Reactions performed as in (B) were analyzed in native agarose gels in the absence of the DNA intercalator ethidium bromide.

(D) The same reaction products were resolved in a native agarose gel in the presence of 0.5 μg/mL ethidium bromide.

See also Figure S6.

produced in the presence of the type II topoisomerases Top2 or TopoIV (Figures S6A, lanes 3–5, and S6B) compared with a reaction containing the type I enzyme Top1 (Figures S6A, lane 2, and S6B). These findings indicate that type II topoisomerase activity has a modest ability, under these conditions, to promote fork convergence in the absence of Pif1-Rrm3.

To investigate whether Pif1-family helicases promote DNA replication termination independent of type II topoisomerase activity, we compared the ability of Pif1 to drive fork convergence in the presence of Top1, Top2, or both enzymes (Figure 6A). The stimulation of fork convergence by Pif1 was comparable in the presence of either Top1 or Top2 (Figure 6A; Figure S6C), indicating that the action of a type II topoisomerase is not required for Pif1 to promote fork convergence. Nevertheless, the production of full-length products was reproducibly enhanced in reactions containing Pif1 together with both Top1 and Top2 (Figures 6A and S6B).

Finally, we tested whether Pif1 and Rrm3 can support the completion of DNA replication termination and the production of completely ligated products in the absence of type II topoisomerase activity (reactions containing Top1 but not Top2). If plasmid replication were complete under such conditions, then the products should represent catenated dimers, which could then be decatenated with *E. coli* TopoIV after purification of the replicated DNA (Figure 6B). This would produce relaxed monomers, into which negative supercoils could then be introduced by incubation with the DNA intercalator ethidium bromide. As shown in Figure 6C, decatenation of the products of reactions containing Pif1 or Rrm3 did indeed produce relaxed monomers (note that the reaction products were not linearized in these experiments, in contrast to those above). Moreover, a proportion of the reaction products were converted into supercoiled monomers in the presence of ethidium bromide, indicating that the products comprised covalently closed circles (Figure 6D). Therefore, both Pif1 and Rrm3 are able to drive fork convergence and the completion of DNA synthesis in the complete absence of topoisomerase II activity.

Rrm3 and Pif1 Are Required for Efficient Termination of Plasmid Replication *In Vivo*

To explore the *in vivo* significance of the Pif1 helicase family for DNA replication termination, we used native agarose gels and Southern blotting to screen for the appearance of LRIs during plasmid replication in synchronized yeast cells. Previous work showed that LRIs accumulated transiently when fork convergence was impeded in budding yeast cells by expression of a catalytically dead form of Top2 (Baxter and Diffley, 2008). Although LRIs were not observed during plasmid replication in control cells, they accumulated strikingly in *rrm3Δ pif1-m2* double mutant cells before being resolved at later time points (Figures 7A and 7B; note that *rrm3Δ pif1Δ* cells were too sick to examine in similar experiments, and so we used the *pif1-m2* allele, which expresses the mitochondrial isoform of the protein but lacks nuclear Pif1). In contrast, LRI accumulation was less severe in *rrm3Δ* and absent in *pif1Δ* single mutant cells (Figure S7A), indicating that the Rrm3 and Pif1 DNA helicases play a partially redundant role in promoting fork convergence during DNA replication termination in budding yeast cells.

To confirm that the LRIs observed in yeast cells lacking Pif1 and Rrm3 were caused by a specific defect in fork convergence during DNA replication termination rather than being produced

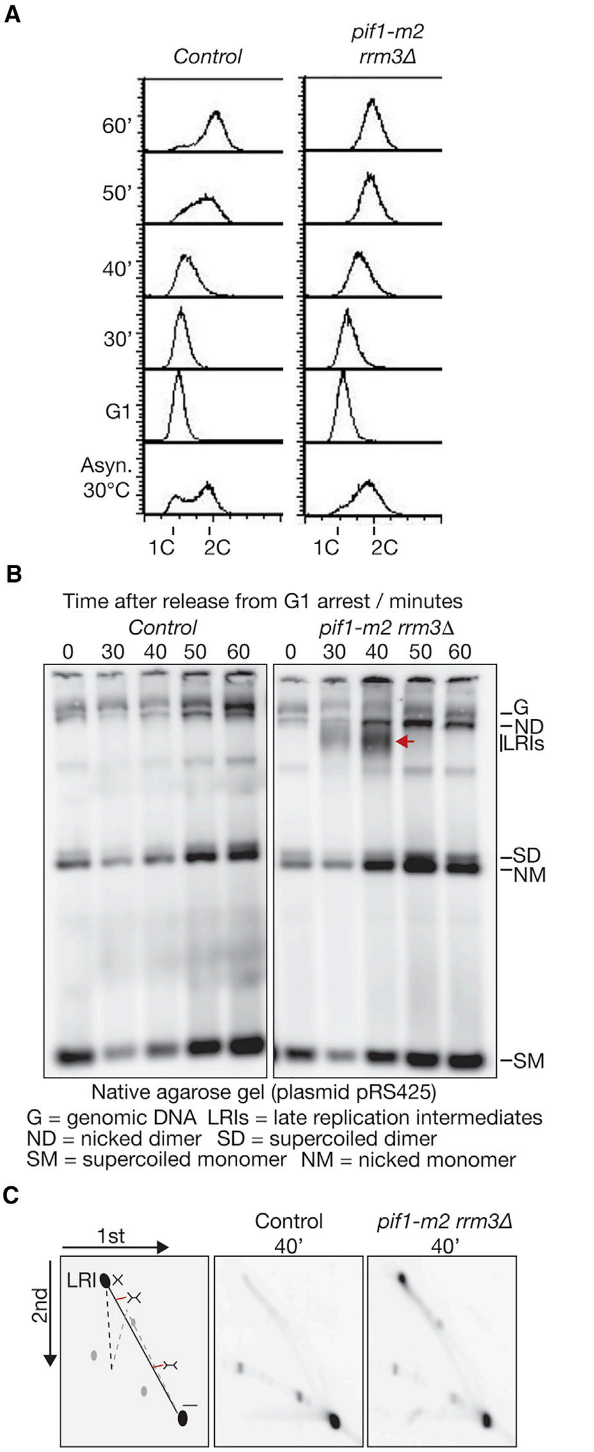

Figure 7. Pif1-Rrm3 Are Required for Efficient Termination of Plasmid DNA Replication *In Vivo*

(A) Control (W303-1a) and *pif1-m2 rrm3Δ* (yMO291) yeast strains containing the plasmid pRS425 were arrested in G1 phase with mating pheromone and then released into S phase for the indicated times. DNA content was monitored by flow cytometry.

(B) Samples from each time point were used to prepare total DNA, which was then analyzed by native agarose gel electrophoresis and Southern blotting (Brewer and Fangman, 1987). The positions of various plasmid isoforms are indicated, and the red arrow indicates the accumulation of LRIs in *pif1-m2 rrm3Δ* cells.

(C) The DNA samples from the 40-min time point in (B) were digested at the origin of replication with SnaBI before resolution in a two-dimensional neutral-neutral agarose gel, as described in STAR Methods. Left: the positions of linear molecules (black dot, bottom right) and replication intermediates (double Y structures of increasing size, located along the line from the linear dot to the LRI).

See also Figure S7.

by the pausing of forks at replication fork barriers, we explored the nature of the LRI by native-native two-dimensional gel electrophoresis. DNA isolated from the 40-min time point in the experiment in Figure 7B was digested with a restriction enzyme that cuts within the origin (Figure S7B), and the samples were then analyzed by native-native two-dimensional gel electrophoresis. If cells lacking Pif1 and Rrm3 have a specific defect in fork convergence during termination, then the digested LRI should comprise a large "double Y" structure that approximates an X-shaped molecule with arms of roughly equal length (LRI in Figure 7C, left, and Figure S7B). However, if the LRI is formed by one fork stalling at a barrier during elongation until the arrival of the second fork, then the digestion of the plasmid at the origin would produce an X-shaped LRI with uneven arm length, which would migrate further down the "X arc" in the two-dimensional gel (Figure 7C, left, black dotted line). In contrast to plasmid DNA from control cells (Figure 7C, center), the digested DNA sample from *pif1-m2 rrm3Δ* cells contained a single LRI species that migrated as a very large double Y near the top of the X arc (Figure 7C, right). This demonstrates that the LRI was produced by a defect in fork convergence on the opposite side of the plasmid to the origin.

DISCUSSION

We reconstituted the final stages of eukaryotic DNA replication and found that the majority of converging replisomes stall when CMG is the only DNA helicase in the reaction, producing late replication intermediates in which the ligated nascent strands are 90–190 bp shorter than the full-length plasmid (Figure 1D). The deficit in nascent strand size reflects the ~30-bp footprint of the CMG helicase at each fork (Räschle et al., 2008), together with any parental dsDNA between the converged replisomes, and also the gap between the 5′ end of the lagging strand and the 3′ end of the leading strand at each fork (Figure 1A). The observed difference between the size of ligated nascent strands and the full-length plasmid is comparable with the situation when two replisomes converge at an inter-strand DNA cross-link in *Xenopus* egg extracts (Räschle et al., 2008). Therefore, the reconstituted yeast replisomes must be very close to each other in the LRI, and the remaining amount of parental dsDNA must be less than the minimal value of about ~150 bp that is needed to form a loop or supercoil (Vafabakhsh and Ha, 2012). This implies that the converging replisomes stall after the point at which fork progression becomes dependent on replisome rotation and precatenane formation.

The defect in fork convergence is seen in the presence of type I or II topoisomerases, regardless of whether the reactions involve

"minimal" (Yeeles et al., 2015) or "complete" (Yeeles et al., 2017) versions of the replisome or whether fork convergence occurs on circular or linearized DNA templates (Figure 3). Several potential explanations for LRI formation could be envisaged. First, the two replisomes might clash when they encounter each other. However, previous work indicated that the CMG helicase encircles the template of the leading strand and can bypass protein barriers on the lagging strand (Fu et al., 2011; Langston et al., 2017). Furthermore, recent evidence indicates that the activation of an Mcm2-7 double hexamer during initiation produces two "converged" CMG helicases that must bypass each other before leaving the origin and establishing bi-directional replication forks (Douglas et al., 2018). This suggests that two converging replisomes should also be able to pass each other unimpeded during DNA replication termination.

A second potential explanation for LRI formation was that inactive Mcm2-7 double hexamers might block the convergence of two replisomes in the absence of factors (potentially including Pif1-Rrm3) that help forks to displace such "pre-replicative" complexes. However, LRIs are still seen under conditions where only a single double hexamer is loaded per plasmid, and our data indicate that the major defect in fork convergence in the reconstituted replication systems arises for another reason, although our findings do not exclude that inactive Mcm2-7 double hexamers have the potential to block progression of the CMG helicase.

A further possibility is that converging CMG helicases become arrested at a stage when continued replisome rotation is impaired by torsional strain. During DNA replication termination in *E. coli* and SV40, the removal of precatenanes by type II topoisomerases relieves torsional strain at converging forks and allows continued replisome rotation and completion of DNA unwinding. We note that Top2 does make a minor contribution to the efficiency of fork convergence in the reconstituted yeast replication system (Figure S6), but depletion of type II topoisomerase does not block fork convergence in yeast cells (Baxter and Diffley, 2008) or *Xenopus* egg extracts (Dewar et al., 2015). This indicates that eukaryotic cells have other pathways that can drive fork convergence during DNA replication termination, independent of type II topoisomerase activity. Our data identify one such pathway that is driven by the Pif1 helicase family and is distinct from the previously described prokaryotic mechanism of fork convergence.

The CMG helicase is essential for replisome progression and can unwind the DNA template over many kilobases, aided by stable entrapment of the leading-strand DNA template within the hexameric Mcm2-7 ring. In contrast, Pif1 and Rrm3 are monomeric DNA helicases that have low processivity (Byrd and Raney, 2017; Singleton et al., 2007) and play important roles at specific points during elongation, unwinding highly stable structures, such as G4 quadruplexes, that might otherwise present a barrier to CMG (Paeschke et al., 2013; Paeschke et al., 2011), and assisting with the replication of centromeres (Chen et al., 2018). It is possible that the robust helicase activity of Pif1-Rrm3 helps to overcome torsional strain at converging forks, driving replisome rotation and helping to unwind the final stretch of parental dsDNA.

The polarity of Pif1 helicases is likely to be an important determinant of their ability to assist with DNA unwinding at converging replication forks. Both Pif1 and Rrm3 unwind DNA 5′ to 3′ and, thus, interact with the template of the lagging strand, complementing the 3′ to 5′ action of the CMG helicase along the leading-strand template. Notably, a bacterial Pif1 helicase was very inefficient at LRI resolution despite being a highly active DNA helicase *in vitro* (Figures 4E and 4F). Moreover, the Chl1 5′ to 3′ helicase was also unable to promote fork convergence in the reconstituted system (Figures 4C and 4D). These findings indicate that the budding yeast Pif1 helicases have a specific ability to support DNA replication termination, perhaps aided by a direct interaction with the budding yeast replisome. Consistent with this view, Rrm3 was found to interact with the catalytic subunit of Pol ε (Azvolinsky et al., 2006).

In agreement with our *in vitro* data, Pif1 and Rrm3 share a partially overlapping role during the termination of plasmid replication in budding yeast cells, leading to the accumulation of LRIs in *rrm3Δ pif1-m2* cells (Figure 7; Figure S7). Fork convergence is delayed but not abolished under such conditions, indicating that additional pathways contribute to termination *in vivo*, possibly including the removal of precatenanes by type II topoisomerases (Fachinetti et al., 2010). The existence of multiple pathways that promote fork convergence likely explains why budding yeast cells lacking both Pif1 and Rrm3 are very sick but able to form colonies. Functional redundancy with other fork convergence pathways might also explain the viability of mice that lack the single mammalian ortholog of Pif1 (Snow et al., 2007). However, we cannot exclude that a penetrant defect in fork convergence in the absence of Pif1 helicases is counterbalanced by other pathways that can resolve X-shaped DNA molecules before cell division; for example, through the action of nucleases and recombination factors.

We note that our data are consistent with past studies that monitored fork convergence at protein-DNA barriers *in vivo* and that observed termination defects in budding yeast cells lacking Rrm3 (Ivessa et al., 2000; Mohanty et al., 2006) or in fission yeast cells lacking the Pfh1 ortholog of Pif1-Rrm3 (Sabouri et al., 2012; Steinacher et al., 2012). However, our findings establish that Pif1 and Rrm3 have a direct role in fork convergence per se rather than simply being required to remove a protein-DNA barrier that otherwise would block fork convergence.

The reconstitution of Pif1-dependent DNA replication termination sets the scene for future mechanistic studies of this enigmatic and fascinating area of chromosome duplication. It will be particularly interesting to determine how the termination of DNA synthesis provides a signal for disassembly of the eukaryotic replisome, which is initiated by ubiquitylation of the CMG helicase and represents the final regulated step in eukaryotic chromosome replication (Dewar et al., 2015, 2017; Maric et al., 2014; Moreno et al., 2014; Sonneville et al., 2017).

STAR★METHODS

Detailed methods are provided in the online version of this paper and include the following:

- KEY RESOURCES TABLE
- CONTACT FOR REAGENT AND RESOURCE SHARING
- EXPERIMENTAL MODEL AND SUBJECT DETAILS

- METHOD DETAILS
  - Yeast methods
  - Protein purification buffers
  - Protein purification
  - DNA templates
  - Molecular weight markers
  - *In vitro* replication assays
  - Preparation of substrates for helicase assays
  - Helicase assays
  - Catenation assay with Sgs1-Top3-Rmi1
  - DNA preparation for agarose gel electrophoresis
  - Decatenation of replicated plasmids with *E. coli* TopoIV
  - One-dimensional agarose gel electrophoresis and Southern blotting
  - Two-dimensional native-native agarose gel electrophoresis
  - Gel imaging and presentation
- QUANTIFICATION AND STATISTICAL ANALYSIS

SUPPLEMENTAL INFORMATION

Supplemental Information can be found with this article online at https://doi.org/10.1016/j.molcel.2019.01.040.

ACKNOWLEDGMENTS

We gratefully acknowledge support from the Wellcome Trust (reference 204678/Z/16/Z for a Sir Henry Wellcome postdoctoral fellowship to T.D.D. and 102943/Z/13/Z for an investigator award to K.P.M.L.), the Medical Research Council (core grants MC_UU_12016/13 to K.P.M.L. and MC_UP_1201/12 to J.T.P.Y.), BBSRC (project grant BB/N007344/1 to J.B.), and the Royal Society (a research fellowship to J.B.). We thank Valentina Aria for plasmid pVA18, John Diffley for strains and for sharing unpublished data, Steve Kowalczykowski for purified Sgs1 and Top3-Rmi1, Lumir Krejci for plasmids and protocols for expression of Srs2, Martin Singleton for providing baculoviruses that express Chl1, Fabrizio Villa for help with insect cell growth and infection, and MRC PPU Reagents and Services (https://mrcppureagents.dundee.ac.uk) for support.

AUTHOR CONTRIBUTIONS

J.B. analyzed plasmid replication in budding yeast cells (Figures 7 and S7), M.A.O.B. constructed and characterized the *pif1-m2 rrm3Δ* strains, and T.D.D. performed all other experiments. J.T.P.Y. supplied the origin-specific plasmids pZN3 and pVA18, reagents for chromatin replication, and invaluable advice and support. T.D.D. and K.P.M.L. designed the project together with J.B. The manuscript was written by T.D.D. and K.P.M.L. with invaluable input from J.B. and J.T.P.Y.

DECLARATION OF INTERESTS

The authors declare no competing interests.

Received: August 9, 2018
Revised: November 26, 2018
Accepted: January 29, 2019
Published: March 5, 2019

REFERENCES

Azmi, I.F., Watanabe, S., Maloney, M.F., Kang, S., Belsky, J.A., MacAlpine, D.M., Peterson, C.L., and Bell, S.P. (2017). Nucleosomes influence multiple steps during replication initiation. eLife *6*, e22512.

Azvolinsky, A., Dunaway, S., Torres, J.Z., Bessler, J.B., and Zakian, V.A. (2006). The S. cerevisiae Rrm3p DNA helicase moves with the replication fork and affects replication of all yeast chromosomes. Genes Dev. *20*, 3104–3116.

Baxter, J., and Diffley, J.F.X. (2008). Topoisomerase II inactivation prevents the completion of DNA replication in budding yeast. Mol. Cell *30*, 790–802.

Bell, S.P., and Labib, K. (2016). Chromosome Duplication in Saccharomyces cerevisiae. Genetics *203*, 1027–1067.

Bermejo, R., Doksani, Y., Capra, T., Katou, Y.M., Tanaka, H., Shirahige, K., and Foiani, M. (2007). Top1- and Top2-mediated topological transitions at replication forks ensure fork progression and stability and prevent DNA damage checkpoint activation. Genes Dev. *21*, 1921–1936.

Biswas, D., Yu, Y., Prall, M., Formosa, T., and Stillman, D.J. (2005). The yeast FACT complex has a role in transcriptional initiation. Mol. Cell. Biol. *25*, 5812–5822.

Bizard, A.H., and Hickson, I.D. (2014). The dissolution of double Holliday junctions. Cold Spring Harb. Perspect. Biol. *6*, a016477.

Brewer, B.J., and Fangman, W.L. (1987). The localization of replication origins on ARS plasmids in S. cerevisiae. Cell *51*, 463–471.

Burgers, P.M.J., and Kunkel, T.A. (2017). Eukaryotic DNA Replication Fork. Annu. Rev. Biochem. *86*, 417–438.

Byrd, A.K., and Raney, K.D. (2017). Structure and function of Pif1 helicase. Biochem. Soc. Trans. *45*, 1159–1171.

Calzada, A., Hodgson, B., Kanemaki, M., Bueno, A., and Labib, K. (2005). Molecular anatomy and regulation of a stable replisome at a paused eukaryotic DNA replication fork. Genes Dev. *19*, 1905–1919.

Cejka, P., Plank, J.L., Dombrowski, C.C., and Kowalczykowski, S.C. (2012). Decatenation of DNA by the S. cerevisiae Sgs1-Top3-Rmi1 and RPA complex: a mechanism for disentangling chromosomes. Mol. Cell *47*, 886–896.

Chen, C.F., Pohl, T.J., Pott, S., and Zakian, V.A. (2018). Two Pif1-family DNA Helicases Cooperate in Centromere Replication and Segregation in Saccharomyces cerevisiae. Genetics *211*, 1105–1119.

Christianson, T.W., Sikorski, R.S., Dante, M., Shero, J.H., and Hieter, P. (1992). Multifunctional yeast high-copy-number shuttle vectors. Gene *110*, 119–122.

Coster, G., and Diffley, J.F.X. (2017). Bidirectional eukaryotic DNA replication is established by quasi-symmetrical helicase loading. Science *357*, 314–318.

Coster, G., Frigola, J., Beuron, F., Morris, E.P., and Diffley, J.F. (2014). Origin licensing requires ATP binding and hydrolysis by the MCM replicative helicase. Mol. Cell *55*, 666–677.

Deegan, T.D., and Diffley, J.F. (2016). MCM: one ring to rule them all. Curr. Opin. Struct. Biol. *37*, 145–151.

Devbhandari, S., Jiang, J., Kumar, C., Whitehouse, I., and Remus, D. (2017). Chromatin Constrains the Initiation and Elongation of DNA Replication. Mol. Cell *65*, 131–141.

Dewar, J.M., and Walter, J.C. (2017). Mechanisms of DNA replication termination. Nat. Rev. Mol. Cell Biol. *18*, 507–516.

Dewar, J.M., Budzowska, M., and Walter, J.C. (2015). The mechanism of DNA replication termination in vertebrates. Nature *525*, 345–350.

Dewar, J.M., Low, E., Mann, M., Räschle, M., and Walter, J.C. (2017). CRL2^{Lrr1} promotes unloading of the vertebrate replisome from chromatin during replication termination. Genes Dev. *31*, 275–290.

Douglas, M.E., Ali, F.A., Costa, A., and Diffley, J.F.X. (2018). The mechanism of eukaryotic CMG helicase activation. Nature *555*, 265–268.

Evrin, C., Clarke, P., Zech, J., Lurz, R., Sun, J., Uhle, S., Li, H., Stillman, B., and Speck, C. (2009). A double-hexameric MCM2-7 complex is loaded onto origin DNA during licensing of eukaryotic DNA replication. Proc. Natl. Acad. Sci. USA *106*, 20240–20245.

Fachinetti, D., Bermejo, R., Cocito, A., Minardi, S., Katou, Y., Kanoh, Y., Shirahige, K., Azvolinsky, A., Zakian, V.A., and Foiani, M. (2010). Replication termination at eukaryotic chromosomes is mediated by Top2 and occurs at genomic loci containing pausing elements. Mol. Cell *39*, 595–605.

Foltman, M., Evrin, C., De Piccoli, G., Jones, R.C., Edmondson, R.D., Katou, Y., Nakato, R., Shirahige, K., and Labib, K. (2013). Eukaryotic replisome components cooperate to process histones during chromosome replication. Cell Rep. *3*, 892–904.

Frigola, J., Remus, D., Mehanna, A., and Diffley, J.F. (2013). ATPase-dependent quality control of DNA replication origin licensing. Nature *495*, 339–343.

Fu, Y.V., Yardimci, H., Long, D.T., Ho, T.V., Guainazzi, A., Bermudez, V.P., Hurwitz, J., van Oijen, A., Schärer, O.D., and Walter, J.C. (2011). Selective bypass of a lagging strand roadblock by the eukaryotic replicative DNA helicase. Cell *146*, 931–941.

Gambus, A., Jones, R.C., Sanchez-Diaz, A., Kanemaki, M., van Deursen, F., Edmondson, R.D., and Labib, K. (2006). GINS maintains association of Cdc45 with MCM in replisome progression complexes at eukaryotic DNA replication forks. Nat. Cell Biol. *8*, 358–366.

Gros, J., Kumar, C., Lynch, G., Yadav, T., Whitehouse, I., and Remus, D. (2015). Post-licensing Specification of Eukaryotic Replication Origins by Facilitated Mcm2-7 Sliding along DNA. Mol. Cell *60*, 797–807.

Hiasa, H., and Marians, K.J. (1996). Two distinct modes of strand unlinking during theta-type DNA replication. J. Biol. Chem. *271*, 21529–21535.

Ira, G., Malkova, A., Liberi, G., Foiani, M., and Haber, J.E. (2003). Srs2 and Sgs1-Top3 suppress crossovers during double-strand break repair in yeast. Cell *115*, 401–411.

Ishimi, Y., Sugasawa, K., Hanaoka, F., Eki, T., and Hurwitz, J. (1992). Topoisomerase II plays an essential role as a swivelase in the late stage of SV40 chromosome replication in vitro. J. Biol. Chem. *267*, 462–466.

Ivessa, A.S., Zhou, J.Q., and Zakian, V.A. (2000). The Saccharomyces Pif1p DNA helicase and the highly related Rrm3p have opposite effects on replication fork progression in ribosomal DNA. Cell *100*, 479–489.

Ivessa, A.S., Zhou, J.Q., Schulz, V.P., Monson, E.K., and Zakian, V.A. (2002). Saccharomyces Rrm3p, a 5′ to 3′ DNA helicase that promotes replication fork progression through telomeric and subtelomeric DNA. Genes Dev. *16*, 1383–1396.

Ivessa, A.S., Lenzmeier, B.A., Bessler, J.B., Goudsouzian, L.K., Schnakenberg, S.L., and Zakian, V.A. (2003). The Saccharomyces cerevisiae helicase Rrm3p facilitates replication past nonhistone protein-DNA complexes. Mol. Cell *12*, 1525–1536.

Keszthelyi, A., Minchell, N.E., and Baxter, J. (2016). The Causes and Consequences of Topological Stress during DNA Replication. Genes (Basel) *7*, 134.

Kingston, I.J., Yung, J.S., and Singleton, M.R. (2011). Biophysical characterization of the centromere-specific nucleosome from budding yeast. J. Biol. Chem. *286*, 4021–4026.

Kurat, C.F., Yeeles, J.T.P., Patel, H., Early, A., and Diffley, J.F.X. (2017). Chromatin Controls DNA Replication Origin Selection, Lagging-Strand Synthesis, and Replication Fork Rates. Mol. Cell *65*, 117–130.

Labib, K., Diffley, J.F.X., and Kearsey, S.E. (1999). G1-phase and B-type cyclins exclude the DNA-replication factor Mcm4 from the nucleus. Nat. Cell Biol. *1*, 415–422.

Langston, L.D., Mayle, R., Schauer, G.D., Yurieva, O., Zhang, D., Yao, N.Y., Georgescu, R.E., and O'Donnell, M.E. (2017). Mcm10 promotes rapid isomerization of CMG-DNA for replisome bypass of lagging strand DNA blocks. eLife *6*, e29118.

Larsen, N.B., and Hickson, I.D. (2013). RecQ Helicases: Conserved Guardians of Genomic Integrity. Adv. Exp. Med. Biol. *767*, 161–184.

Lucas, I., Germe, T., Chevrier-Miller, M., and Hyrien, O. (2001). Topoisomerase II can unlink replicating DNA by precatenane removal. EMBO J. *20*, 6509–6519.

Marahrens, Y., and Stillman, B. (1992). A yeast chromosomal origin of DNA replication defined by multiple functional elements. Science *255*, 817–823.

Maric, M., Maculins, T., De Piccoli, G., and Labib, K. (2014). Cdc48 and a ubiquitin ligase drive disassembly of the CMG helicase at the end of DNA replication. Science *346*, 1253596.

Maric, M., Mukherjee, P., Tatham, M.H., Hay, R., and Labib, K. (2017). Ufd1-Npl4 Recruit Cdc48 for Disassembly of Ubiquitylated CMG Helicase at the End of Chromosome Replication. Cell Rep. *18*, 3033–3042.

Marini, V., and Krejci, L. (2012). Unwinding of synthetic replication and recombination substrates by Srs2. DNA Repair (Amst) *11*, 789–798.

Mayer, M.L., Pot, I., Chang, M., Xu, H., Aneliunas, V., Kwok, T., Newitt, R., Aebersold, R., Boone, C., Brown, G.W., and Hieter, P. (2004). Identification of protein complexes required for efficient sister chromatid cohesion. Mol. Biol. Cell *15*, 1736–1745.

Mohanty, B.K., Bairwa, N.K., and Bastia, D. (2006). The Tof1p-Csm3p protein complex counteracts the Rrm3p helicase to control replication termination of Saccharomyces cerevisiae. Proc. Natl. Acad. Sci. USA *103*, 897–902.

Moreno, S.P., Bailey, R., Campion, N., Herron, S., and Gambus, A. (2014). Polyubiquitylation drives replisome disassembly at the termination of DNA replication. Science *346*, 477–481.

On, K.F., Beuron, F., Frith, D., Snijders, A.P., Morris, E.P., and Diffley, J.F. (2014). Prereplicative complexes assembled in vitro support origin-dependent and independent DNA replication. EMBO J. *33*, 605–620.

Osmundson, J.S., Kumar, J., Yeung, R., and Smith, D.J. (2017). Pif1-family helicases cooperatively suppress widespread replication-fork arrest at tRNA genes. Nat. Struct. Mol. Biol. *24*, 162–170.

Paeschke, K., Capra, J.A., and Zakian, V.A. (2011). DNA replication through G-quadruplex motifs is promoted by the Saccharomyces cerevisiae Pif1 DNA helicase. Cell *145*, 678–691.

Paeschke, K., Bochman, M.L., Garcia, P.D., Cejka, P., Friedman, K.L., Kowalczykowski, S.C., and Zakian, V.A. (2013). Pif1 family helicases suppress genome instability at G-quadruplex motifs. Nature *497*, 458–462.

Räschle, M., Knipscheer, P., Enoiu, M., Angelov, T., Sun, J., Griffith, J.D., Ellenberger, T.E., Schärer, O.D., and Walter, J.C. (2008). Mechanism of replication-coupled DNA interstrand crosslink repair. Cell *134*, 969–980.

Remus, D., Beuron, F., Tolun, G., Griffith, J.D., Morris, E.P., and Diffley, J.F. (2009). Concerted loading of Mcm2-7 double hexamers around DNA during DNA replication origin licensing. Cell *139*, 719–730.

Richter, A., Strausfeld, U., and Knippers, R. (1987). Effects of VM26 (teniposide), a specific inhibitor of type II DNA topoisomerase, on SV40 DNA replication in vivo. Nucleic Acids Res. *15*, 3455–3468.

Rossi, M.L., Pike, J.E., Wang, W., Burgers, P.M., Campbell, J.L., and Bambara, R.A. (2008). Pif1 helicase directs eukaryotic Okazaki fragments toward the two-nuclease cleavage pathway for primer removal. J. Biol. Chem. *283*, 27483–27493.

Ruone, S., Rhoades, A.R., and Formosa, T. (2003). Multiple Nhp6 molecules are required to recruit Spt16-Pob3 to form yFACT complexes and to reorganize nucleosomes. J. Biol. Chem. *278*, 45288–45295.

Sabouri, N., McDonald, K.R., Webb, C.J., Cristea, I.M., and Zakian, V.A. (2012). DNA replication through hard-to-replicate sites, including both highly transcribed RNA Pol II and Pol III genes, requires the S. pombe Pfh1 helicase. Genes Dev. *26*, 581–593.

Samora, C.P., Saksouk, J., Goswami, P., Wade, B.O., Singleton, M.R., Bates, P.A., Lengronne, A., Costa, A., and Uhlmann, F. (2016). Ctf4 Links DNA Replication with Sister Chromatid Cohesion Establishment by Recruiting the Chl1 Helicase to the Replisome. Mol. Cell *63*, 371–384.

Schalbetter, S.A., Mansoubi, S., Chambers, A.L., Downs, J.A., and Baxter, J. (2015). Fork rotation and DNA precatenation are restricted during DNA replication to prevent chromosomal instability. Proc. Natl. Acad. Sci. USA *112*, E4565–E4570.

Seidman, M.M., and Salzman, N.P. (1979). Late replicative intermediates are accumulated during simian virus 40 DNA replication in vivo and in vitro. J. Virol. *30*, 600–609.

Singleton, M.R., Dillingham, M.S., and Wigley, D.B. (2007). Structure and mechanism of helicases and nucleic acid translocases. Annu. Rev. Biochem. *76*, 23–50.

Skibbens, R.V. (2004). Chl1p, a DNA helicase-like protein in budding yeast, functions in sister-chromatid cohesion. Genetics *166*, 33–42.

Snapka, R.M., Powelson, M.A., and Strayer, J.M. (1988). Swiveling and decatenation of replicating simian virus 40 genomes in vivo. Mol. Cell. Biol. *8*, 515–521.

Snow, B.E., Mateyak, M., Paderova, J., Wakeham, A., Iorio, C., Zakian, V., Squire, J., and Harrington, L. (2007). Murine Pif1 interacts with telomerase and is dispensable for telomere function in vivo. Mol. Cell. Biol. *27*, 1017–1026.

Sonneville, R., Moreno, S.P., Knebel, A., Johnson, C., Hastie, C.J., Gartner, A., Gambus, A., and Labib, K. (2017). CUL-2^{LRR-1} and UBXN-3 drive replisome disassembly during DNA replication termination and mitosis. Nat. Cell Biol. *19*, 468–479.

Steinacher, R., Osman, F., Dalgaard, J.Z., Lorenz, A., and Whitby, M.C. (2012). The DNA helicase Pfh1 promotes fork merging at replication termination sites to ensure genome stability. Genes Dev. *26*, 594–602.

Stodola, J.L., and Burgers, P.M. (2017). Mechanism of Lagging-Strand DNA Replication in Eukaryotes. Adv. Exp. Med. Biol. *1042*, 117–133.

Sundin, O., and Varshavsky, A. (1980). Terminal stages of SV40 DNA replication proceed via multiply intertwined catenated dimers. Cell *21*, 103–114.

Suski, C., and Marians, K.J. (2008). Resolution of converging replication forks by RecQ and topoisomerase III. Mol. Cell *30*, 779–789.

Szyjka, S.J., Viggiani, C.J., and Aparicio, O.M. (2005). Mrc1 is required for normal progression of replication forks throughout chromatin in S. cerevisiae. Mol. Cell *19*, 691–697.

Taylor, M.R.G., and Yeeles, J.T.P. (2018). The Initial Response of a Eukaryotic Replisome to DNA Damage. Mol. Cell *70*, 1067–1080.e12.

Tourrière, H., Versini, G., Cordón-Preciado, V., Alabert, C., and Pasero, P. (2005). Mrc1 and Tof1 promote replication fork progression and recovery independently of Rad53. Mol. Cell *19*, 699–706.

Tran, P.L.T., Pohl, T.J., Chen, C.F., Chan, A., Pott, S., and Zakian, V.A. (2017). PIF1 family DNA helicases suppress R-loop mediated genome instability at tRNA genes. Nat. Commun. *8*, 15025.

Vafabakhsh, R., and Ha, T. (2012). Extreme bendability of DNA less than 100 base pairs long revealed by single-molecule cyclization. Science *337*, 1097–1101.

Vos, S.M., Tretter, E.M., Schmidt, B.H., and Berger, J.M. (2011). All tangled up: how cells direct, manage and exploit topoisomerase function. Nat. Rev. Mol. Cell Biol. *12*, 827–841.

Yeeles, J.T., Deegan, T.D., Janska, A., Early, A., and Diffley, J.F. (2015). Regulated eukaryotic DNA replication origin firing with purified proteins. Nature *519*, 431–435.

Yeeles, J.T.P., Janska, A., Early, A., and Diffley, J.F.X. (2017). How the Eukaryotic Replisome Achieves Rapid and Efficient DNA Replication. Mol. Cell *65*, 105–116.

STAR★METHODS

KEY RESOURCES TABLE

REAGENT or RESOURCE	SOURCE	IDENTIFIER
Antibodies		
Anti-fluorescein-AP Fab fragments	Roche	000000011426338910
Bacterial and Virus Strains		
Escherichia coli: Rosetta (DE3) pLysS cells: F^- *ompT hsdS*$_B$(r_B^- m_B^-) *gal dcm* (DE3) pLysSRARE (Cam^R)	Novagen	70956
Chemicals, Peptides, and Recombinant Proteins		
Anti-FLAG M2 affinity gel	Sigma-Aldrich	A2220
Glutathione Sepharose 4B	GE Healthcare	17075601
Ni-NTA agarose	QIAGEN	30210
Calmodulin Sepharose 4B	GE Healthcare	17052901
Streptactin superflow resin	IBA Life Sciences	2-1206-002
IgG Sepharose 6 Fast Flow	GE Healthcare	17096901
3Flag peptide	Sigma-Aldrich	F4799
Roche Complete EDTA-free protease inhibitor cocktail	Roche	000000011873580001
Sigma protease inhibitor cocktail	Sigma-Aldrich	P8215
Critical Commercial Assays		
CDP-Star	GE Healthcare	GERPN3682
Recombinant proteins are detailed in Table S4		
Experimental Models: Cell Lines		
Sf21 insect cells	ThermoFisher Scientific	11497013
Experimental Models: Organisms/Strains		
S. cerevisiae strains are detailed in Table S5		
Recombinant DNA		
pAM3 (Cdc6 purification)	Frigola et al., 2013	N/A
pJY19 (PCNA purification)	Yeeles et al., 2017	N/A
pRJ1228-Nhp6 (Nhp6 purification)	Ruone et al., 2003	N/A
pCDFduet.H2A-H2B (histones purification)	Kingston et al., 2011	N/A
pCDFduet.H3-H4(histones purification)	Kingston et al., 2011	N/A
pJFDJ5 (GINS purification)	Yeeles et al., 2015	N/A
pET28a-Mcm10 (Mcm10 purification)	Yeeles et al., 2015	N/A
pTF175 (FACT purification)	Biswas et al., 2005	N/A
pJW22 (FACT purification)	Biswas et al., 2005	N/A
pCFK1 (Nap1 purification)	Kurat et al., 2017	N/A
pET11d-Srs2 (Srs2 purification)	Marini and Krejci, 2012	N/A
pTDK10 (Pif1 purification)	This study	N/A
pTDK24 (Pif1 K264A purification)	This study	N/A
pTDK4 (generation of yTDK4 for Csm3-Tof1 purification)	This study	N/A
pTDK15 (generation of yTDK9 for Rrm3 purification)	This study	N/A
pTDK8 (generation of yTDK6 for Top1 purification)	This study	N/A
pTDK34 (generation of yTDK17 for Rrm3 K260A purification)	This study	N/A
pFV36 (generation of yFV43 for Dna2 purification)	This study	N/A
pTDK18 (generation of yTDK18 for Fen1 purification)	This study	N/A
pTDK19 (generation of yTDK19 for Cdc9 purification)	This study	N/A
pTDK13 (template for generation of molecular weight markers)	This study	N/A

(*Continued on next page*)

Continued		
REAGENT or RESOURCE	SOURCE	IDENTIFIER
pBS/ARS1WTA (3.2 kb replication template)	Marahrens and Stillman, 1992	N/A
pCFK1_WT (5.8 kb replication template)	Yeeles et al., 2015	N/A
pVA18 (5.5 kb replication template)	Valentina Aria	N/A
pZN3 (9.7 kb replication template)	Taylor and Yeeles, 2018	N/A
pRS425	Baxter laboratory	N/A
λ DNA-HindIII Digest: molecular weight markers	New England Biolabs	N3012S
M13 ssDNA	New England Biolabs	N4040S
Software and Algorithms		
ImageJ	National Institute of Health	https://imagej.nih.gov/ij/

CONTACT FOR REAGENT AND RESOURCE SHARING

Further information and requests for resources and reagents should be directed to the Lead Contact, Karim Labib (kpmlabib@dundee.ac.uk).

EXPERIMENTAL MODEL AND SUBJECT DETAILS

The *Saccharomyces cerevisiae* strain yJF1 (*MAT***a** *ade2-1 ura3-1 his3-11,15 trp1-1 leu2-3,112 can1-100 bar1Δ::hphNT pep4Δ::kanMX*) was transformed with linearized plasmids using standard genetic procedures to generate the protein expression strains, as detailed in the 'Key Resources Table', Table S1 and Table S5. The codon usage of the synthetic gene constructs that were used for the protein expression strains was optimized for high-level expression in *Saccharomyces cerevisiae* (Yeeles et al., 2015).

For expression of proteins in *E. coli*, the corresponding plasmids (listed in the Key Resources Table and Table S1) were transformed into Rosetta (DE3) pLysS cells (Novagen) (F^- *ompT hsdS*$_B$(r_B^- m_B^-) *gal dcm* (DE3) pLysSRARE (Cam^R).

METHOD DETAILS

Yeast methods

For expression of proteins, the corresponding strains (see Table S5) were grown at 30°C in YP + 2% raffinose to a density of 2–4 × 10^7 cells / ml. For expression of Mrc1, Csm3-Tof1, Rrm3, Top1, Fen1 and Cdc9, the cells were arrested in G1-phase by incubation for 3 h with 200 ng / ml alpha factor mating pheromone (Pepceuticals). Protein expression was then induced by addition of galactose to 2% for 3 h at 30°C. RFC, Pol δ and Dna2 expression was induced in the same way, but in asynchronous cultures.

Following expression, cells were collected by centrifugation and washed once with lysis buffer (see purification protocols for details of buffers used) without protease inhibitors. The cell pellets were then resuspended in 0.3–0.4 volumes of lysis buffer + protease inhibitors (see purification protocols for details) and the resulting suspensions were frozen drop-wise in liquid nitrogen. The frozen cells were crushed in a freezer mill (SPEX CertiPrep 6850 Freezer/Mill) with 4 cycles of 2′ at a rate of 15. The resulting powders were stored at −80°C until required.

For the experiments in Figure 7 and Figure S7, cells containing the plasmid pRS425 were grown to early log phase at 30°C, in minimal media supplemented with 2% glucose but lacking leucine, before transfer to rich medium supplemented with 2% Glucose (YPD: 1% yeast extract, 2% peptone), supplemented with 40 μg / ml of adenine. The cells were then grown to mid-log phase and arrested in G1-phase by addition of 10 μg / ml alpha factor per hour, until 95% of cells were unbudded (typically 120'). Cells were then washed three times in YPD medium lacking alpha factor, before further incubation in YPD. 'Time 0' was designated as the time of the addition of the first wash to the pelleted cells. Samples were taken at the indicated time points and used to prepare genomic DNA ($2x10^8$ cells were pelleted and frozen on dry ice) or to monitor DNA content by flow cytometry (Labib et al., 1999).

Protein purification buffers

Buffer A: 25 mM HEPES KOH pH 7.6, 10% glycerol, 0.02% NP-40-S, 1 mM DTT
Buffer B: 25 mM Tris-Cl pH 7.2, 10% glycerol, 1 mM DTT
Buffer C: 25 mM Tris-Cl pH 8.5, 10% glycerol, 0.02% NP-40-S, 1mM DTT
Buffer D: 25 mM Tris-Cl pH 7.2, 10% glycerol, 0.02% NP-40-S, 1 mM DTT
Buffer E: 25 mM HEPES KOH pH 7.6, 10% glycerol, 5 mM MgOAc, 1 mM DTT
Buffer F: 50 mM HEPES KOH pH 7.6, 10% glycerol, 1 mM DTT, 0.02% NP-40

Homemade protease inhibitor cocktail

1 mM PMSF, 5 mM benzamidine HCl, 1 mM AEBSF, 1 μg/ml pepstatin A, 1 μg/ml aprotinin

One protease inhibitor tablet (Roche, 000000011873580001) was used per 25 mL of lysis buffer where indicated.

1 mL of Sigma protease inhibitor cocktail (Sigma-Aldrich, P8215) was used per 100 mL of lysis buffer where indicated.

Protein purification

Yeast protein expression strains, and expression plasmids for protein purification from *Escherichia coli* are detailed in the Key Resources Table, Table S1 and Table S5. Proteins purified in this study are listed in Table S4. During extract preparation, thawed yeast cell powder was resuspended in 2-3 volumes of the initial lysis buffer in all cases. After affinity purification from yeast or bacterial cell lysates, the affinity resin was washed with at least 40 column volumes of wash buffer in all instances.

Cdc9

Powder was thawed in buffer C / 0.2 M NaCl / protease inhibitors (Roche tablets + Sigma protease inhibitors + homemade cocktail). Insoluble material was removed by centrifugation (235,000 x g, 4°C, 1 h) and the supernatant was mixed with 2.5 mL anti-FLAG M2 affinity gel (Sigma-Aldrich) at 4°C for 90 min.

The resin was collected and washed with buffer C / 0.2 M NaCl. Cdc9 was eluted in 1 column volume of buffer C / 0.2 M NaCl / 0.5 mg / ml 3FLAG peptide, then 1 column volume of buffer C / 0.2 M NaCl / 0.25 mg / ml 3FLAG peptide.

The eluate fraction was diluted 2-fold in buffer C then loaded onto a 1 mL HiTrap Q column in buffer C / 0.1 M NaCl. Cdc9 was eluted with a 20 column-volume gradient from 0.1 – 0.7 M NaCl in buffer C. Cdc9 containing fractions were pooled, dialysed versus buffer A / 0.2 M KOAc, snap frozen and stored at −80.

Chl1

A 20 mL aliquot of P5 viral supernatant, expressing full-length Chl1 including an N-terminal 2HA-6His-2Strep tag (a kind gift from Martin Singleton) was added to 2.8 l SF21 insect cells at a cell density of 1 million cells / ml. The cells were grown at 27°C with shaking at 110 rpm for 48 h. The cells were then harvested by centrifugation at 2500 rpm for 15 min at 4°C in a JLA9.1000 rotor (Beckman). The cell pellets were washed once in PBS + 5 mM MgOAc, resuspended in 6 volumes 25 mM HEPES pH 7.6, 0.02% Tween-20, 10% glycerol, 1 mM EDTA, 1 mM EGTA, 15 mM KCl, 2 mM MgOAc, 0.4 mM PMSF, 2 mM 2-mercaptoethanol, Roche protease inhibitor tablets, then snap frozen in aliquots.

Subsequently, the frozen cell suspension was thawed at room temperature, left on ice for 10 min, then lysed in a dounce homogenizer (20x compressions). KCl was added to 0.3 M and the sample centrifuged at 40,000 x g for 30 min at 4°C in a JA30.5 rotor (Beckman). The soluble fraction was recovered and mixed with 3 mL Streptactin superflow resin (IBA Life Sciences) for 90 min at 4°C with rotation. The beads were collected in a disposable column and washed extensively in 50 mM Tris-HCl pH 8.5, 0.3 M NaCl, 10% Glycerol, 1 mM DTT, then Chl1 was eluted in 2 column-volumes of 50 mM Tris-HCl pH 8.5, 0.3 M NaCl, 10% Glycerol, 1 mM DTT, 2.5 mM desthiobiotin.

The eluate fractions were pooled and the tag removed by overnight cleavage with 100 μg TEV protease at 4°C. The sample was diluted 3-fold in 20 mM Tris-HCl pH 8.5, 10% Glycerol, 1 mM DTT and then loaded onto a 1 mL HiTrap Q column. Chl1 was eluted with a 20 column-volume gradient from 0.1 – 0.4 M NaCl in 20 mM Tris-HCl pH 8.5, 0.1 M NaCl, 10% Glycerol, 1 mM DTT. The peak fractions were pooled, diluted with 20 mM Tris-HCl pH 8.5, 10% Glycerol, 1 mM DTT to a conductivity equivalent of 0.1 M NaCl, and loaded onto a 0.24 mL MiniQ column. Chl1 was eluted with a 5 mL gradient from 0.1 – 0.4 M NaCl. The peak fractions containing Chl1 were pooled, aliquoted and snap frozen.

Csm3-Tof1

Powder was thawed in buffer A / 0.2 M NaCl / protease inhibitors (Roche tablets + homemade cocktail) and the insoluble material removed by centrifugation (235,000 x g, 4°C, 1 h). The soluble extract was supplemented with 2 mM $CaCl_2$, 2 mL calmodulin affinity resin was added, and the mixture incubated with rotation at 4°C for 90 min.

The resin was collected and washed extensively with buffer A / 0.2 M NaCl. The washed resin was resuspended in one column volume of buffer A / 0.2 M NaCl and incubated with 200 μg TEV protease for 4 h at 4°C. The flow-through was collected and HIS-tagged TEV protease was depleted with 1 mL Ni-NTA beads (QIAGEN, 30210).

The Csm3-Tof1 sample was then separated on a 24 mL Superose 6 column in buffer A / 0.2 M NaCl. Csm3-Tof1 peak fractions were pooled, dialysed against buffer A / 0.3 M KOAc, snap frozen and stored at −80°C.

Dna2

Powder was thawed in buffer A / 0.2 M KCl / protease inhibitors (Roche tablets + homemade cocktail) and insoluble material removed by centrifugation (235,000 x g, 4°C, 1 h). The soluble extract was supplemented with 2 mM $CaCl_2$, 2 mL calmodulin affinity resin was added, and the mixture incubated with rotation at 4°C for 1 h.

The resin was collected and washed extensively with buffer A / 0.2 M KCl. The washed resin was then resuspended in 1 column-volume of buffer A / 0.2 M KCl and incubated with 240 μg TEV protease at room temperature for 1 h. The flow-through was collected and diluted to a conductivity equivalent of 0.1 M KCl with buffer A. The resulting sample was loaded onto 1 mL HiTrap Q column equilibrated in buffer A / 0.1 M KCl. Dna2 was then eluted with a 20 column-volume gradient from 0.1 – 0.7 M KCl in buffer A.

Dna2 containing fractions were pooled, concentrated and separated on a 24 mL Superdex 200 column in buffer A / 0.3 M KOAc without NP-40. Dna2 containing fractions were then pooled, concentrated, aliquoted and snap frozen.

Fen1

Powder was thawed in buffer C / 0.2 M NaCl / protease inhibitors (Roche tablets + Sigma protease inhibitors + homemade cocktail). Insoluble material was removed by centrifugation (235,000 g, 4°C, 1 h) and the supernatant was mixed with 2.5 mL anti-FLAG M2 affinity gel (Sigma-Aldrich) at 4°C for 90 min.

The resin was collected and washed with buffer C / 0.2 M NaCl. Fen1 was eluted in 1 column volume of buffer C / 0.2 M NaCl / 0.5 mg / ml 3FLAG peptide, then 1 column volume of buffer C / 0.2 M NaCl / 0.25 mg / ml 3FLAG peptide.

The eluate fraction was diluted 2-fold in buffer C then loaded onto a 1ml HiTrap heparin HP column in buffer D / 0.1 M NaCl. Fen1 was eluted with a 20 column-volume gradient from 0.1 – 1 M NaCl in buffer D. Fen1 containing fractions were pooled, dialysed versus buffer D / 0.3 M KOAc, snap frozen and stored at −80.

Mrc1

Powder was thawed in buffer A / 0.5 M NaCl / protease inhibitors (Roche tablets + homemade cocktail) and insoluble material was removed by centrifugation (235,000 x g, 4°C, 1 h). The soluble extract was mixed with 4 mL anti-FLAG M2 affinity gel (Sigma-Aldrich, A2220) and the mixture incubated with rotation at 4°C for 90 min.

The resin was collected and washed extensively with 120 mL buffer A / 0.5 M NaCl / protease inhibitors, then 20 mL buffer A / 0.5 M NaCl, then 40 mL buffer A / 0.5 M NaCl / 10 mM MgOAc / 1 mM ATP, then 20 mL buffer A / 0.2 M NaCl. Mrc1–5FLAG was eluted in 1 column volume of buffer A / 0.2 M NaCl / 0.5 mg / ml 3FLAG peptide, followed by 2 column volumes of buffer A / 0.2 M NaCl / 0.25 mg / ml 3FLAG peptide.

The eluate was diluted to a conductivity equivalent to 0.1 M NaCl with buffer A. The resulting sample was loaded onto 1 mL HiTrap Q column equilibrated in buffer A / 0.1 M NaCl. Mrc1 was eluted with a 20 column-volume gradient from 0.1 – 1 M NaCl in buffer A.

Mrc1-containing fractions were pooled and dialysed against buffer A / 40% glycerol / 0.3 M KOAc for 4 h at 4°C. The dialysed sample was recovered, aliquoted and snap frozen.

Pif1 (S. cerevisiae)

Rosetta *E. coli* cells (Novagen) were transformed with the relevant Pif1 expression vector (see Key Resources Table). The transformant colonies were inoculated into a 250 mL LB / ampicillin (50 μg/ml) / chloramphenicol (35 μg/ml) culture, which was grown overnight at 37°C with shaking at 200 rpm. The following morning, the culture was diluted into 1-2 l of LB / ampicillin (50 μg/ml) / chloramphenicol (35 μg/ml) to a final OD_{600} of 0.15. The culture was left to grow at 37°C until an OD_{600} of 0.6 was reached. Cells were cooled on ice for 30 min, 1 mM IPTG was added to induce expression, and then the cells were incubated overnight at 23°C. The cells were harvested by centrifugation at 5000 rpm for 10 min in a JLA-9.1000 rotor (Beckman).

For lysis, cell pellets were resuspended in 30 mL of buffer E / 0.3 M NaCl / 30 mM imidazole / Roche protease inhibitor tablets. Lysozyme was added to a final concentration of 500 μg/ml and the mixture then left for 20 min on ice. Subsequently, the sample was sonicated for 90 s (15 s on, 30 s off) at 40% on a Branson Digital Sonifier. Insoluble material was removed by centrifugation at 15000 rpm for 30 min in an SS-34 rotor (Sorvall).

The supernatant was subjected to Ni^{2+} affinity purification by incubation with 1 mL packed bead volume of Ni-NTA resin (QIAGEN) for 90 min at 4°C. The beads were recovered in a disposable gravity flow column and washed extensively with buffer E / 0.3 M NaCl / 30 mM imidazole / Roche protease inhibitor tablets. Pif1 was eluted with 5 column volumes of buffer E / 0.3 M NaCl / 0.4 M imidazole.

The eluate was diluted 2-fold in buffer E lacking salt and then loaded onto a 1 mL HiTrap SP FF column pre-equilibrated in buffer E / 0.15 M NaCl. Protein was eluted with a 20 column-volume gradient from 0.15 – 1 M NaCl. Pif1 containing fractions were pooled and diluted to a conductivity equivalent of 0.15 M NaCl with buffer E lacking salt. The resultant fraction was then loaded onto a 1 mL HiTrap Heparin HP column pre-equilibrated in buffer E / 0.15 M NaCl. Protein was eluted with a 20 column-volume gradient from 0.15 - 1 M NaCl. Pif1 containing fractions were pooled and dialysed against 25 mM HEPES / 40% glycerol / 5 mM MgOAc / 0.3 M KOAc / 1 mM DTT for 4 h at 4°C. The dialysed sample was recovered, aliquoted and snap frozen.

BacPif1

Pif1 from Bacteroides sp 2 1 16 (BacPif1) was purified as for budding yeast Pif1, until the Ni^{2+} affinity purification step. Following elution from Ni-NTA resin, the eluate was diluted 2-fold in buffer E lacking salt and then loaded onto a 1 mL HiTrap Q HP column pre-equilibrated in buffer E / 0.15 M NaCl. Protein was eluted with a 20 column-volume gradient from 0.15 – 1 M NaCl. BacPif1 containing peak fractions were pooled and dialysed against 25 mM HEPES / 40% glycerol / 5 mM MgOAc / 0.3 M KOAc / 1 mM DTT at 4°C for 4 h. The dialysed sample was recovered, aliquoted and snap frozen in liquid nitrogen.

Pol α / primase

Buffer A / 0.4 M NaCl / protease inhibitors (Roche tablets + homemade cocktail) was added to cell powder and the resuspension was centrifuged (235,000 x g, 4°C, 1 h). The soluble extract was supplemented with 2 mM $CaCl_2$ and Pol α / primase was purified by calmodulin affinity chromatography using 2 mL calmodulin affinity resin (GE Healthcare, 17052901).

The eluate fractions were pooled and separated on a 24 mL Superdex 200 column in buffer B + 0.4 M KOAc. Pol α / primase peak fractions were pooled, concentrated, aliquoted and snap frozen.

Pol δ

Buffer A / 0.2 M NaCl / protease inhibitors (Roche tablets + homemade cocktail) was added to thawed powder and the sample was centrifuged (235,000 x g, 4°C, 1 h). The soluble extract was recovered and supplemented with 2 mM $CaCl_2$. At this point, 1.5 mL calmodulin affinity resin was added and the mixture incubated at 4°C for 90 min with rotation.

The resin was collected and washed extensively with buffer A / 0.2 M NaCl / 2 mM $CaCl_2$, and Pol δ was then eluted with buffer A / 0.2 M NaCl / 2 mM EDTA / 2 mM EGTA.

The eluate fractions were pooled, concentrated and loaded onto a 24 mL Superdex 200 column in buffer A / 0.3 M KOAc without NP-40. The peak fractions containing Pol δ were pooled, concentrated, aliquoted and snap frozen.

RFC

Powder was thawed in buffer A / 0.15 M NaCl / protease inhibitors (Roche tablets + homemade cocktail) and then centrifuged (235,000 x g, 4°C, 1 h). The soluble extract was recovered and supplemented with 2 mM $CaCl_2$ and RFC purified by camodulin affinity chromatography using 1 mL resin.

Eluate fractions were pooled and loaded onto a 1 mL HiTrap SP FF column in buffer A / 0.15 M NaCl. RFC was eluted with a 20 column-volume gradient from 0.15 – 1 M NaCl in buffer A. RFC containing fractions were pooled and dialysed against buffer A / 0.3 M KOAc for 4h at 4°C. The dialysed sample was recovered, concentrated, aliquoted and snap frozen.

Rrm3

The frozen cell powder was resuspended in 3 volumes of buffer F / 0.5 M KCl / protease inhibitors (Roche tablets + Sigma inhibitors + homemade cocktail) and centrifuged (235,000 x g, 4°C, 1 h). Solid ammonium sulfate was added gradually to the soluble extract to 30% final concentration with stirring (10 min, 4°C). The insoluble material was then removed by centrifugation (27,000 x g, 4°C, 20 min) and the supernatant was mixed with 4 mL anti-FLAG M2 affinity gel (Sigma-Aldrich) at 4°C for 30 min.

The resin was collected and washed with 40 column volumes of buffer A / 0.5 M KCl / protease inhibitors, then 10 column volumes of buffer A / 0.5 M KCl / 5 mM MgOAc / 1 mM ATP, then 10 column volumes of buffer A / 0.5 M KCl. At this point, 3FLAG-Rrm3 was eluted in 1 column volume of buffer A / 0.5 M KCl / 0.5 mg / ml 3FLAG peptide, then 2 column volumes of buffer A / 0.5 M KCl / 0.25 mg / ml 3FLAG peptide.

The eluate was dialysed against buffer A / 0.3 M KCl for 3 h at 4°C, then loaded onto a 1 mL HiTrap heparin column equilibrated in buffer A / 0.3 M KCl / 10 mM MgOAc / 1 mM ATP. Rrm3 was eluted with a 15 column-volume gradient from 0.3 – 1 M KCl in buffer A / 10 mM MgOAc / 1 mM ATP.

Peak fractions containing Rrm3 were pooled and dialysed against buffer A / 40% glycerol / 0.35 M KCl at 4°C for 4 h. The dialysed sample was recovered, aliquoted and snap frozen.

Srs2

Rosetta *E. coli* cells were transformed with pET11d-Srs2 (see Key Resources Table). A 200 mL LB / ampicillin (50 μg/ml) / chloramphenicol (35 μg/ml) culture was inoculated with transformant colonies and grown overnight at 37°C with shaking at 200 rpm. The following morning, the culture was diluted 20-fold into 2 l of LB / ampicillin (50 μg/ml) / chloramphenicol (35 μg/ml) and then left to grow at 37°C until an OD_{600} of 1 was reached. 0.1 mM IPTG was added and Srs2 expression induced overnight at 16°C. Cells were harvested by centrifugation for 10 min in a JLA-9.1000 rotor (Beckman) at 5000 rpm.

For lysis, cell pellets were resuspended in 30 mL of buffer A / 0.6 M KCl / 30 mM imidazole / Roche protease inhibitor tablets. Sonication was performed as described for Pif1. Insoluble material was removed by centrifugation at 15,000 rpm for 30 min in an SS-34 rotor (Sorvall).

The supernatant was subjected to Ni^{2+} affinity purification by incubation with 1 mL packed bead volume of Ni-NTA resin (QIAGEN) for 90 min at 4°C. The beads were recovered in a disposable gravity flow column and washed extensively with buffer A / 0.6 M KCl / 30 mM imidazole / Roche protease inhibitor tablets. Srs2 was eluted with 10 column volumes of buffer A / 0.6 M KCl / 0.4 M imidazole.

The eluate was diluted 4-fold in buffer A lacking salt and then loaded onto a 1 mL HiTrap SP FF column pre-equilibrated in buffer A / 0.15 M KCl. Protein was eluted with a 20 column-volume gradient from 0.15 – 1 M KCl. Srs2 containing fractions were then pooled and loaded onto a 120 mL Superdex 200 column in buffer A / 0.2 M KCl. Peak fractions were then re-loaded onto a 1 mL HiTrap SP FF and Srs2 was eluted with a 10 column-volume gradient from 0.2 – 1 M KCl. The Srs2 containing fractions were pooled, concentrated and snap frozen.

Top1

Powder was thawed in buffer A / 0.3 M NaCl / protease inhibitors (Roche tablets + homemade cocktail) and insoluble material removed by centrifugation (235,000 x g, 4°C, 1 h). At this point, 2 mM $CaCl_2$ was added to the soluble extract, followed by 2 mL calmodulin affinity resin, and the mixture incubated for 90 min at 4°C.

The resin was collected and washed extensively with buffer A / 0.3 M NaCl. Top1 was eluted in 6 column volumes buffer A / 0.3 M NaCl / 2 mM EDTA / 2 mM EGTA. The Top1-containing fractions were pooled, concentrated and separated on a 24 mL Superdex 200 column in buffer A / 0.4 M KOAc. The peak fractions were pooled, concentrated, aliquoted and snap frozen.

Other proteins

Sgs1 and Top3-Rmi1 were a kind gift from Dr. Stephen Kowalczykowski. *E. coli* TopoIV was purchased from Inspiralis (T4001). ORC, Cdc6, Cdt1-Mcm2-7, DDK, S-CDK, Sld3/7, Cdc45, Dpb11, Pol ε, Sld2, GINS, Mcm10, Ctf4, Top2, PCNA, RPA, ISWI, Nap1, Nhp6, FACT and histones were purified by adapting previously established protocols (Coster et al., 2014; Frigola et al., 2013; Kurat et al., 2017; On et al., 2014; Yeeles et al., 2015). A brief purification strategy for each of these proteins is listed in the Table S3.

DNA templates

The DNA templates pBS/ARS1WTA (3.2 kb), pCFK1_WT (5.8 kb), pZN3 (9.7 kb) and pRS425 have been described previously (Christianson et al., 1992; Marahrens and Stillman, 1992; Taylor and Yeeles, 2018; Yeeles et al., 2015). pVA18 (5.5 kb) is a smaller derivative of the origin-specific plasmid pZN3. The DNA sequence of pVA18 (5589 bp) is provided below:

```
CTGACGCGCCCTGTAGCGGCGCATTAAGCGCGGCGGGTGTGGTGGTTACGCGCAGCGTGACCGCTACACTTGCCAGCGCC
CTAGCGCCCGCTCCTTTCGCTTTCTTCCCTTCCTTTCTCGCCACGTTCGCCGTCCTTCAATGAAACATCGTTGGCCACTAATTT
GGCCAGTGCAAAGTAGAACAAATCGGCAGCCTCCCAAGAAAGCTCCTTCTTACCCTTTGCCTCAGTCAGTTCTTCAGCTTCTT
CCTTGATCTTGGCATCTAACAATGCAGAGTCGTTGAATAGTCTTCTAGTATAAGATTCCTCTGGAGCGTCCTGTAGCCTTTGTTT
TAGTAAAGATTCTAGCCCCACCAAACCATGCTTGAATTCACCAAAGCAAGACATGGTCTCCAAGTGGCAAAATCCAACGTTTTC
TTGTTCAACGATAAACTTTAAGGCATCCGAATCACAGTCAGTAGAGATTTGTAAAAGCTTTTGGCCATTGCCAGAAGTTTCACCC
TTGATCCAGATTTCATTCCTAGAACGAGAATAATAAACGCCACGACCCAATTCGATGGCCTTTGCTATAGATTTCTTCGAAGAAT
ACACCAACCCTAGACAACGCTCATATTGGTCCACAACTAGGGTGGTATATAAACCGTCAGGACGGTCTGTACGTACTTCACCA
AGCACTTCTTTGGTCAACATATCCTTGCTTAATTTCTTTATGGACACAATTTTATCTTGCGAGAATTTTTGTTTTACCATGAATTGA
TTGGAGAAAACACCGTTCTCTTCCACAACAACACGCTCCTTTGGTACATTCAATTGTTCAACCAAGTGTTCGGCTGTTTTAGCA
TCTTGGCTTGCAATGAACAGAGAAGAAACTCCGTTGTTCAAGAAGGCAATGATTTCATCATCGCTGAATTTACCACTTGGCAAG
GACAAAGCCACCAATGGAACTTCTTCCTCTTTGGAGAACTGGAGAATCTCTTCATTACTCAGGCTCGAGCCATCCAAAAGTAC
CTGACCAACAAGTGAAACGTATTCCTTCTTACTATTCCATGAGGCCAGATCATCAATTAACGGTAGAATCGGCAAAACCATTAT
TCAGAAAAAAAATTTTGTAAACTATTGTATTACTATTACACAGCGCAGTTGTGCTATGATATTAAAATGTATCCAGAACACACATC
GGAGGTGAATATAACGTTCCATATCTATTATATACACAGTATACTACTGTTCATAGTCATATCCTTTTCTTACCTTCTATATCGAA
TGACTGATAATGCAACGTGAGTCACTGTGCATGGGTTTAGCAATTATTAAACTAATTTACCGGAGTCACTATTAGAGTCAGTTCG
ACTGCCTAGAAGAACTGCTGGTTGTCAGGATTGTGATGGGGGCATTCTGCTGTATTATGACCCATCGTATCGCAATGCTCACAC
CACTGTTGTCTTCCTGCCGTGGTATCGACTGGTGCAGGGGGGTCGAAAATTGGTATACGGTACTACTGCACAACAAACTTTGG
AAACCAACTTCAATGATCATCATGACTGCAATAAAAGCACTGAGAAACACGAGTTGATAATACCCACCCCATCAAAACCACTAA
AGAAAAGGATATAAAGAAGACAAAGTAAAATGTATCAGCATTTACAACATTTGTCACGTTCTAAACCATTGCCGCTTACTCCAAA
CTCCAAATATAATGGGGAGGCTTGCGTCCAATTAGGGAAGACATATACAGTTATTCAGGATTACGAGCCTAGATTGACAGACGA
AATAAGAATCTCGCTGGGTGAAAAAGTTAAAATTCTGGCCACTCATACCGATGGATGGTGTCTGGTAGAAAAGTGTAATACACA
AAAGGGTTCTATTCACGTCAGTGTTGACGATAAAAGATACCTCAATGAAGATAGAGGCATTGTGCCTGGTGACTGTCTCCAAGA
ATACGACTGATGAAAATAATATTGACGTTCGCATTTAATCTATACCTATAATTCTGTACTTATATACTGTTCCTTAATTGAAGATTTC
AACATCGTTTTTGATGTAGGTCTTTTCACCTGGAGGTGCGGCTGGGCTACCGAAGACTAATTGAGCTTGTACGGTCCAAGACTC
AGGGATTTTGCTTGGCAAAGCAGCTTTTATGTAACCATTGTAGTGTTGTAGGTGACCACCCAGGCCCATTGCCTCCAAGGCAAC
CCACGAGTTGATTTGAGCGGCACCAGAGGTATGGTCCGCGAAACTAGGGAATGCAGCTGGTACGCTGGGAAGTCAGCCTTTA
GCTTTTCAGTTACCTTGTCGTCGGTGAAGAAGATTACAGAACCAAAGGCCTCATCCCTTGCTGAAGCAGGCCTCTTTTGACCG
GCAGGGCTTTCTATAGCCTTAGTCACTTCGTCCCAAACTTTTTTGTGAGTTTCACCAGTCAAGATAACAGCGCGATTTGGCTGG
GAGTTGAAAGCGGTGGGTGTGGCTCGAATGATGGTTTGGACGACGGATTGGATGTCGTTGATAGTAATTTCACCAGGTGCGGC
CGCTTTCAAAGCGTAAATAGTACGACGAGCAGTTAAAGTTTTCAAATAAGTTGCAACAGCAGACATGATATTGGATTGCCGGAA
TGGCGATATGTTGATCCCGGATACTTCAGTCTACGAAAAAAGTACAAATTATGTGTCAGTTCCTTCAGTATGGTGTCCTTATATA
CTGTAGTTTGGACAAGGTGCAAATGCCAAGACCCTAGCCCGAAAAGCTCGAGGCACCCCAGGATCTTCCCCTTTACGTAATT
TTCACGTAAAACGCCACAGTCCGATTTTTCTAGAATAATCATTAGTAAAAGCGGTATACTGGATTATTGTACGATAACAAGGTAG
AGCTTTATTACTAAGCTAAGACGTTCTTACATCAATAGTGCTGTTCGTTATTGACGTCAGGAGAAGGAGCGGGTCTGGTGAATA
GTGTAAGCAGTGTTTCTGAACTTTTTCTTCGTCTAAGTCCTTGTAATGTAAGGTAAGAATGCAAGCATCTTGTTTGTAACCCGGG
TGTACGTTGACGTTAGTAAGGGGTGTACGTTGACGTTAGTAAGTCACAAACCCAAGCTTAACTTCTTCGTGAGGAAGGAAAGTG
TTGTCTCCTACTTTTTTCAAATTTTCGAATTGTATTTATATTTATTTAGTACTTCTTGAGTTTACATATCCTTCGTAAAAATGCAACTT
TTGTCGAAAAACACTTCCAAAAAAAAATAATAATGAATTTATGAAGCATACTAACGAGCGAGCACATCGCTGACCTATCATTACT
TCATGAGATAAATTAAGATCTCCTCATATGCGAATTTCCTGTTCAGTGATAAACGTTGATTACGTTATTGATAAAAGTCTTTTCTTC
TGGCAAGGGGTACCCAGCTTTTGTTCCCTTTAGTGAGGGTTAATTGCGCGCTTGGCGTAATCATGGTCATCGATGTTTCCTGTG
TGAAATTGTTATCCGCTCACAATTCCACACAACATACGAGCCGGAAGCATAAAGTGTAAAGCCTGGGGTGCCTAATGAGTGAG
CTAACTCACATTAATTAAGTTGCGCTCACTGCCCGCTTTCCAGTCGGGAAACCTGTCGTGCCAGCTGCATTAATGAATCGGCCA
ACGCGCGGGGAGAGGCGGTTTGCGTATTGGGCGCTCTTCCGCTTCCTCGCTCACTGACTCGCTGCGCTCGGTCGTTCGGCT
GCGGCGAGCGGTATCAGCTCACTCAAAGGCGGTAATACGGTTATCCACAGAATCAGGGGATAACGCAGGAAAGAACATGTGA
GCAAAAGGCCAGCAAAAGGCCAGGAACCGTAAAAAGGCCGCGTTGCTGGCGTTTTTCCATAGGCTCCGCCCCCCTGACGAG
CATCACAAAAATCGACGCTCAAGTCAGAGGTGGCGAAACCCGACAGGACTATAAAGATACCAGGCGTTTCCCCCTGGAAGCT
CCCTCGTGCGCTCTCCTGTTCCGACCCTGCCGCTTACCGGATACCTGTCCGCCTTTCTCCCTTCGGGAAGCGTGGCGCTTTC
TCATAGCTCACGCTGTAGGTATCTCAGTTCGGTGTAGGTCGTTCGCTCCAAGCTGGGCTGTGTGCACGAACCCCCCGTTCAG
CCCGACCGCTGCGCCTTATCCGGTAACTATCGTCTTGAGTCCAACCCGGTAAGACACGACTTATCGCCACTGGCAGCAGCC
ACTGGTAACAGGATTAGCAGAGCGAGGTATGTAGGCGGTGCTACAGAGTTCTTGAAGTGGTGGCCTAACTACGGCTACACTA
GAAGGACAGTATTTGGTATCTGCGCTCTGCTGAAGCCAGTTACCTTCGGAAAAAGAGTTGGTAGCTCTTGATCCGGCAAACAA
ACCACCGCTGGTAGCGGTGGTTTTTTTGTTTGCAAGCAGCAGATTACGCGCAGAAAAAAAGGATCTCAAGAAGATCCTTTGATC
```

TTTTCTACGGGGTCTGACGCTCAGTGGAACGAAAACTCACGTTAAGGGATTTTGGTCATGAGATTATCAAAAAGGATCTTCACC
TAGATCCTTTTAAATTAAAAATGAAGTTTTAAATCAATCTAAAGTATATATGAGTAAACTTGGTCTGACAGTTACCAATGCTTAATC
AGTGAGGCACCTATCTCAGCGATCTGTCTATTTCGTTCATCCATAGTTGCCTGACTCCCCGTCGTGTAGATAACTACGATACGG
GAGGGCTTACCATCTGGCCCCAGTGCTGCAATGATACCGCGAGACCCACGCTCACCGGCTCCAGATTTATCAGCAATAAACC
AGCCAGCCGGAAGGGCCGAGCGCAGAAGTGGTCCTGCAACTTTATCCGCCTCCATCCAGTCTATTAATTGTTGCCGGGAAGC
TAGAGTAAGTAGTTCGCCAGTTAATAGTTTGCGCAACGTTGTTGCCATTGCTACAGGCATCGTGGTGTCACGCTCGTCGTTTGG
TATGGCTTCATTCAGCTCCGGTTCCCAACGATCAAGGCGAGTTACATGATCCCCCATGTTGTGCAAAAAAGCGGTTAGCTCCTT
CGGTCCTCCGATCGTTGTCAGAAGTAAGTTGGCCGCAGTGTTATCACTCATGGTTATGGCAGCACTGCATAATTCTCTTACTGT
CATGCCATCCGTAAGATGCTTTTCTGTGACTGGTGAGTACTCAACCAAGTCATTCTGAGAATAGTGTATGCGGCGACCGAGTTG
CTCTTGCCCGGCGTCAATACGGGATAATACCGCGCCACATAGCAGAACTTTAAAAGTGCTCATCATTGGAAAACGTTCTTCGG
GGCGAAAACTCTCAAGGATCTTACCGCTGTTGAGATCCAGTTCGATGTAACCCACTCGTGCACCCAACTGATCTTCAGCATCTT
TTACTTTCACCAGCGTTTCTGGGTGAGCAAAAACAGGAAGGCAAAATGCCGCAAAAAAGGGAATAAGGGCGACACGGAAATGT
TGAATACTCATACTCTTCCTTTTTCAATATTATTGAAGCATTTATCAGGGTTATTGTCTCATGAGCGGATACATATTTGAATGTATT
TAGAAAAATAAACAAATAGGGGTTCCGCGCACATTTCCCCGAAAAGTGCCAC

Covalently closed plasmids for *in vitro* replication reactions were purified using alkaline lysis followed by caesium chloride density gradient centrifugation.

For the preparation of linear DNA templates, 4 μg plasmid DNA was incubated with 2 μL restriction enzyme (New England Biolabs) in 1X CutSmart Buffer (New England Biolabs) in a 40 μL final reaction volume. After incubating for 4h at 37°C, the sample was treated with SDS / proteinase K and the DNA purified by phenol / chloroform extraction. The DNA was then precipitated from the aqueous phase with 0.2 M NaCl and 3 volumes 100% ethanol, then left at −20°C overnight. The following morning, the DNA pellet was harvested by centrifugation, washed with 70% ethanol, harvested again, air-dried, and then resuspended in 15-20 μL TE.

Molecular weight markers

Standard molecular weight markers were prepared by dephosphorylating 17 μg λ DNA-HindIII Digest (New England Biolabs N3012S) with 10 U Antarctic Phosphatase (New England Biolabs M0289S) in total volume of 40 μL for 1 h at 37°C. The phosphatase was then inactivated by incubation at 80°C for 10 min. Subsequently, 6.8 μg of dephosphorylated DNA was labeled with γ-[^{32}P]-ATP using 40 units of T4 Polynucleotide Kinase (New England Biolabs M0201S) for 1 h at 37°C, in a total reaction volume of 40 μl. Unincorporated γ-[^{32}P]-ATP was removed using Illustra MicroSpin G-50 columns (GE Healthcare) and 5 mM EDTA was added to the recovered sample.

For Figure 1D, end-labeled 3189 bp plasmid was prepared by digesting 4 μg plasmid DNA with 2 μL SmaI (Roche) in 1X CutSmart Buffer (New England Biolabs) in a 40 μL final reaction volume for 2 h at 25°C. The linearized plasmid was then column purified using the High Pure PCR Product Purification Kit (Roche) and then dephosphorylated and end-labeled with γ-[^{32}P]-ATP, as described for standard molecular weight markers. Other labeled markers in Figure 1D were generated by PCR amplification using oligonucleotides 7272 – 7275 (see Table S2) and pTDK13 plasmid template. In each case, 50 μL PCR reactions were assembled in the presence of 33 nM α-[32P]-dCTP and the PCR products were purified over Illustra MicroSpin G-50 columns (GE Healthcare).

In vitro replication assays

Mcm2-7 loading and DDK phosphorylation was performed as follows. First, 5 nM plasmid DNA template, 5-10 nM ORC, 20 nM Cdc6, 40 nM Cdt1/Mcm2-7 and 20 nM DDK were incubated in 25 mM HEPES-KOH (pH 7.6), 100 mM potassium acetate, 0.02% NP-40-S, 0.1 mg / ml BSA, 1 mM DTT, 10 mM $Mg(OAc)_2$, 5 mM ATP at 30°C for 10 min.

Subsequently, separate buffer and replication protein mixtures were added sequentially to the Mcm2-7 loading mixture to give a final replication reaction containing 25 mM HEPES-KOH (pH 7.6), 100 mM potassium acetate (unless otherwise indicated), 0.02% NP-40-S, 0.1 mg/ml BSA, 1 mM DTT, 10 mM $Mg(OAc)_2$, 2.75 mM ATP, 30 μM dATP-dCTP-dGTP-dTTP, 33 nM α-[32P]-dCTP, 400 μM CTP-GTP-UTP, 20 nM S-CDK, 30 nM Dpb11, 8 nM GINS, 40 nM Cdc45, 30 nM Pol ε, 5 nM Mcm10, 5 nM RFC, 20 nM PCNA, 20 nM Top1, 20 nM Top2, 20 nM Pol α−primase, 25 nM Sld3-7, 10 nM Ctf4, 100 nM RPA, 20 nM Csm3-Tof1, 20 nM Mrc1, 50 nM Sld2, 5 nM Pol δ, 10 nM Fen1 and 20 nM Cdc9 (unless otherwise indicated).

Pol δ, Fen1 and Cdc9 were generally omitted from replication reactions including accessory DNA helicases (except in Figures 6C and 6D and Figure S4A), in order to prevent strand displacement synthesis by Pol δ, which has previously been shown to be enhanced by Pif1-Rrm3 (Osmundson et al., 2017; Rossi et al., 2008). The final concentration of potassium acetate was adjusted to 250 mM in those reactions that included Pol δ.

2-5 μL of the Mcm2-7 loading mixture was generally used per sample and this was typically diluted 4-fold in the final reaction. The extra contribution from protein storage buffers to the final reaction was approximately 22 mM chloride and 50-60 mM acetate, and the corresponding potassium counter-ions. Replication reactions were conducted at 30°C for 20 min unless otherwise indicated.

Pif1 and Rrm3 were included at 5 nm and 12.5 nM, respectively, unless otherwise indicated. For Figure 4C, Sgs1 and Top3-Rmi1 were included at 10 nM. Srs2, Chl1 and Dna2 were included at 20 nM.

RFC, PCNA, Top2, Ctf4, Csm3-Tof1, Mrc1, Pol δ, Fen1 and Cdc9 were omitted from reactions with the ‘minimal replisome’, as in Figure S1G.

Origin-specific replication reactions were conducted as above, except that Mcm2-7 loading was conducted at 24°C for 10 min and 80 nM S-CDK was then added directly to this reaction at 24°C for 5 min before the replication step.

For pulse-chase experiments, dATP, dTTP, dGTP and dCTP were each added to 600 μM in the chase at the indicated times. For the experiments in Figures 1F, 3C, S4B, S5A, the dCTP concentration was adjusted to 2.5 μM in the pulse. For the experiments in Figures 3C and S4B, SmaI (Roche) was added to a final concentration of 0.5 U/μl concomitant with the chase.

Chromatin replication experiments were carried out using a modified version of an existing protocol (Taylor and Yeeles, 2018). Briefly, for chromatin assembly, ISW1 (30 nM), Nap1 (3 μM) and histone octamers (150 nM) were incubated in chromatin assembly buffer (25 mM HEPES-KOH (pH 7.6), 10 mM $Mg(OAc)_2$, 100 mM KOAc, 0.01% NP-40-S, 5% glycerol, 0.1 mg/ml BSA) for 10 min on ice. Creatine phosphate (40 mM), creatine phosphate kinase (100 μg/ml) and ATP (3 mM) were then added and chromatin assembly initiated by the addition of plasmid template (3 nM). Chromatin assembly was performed at 30°C for 1 h, with addition of ORC (10 nM) after 10 min incubation.

Chromatinised plasmids were purified by applying a 40 μL chromatin assembly reaction to a 400 μL Sephacryl S-400 column (prepared in a 0.8 mL Pierce Centrifuge Column (Thermo Scientific 89868)) pre-equilibrated in 25 mM HEPES-KOH (pH 7.6), 100 mM potassium acetate, 0.02% NP-40-S, 1 mM DTT, 10 mM $Mg(OAc)_2$, and then centrifuging at 700 x *g* for 2 min. Chromatin replication was performed as for naked DNA templates with the addition of FACT (80 nM) and Nhp6 (400 nM) in the replication step. Pol δ was included at 10 nM in chromatin replication experiments.

For Figure 4A, reactions were conducted as with the reconstituted system reactions, except that S-phase extract (8 mg / ml) (On et al., 2014) was used instead of purified proteins during the replication step.

Preparation of substrates for helicase assays

28 pmol of PAGE-purified oligonucleotides purchased from Sigma (see Table S2 for oligonucleotide sequences) were first labeled with γ-[^{32}P]-ATP using 20 U T4 Polynucleotide Kinase (New England Biolabs M0201S) for 1 h at 37°C in a total reaction volume of 20 μL. Unincorporated γ-[^{32}P]-ATP was then removed using illustra MicroSpin G-50 columns (GE Healthcare).

For annealing, a 3-fold molar excess of labeled oligonucleotide was incubated with 1.3 pmol of M13 ssDNA (New England Biolabs N4040S) in a total reaction volume of 20 μL with heating to 95°C for 10 min in a metal heating block. The metal block was then placed at room temperature and the sample left to cool for 3 h. Free oligonucleotide was removed from the sample by applying a 20 μL annealing reaction to a 400 μL Sephacryl S-400 column (prepared in 0.8 mL Pierce Centrifuge Columns (Thermo Scientific 89868)) pre-equilibrated in TE. The final concentration of the recovered substrate was estimated to be 38 nM. Substrates were stored at 4°C.

Helicase assays

Helicase assays (10 μL volume) were carried out using 1 nM M13-based substrate in buffer containing 25 mM HEPES-KOH (pH 7.6), 0.1 mg/ml BSA, 2 mM $Mg(OAc)_2$ and 2 mM ATP. Reactions were assembled on ice, equilibrated to room temperature and the respective helicases added to 50 nM final concentration. Reactions were incubated at 30°C for 30 min.

Reactions were stopped by the addition of EDTA (20 mM), SDS (0.4%) and proteinase K (1/25 volumes) and the incubation continued at 37°C for 10 min. The samples were supplemented with Novex Hi-Density TBE Sample Buffer (ThermoFisher Scientific LC6678) and analyzed in 4%–20% Novex TBE Gels (ThermoFisher Scientific EC62252BOX) at 200 V for 30 min in 1X TBE. Gels were mounted onto chromatography paper (GE Healthcare, 3030-861) and exposed to Amersham Hyperfilm ECL (GE Healthcare) at −80°C.

Catenation assay with Sgs1-Top3-Rmi1

The catenation assay in Figure S3A was carried out using a modified version of an existing protocol (Cejka et al., 2012). The 3.2 kb plasmid (5 nM) was incubated with Sgs1 (60 nM), Top3-Rmi1 (400 nM) and RPA (100 nM) at 30°C for 30 min in a 10 μL reaction containing 25 mM HEPES-KOH (pH 7.6), 0.1 mg/ml BSA, 1 mM $Mg(OAc)_2$, 1 mM ATP, 100 μg / ml creatine phosphate kinase and 40 mM creatine phosphate. The reaction was stopped by addition of EDTA (50 mM), SDS (0.1%) and proteinase K (1 / 100 volume) and the incubation continued at 37°C for 60 min. Reaction products were then separated in a 1% native agarose gel, which was subsequently stained with EtBr (0.5 μg / ml) for 20 min at room temperature.

DNA preparation for agarose gel electrophoresis

For all *in vitro* replication experiments, reactions were quenched by addition of EDTA to 25 mM. SDS (0.1%) and proteinase K (1 / 100 volumes) were subsequently added and the incubation continued at 37°C for 30 min. To this was added an equal volume of phenol:chloroform:isoamyl alcohol 25:24:1 (Sigma-Aldrich P2069) saturated with TE (10 mM Tris-HCl pH 8.0, 1 mM EDTA) and the DNA was then extracted. The aqueous phase was buffer exchanged to TE and unincorporated nucleotides were removed with Illustra MicroSpin G-50 columns (GE Healthcare).

For the experiments in Figures 7B and S7, frozen cell pellets corresponding to 2×10^8 cells were re-suspended in 400 μL lysis buffer (50 mM Tris-HCl pH 8.0, 0.1 M NaCl, 10 mM EDTA, 1% SDS) and the cell wall removed by incubation with 80 units / ml Lyticase (Sigma) and 1% β-mercaptoethanol at 37°C for 5 min. DNA was then extracted with phenol/chloroform/isoamylalcohol (25:24:1) and

the aqueous layer collected using phase lock tubes (SLS, 2302800). The DNA was then precipitated with 2 volumes of 100% ethanol and washed with 70% ethanol before being re-solubilized in 120 μL 10 mM Tris pH 8.0.

Decatenation of replicated plasmids with *E. coli* TopoIV

For post-reaction treatment with *E. coli* TopoIV (Figure 6), replication reactions were processed as above and then treated with TopoIV (Inspiralis, T4001) at 37°C for 30 min as per the manufacturer's instructions. The reactions were quenched and the samples prepared for gel analysis as above.

One-dimensional agarose gel electrophoresis and Southern blotting

Samples for denaturing agarose gels were supplemented with 20 mM EDTA, and 1/10 volume alkaline loading dye (0.5 M NaOH, 10% sucrose, xylene cyanol) and then left to denature at room temperature for 10 min. For native gels, samples were mixed with 1/6 volume native loading dye (30% glycerol, 0.25% bromophenol blue). For restriction digestion of the replicated products, 8-14 μL of sample was incubated in 1x CutSmart buffer with 0.25 μL restriction enzyme at 37°C for 30 min, except for SmaI that was incubated at 25°C for 30 min.

Samples were analyzed in 0.6 - 0.8% denaturing agarose gels at 21 V overnight in 30 mM NaOH, 2 mM EDTA, or 0.8% native agarose gels at 20 V overnight in 1X TAE. EtBr was included at 0.5 μg/ml for Figure 6D.

For the experiments in Figure 7B and Figure S7, the samples were electrophoresed at 0.4 V / cm for 7 days in 0.8% agarose (Megasieve) in 0.5x TBE. Following neutral Southern blotting onto Hybond-N+ membrane (GE Healthcare), the plasmids were detected by hybridization with a *LEU2* DNA probe that also detected the genomic *leu2* locus. Labeling and detection were performed with the 'random prime labeling module' incorporating fluorescein tagged dUTP (Roche, 11585622910). After hybridization and washing, fluorescein tagged dUTP was detected with alkaline phosphatase tagged anti fluorescein Fab fragments (Roche, 000000011426338910), and revealed with CDP-Star (GE Healthcare, GERPN3682). Images were acquired with the ImageQuant 4000 system (GE Healthcare).

Two-dimensional native-native agarose gel electrophoresis

The method was adapted from that described previously (Brewer and Fangman, 1987). In an analogous experiment to that in Figure 7B, involving control and *pif1-m2 rrm3Δ* cells containing the plasmid pRS425, DNA equivalent to 4×10^7 cells was prepared from the 40 minute time point. The DNA was then digested with 110 units of SnaB1 enzyme (New England Biolabs R0130M) in 1x 'CutSmart buffer' in a total volume of 50 μL at 37°C for 4 h.

20 μL of the digested volume was then loaded onto a 0.4% native agarose gel which was run 1 V / cm in 1x TBE for 15 h at room temperature. The lanes were then cut out and reset in a second dimension of 1% agarose containing 0.3 μg/ml EtBr and run in 1x TBE containing 0.3 μg/ml EtBr for 8 h at 5 V / cm in the cold room. The resultant gel was then prepared for Southern blotting and non-radioactive hybridization, as described above for one-dimensional gels.

Gel imaging and presentation

For *in vitro* replication experiments, native gels were dried directly onto chromatography paper (GE Healthcare, 3030-861). Denaturing gels were fixed by two incubations (20 min, 4°C) in cold 5% trichloroacetic acid and then dried onto chromatography paper. The dried gels were typically exposed to both Amersham Hyperfilm ECL (GE Healthcare) and BAS-MS Imaging Plates (Fujifilm), which were then developed on a Typhoon phosphorimager (GE Healthcare).

QUANTIFICATION AND STATISTICAL ANALYSIS

For quantification of replication products, gel images generated on a Typhoon phosphorimager were converted to 16-Bit Tiff files using the Linearize GelData plugin in ImageJ (National Institute of Health). Boxes were drawn around each lane and the peaks corresponding to LRIs and full-length products were selected manually from the resultant lane profiles. The 'percentage full-length products' was then calculated, relative to all the replication products in a given lane.

The corresponding experiments were performed the following number of times: Figure 1C (3x), Figure 1D (3x), Figure 1F (2x), Figure 2A (3x), Figure 2C (2x), Figure 2D (2x), Figure 3B (2x), Figure 3C (3x), Figure 4A (2x), Figure 4C (2x), Figure 4D (3x), Figure 4E (3x), Figure 4F (2x), Figure 5B (2x), Figure 5C (3x), Figure 5D (3x), Figure 5E (1x), Figure 5F (1x), Figure 6A (3x), Figures 6C and 6D (3x), Figure 7B (3x), Figure 7B (2x), Figure S1D (2x), Figure S1E (2x), Figure S1G (2x), Figure S2A (2x), Figure S2B (2x), Figure S3A (1x), Figures S3B and S3C (3x), Figure S3D (1x), Figure S3E (1x), Figure S3F (2x), Figure S4A (2x), Figure S4B (2x), Figure S5A (1x), Figure S5B (1x), Figure S5C (2x), Figure S6A (3x), Figure S7A (3x). The analyses in Figures 1E, S1F, S3G and S3H formed part of a number of other experiments, and were thus repeated many times.

Molecular Cell, Volume *74*

Supplemental Information

Pif1-Family Helicases Support Fork Convergence during DNA Replication Termination in Eukaryotes

Tom D. Deegan, Jonathan Baxter, María Ángeles Ortiz Bazán, Joseph T.P. Yeeles, and Karim P.M. Labib

Figure S1, related to Figure 1

Convergence of reconstituted replisomes is defective over a wide range of experimental conditions. (**A**) Plasmids used in this study, with restriction sites that were used for linearisation. (**B**) Reaction scheme for standard *in vitro* replication reactions. (**C**) *S. cerevisiae* DNA replication proteins were purified as described in STAR Methods, and then visualised by SDS-PAGE and coomassie staining. (**D**) Fork convergence is defective during replication of different plasmid templates (see STAR methods and (A) for details). Reaction products were linearised before native agarose gel analysis with the indicated enzymes. (**E**) Replication of the 3.2 kb plasmid template in the indicated concentrations of potassium glutamate. The reaction products were purified, linearised with SbfI and then analysed in a native agarose gel. (**F**) The 3.2 kb plasmid was replicated for 20' in the presence ^{32}P-dCTP, before addition of a chase of 600 μM unlabelled dNTPs. Samples were taken at the indicated times, before analysis by digestion and native agarose gel electrophoresis, indicating that LRIs persist stably and thus represent an endpoint of the replication reaction under these conditions. (**G**) The 3.2 kb template was replicated for the indicated times in the presence of a 'minimal' replisome (Yeeles et al., 2015). The reactions lacked Ctf4, Csm3-Tof1, Mrc1, Top2, Pol δ, RFC, PCNA, ligase and Fen1. As before, the purified samples were analysed by SmaI digestion and native agarose gel electrophoresis.

Figure S2, related to Figure 2

Replication of plasmids with a single active origin of DNA replication. (**A**) Micrococcal nuclease digestion of the chromatinized 3.2 kb plasmid template

used in Figure 2A. The products were analysed in a 1.5% native agarose gel with ethidium bromide staining. (**B**) Replication of the circular (1) and linearised (2-3) versions of the 5.5 kb plasmid template shown in Fig. 2B. The reactions were performed in the absence of Polδ, ligase and Fen1, and were analysed by denaturing agarose gel electrophoresis. The figure indicates the positions of the ~2.75 kb leading strand products for the circular vector and the ~5.5 kb leading strand products for the linearised plasmids.

Figure S3, related to Figure 4

The ability of accessory DNA helicases to support fork convergence in the reconstituted DNA replication system. (**A**) Control experiment, in which the 3.2 kb plasmid was incubated with Sgs1, Top3-Rmi1 and RPA, showing that purified Sgs1 and Top3-Rmi1 are active for relaxation and catenation of dsDNA. The positions of the various plasmid isoforms are indicated. Sgs1 and Top3-Rmi1 were omitted as indicated. (**B**) Analogous reactions to those in Figure 4C were performed in the presence of the indicated concentrations of Sgs1-Top3-Rmi1 (Sgs1-T-R), before analysis by native agarose gel electrophoresis. (**C**) Samples from A were analysed by denaturing agarose gel electrophoresis, illustrating that higher concentrations of Sgs1-Top3-Rmi1 inhibited DNA synthesis in the reconstituted replication system. (**D**-**H**) The 3.2 kb plasmid template was replicated in the presence of varying concentrations of the indicated proteins. The reaction products were purified, digested, and analysed in native agarose gels.

Figure S4, related to Figure 4

Pif1 supports fork converge on chromatinised and linearised plasmid templates. (**A**) A chromatinised versions of the 3.2 kb plasmid was prepared as for Figure 2A, and replicated in the presence of FACT, with or without Pif1 as indicated. The products were digested with SpeI and analysed in a native agarose gel. (**B**) Pulse-chase replication reaction conducted as depicted in Figure 3A with 9.7 kb plasmid template. Pif1 was added as indicated, concomitantly with SmaI, to test whether Pif1 could promote fork convergence on a linearised DNA template. Those samples that were not linearised during the experiment (1-2) were subsequently digested with SmaI before native gel analysis.

Figure S5, related to Figure 5

Pif1-Rrm3 support fork convergence but CMG is still essential for replication. (**A**) The 9.7 kb plasmid template pZN3 was replicated in the presence of ^{32}P-dCTP for 2' 50", before addition of a chase of cold dNTPs, together with Pif1+Rrm3 as indicated. Pol δ, ligase and Fen1 were omitted from these reactions, so that un-ligated leading strands could be monitored. Samples were removed and quenched at the indicated times, before analysis in a denaturing agarose gel. (**B**) Replication reactions containing Pif1 but lacking the indicated factors that are required for CMG assembly and activation. Products were analysed by digestion and native agarose gel electrophoresis. (**C**) Similar reactions containing Rrm3 but lacking the indicated factors.

Figure S6, related to Figure 6

Type II topoisomerase activity makes a modest contribution to fork convergence in the reconstituted replication system. (**A**) Replication of the 3.2 kb plasmid template in the presence of the indicated topoisomerases from *S. cerevisiae* (*S.c.*) or *E. coli* (*E.c.*). Products were analysed by SbfI digestion and native agarose gel electrophoresis. (**B**) Similar experiments to (A) were performed three times. The percentage full-length products was quantified in each case, and the figure presents the mean values with standard deviations (**C**) Analogous experiments to the one in Figure 6A were performed three times, and then quantified as above.

Figure S7, related to Figure 7

Pif1 and Rrm3 have a partially redundant role in promoting fork convergence during plasmid replication *in vivo*. (**A**) Control cells (W303-1a), *rrm3Δ* (yBH131), *pif1Δ* (yMO294) and *pif1-m2 rrm3Δ* (yMO291) were grown and analysed as in Figure 7A-B. The positions of the various plasmid isoforms are indicated, and the red arrow denotes late replication intermediates (LRIs), which accumulated to a greater extent in *pif1-m2 rrm3Δ* cells compared to the *rrm3Δ* single mutant. (**B**) The plasmid pRS425, illustrating the SnaBI restriction site that was used for linearization in Figure 7C, and the Late Replication Intermediate (LRI) that results from transient stalling of forks during DNA replication termination *in vivo*.

Figure S1, related to Figure 1

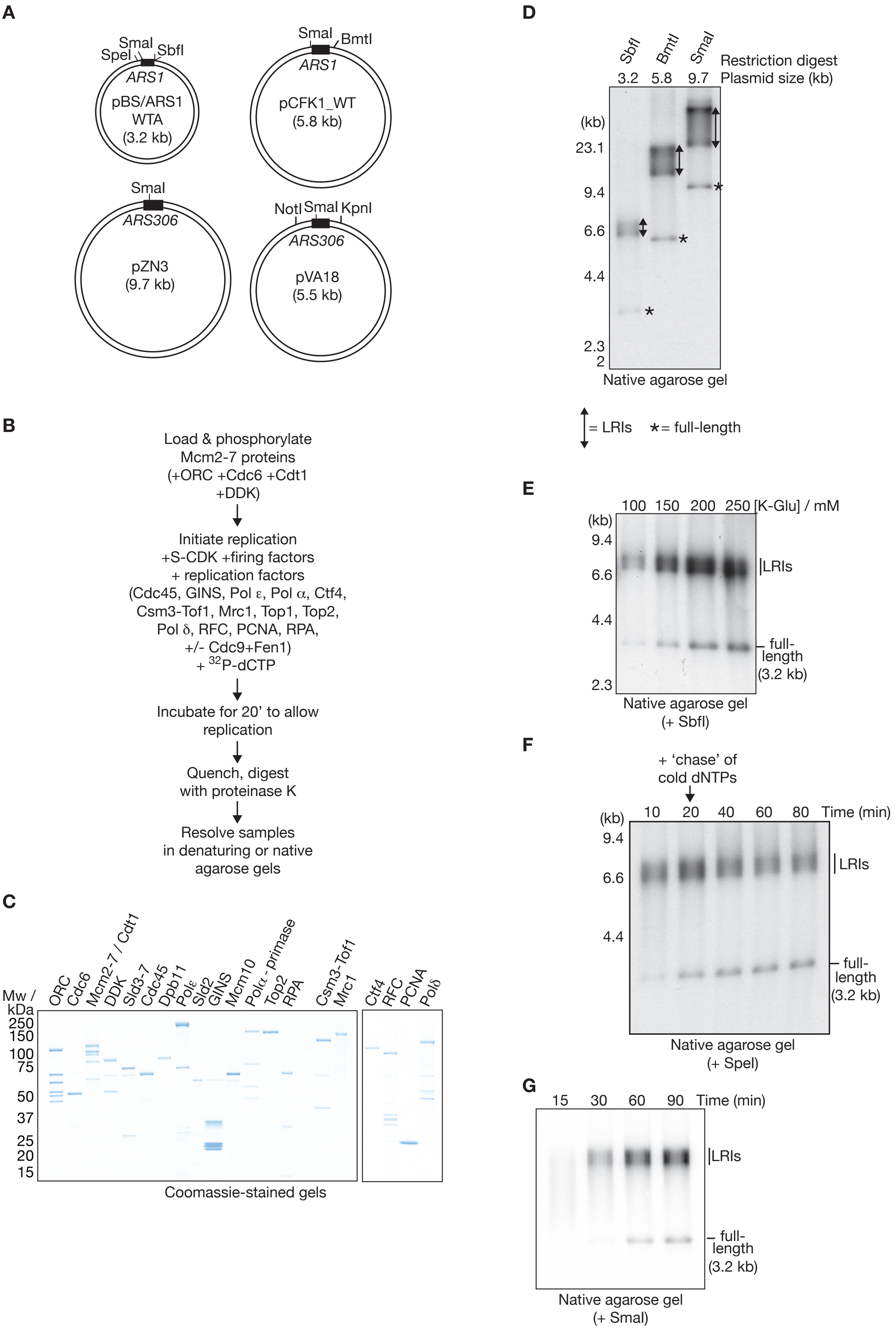

Figure S2, related to Figure 2

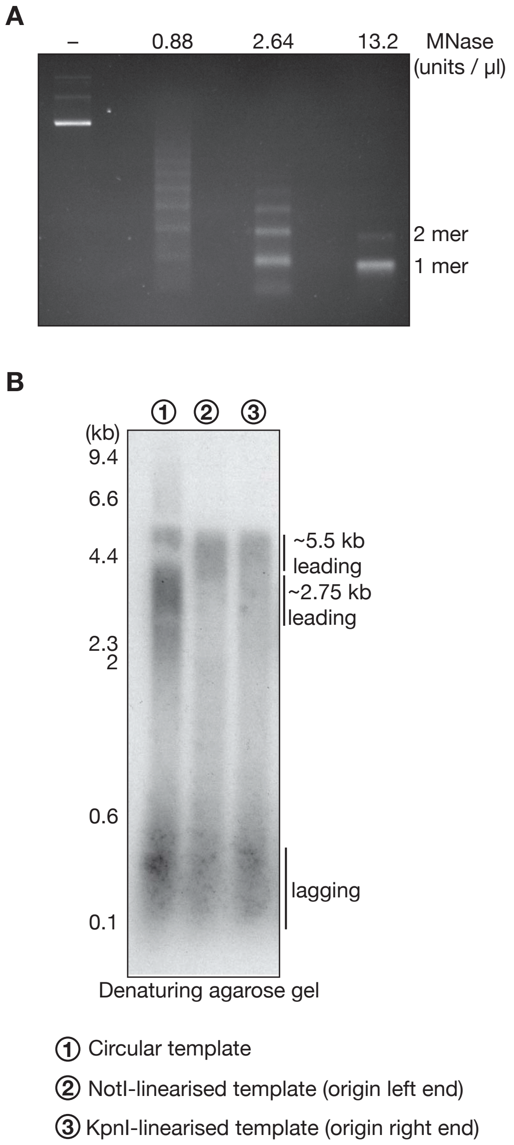

Figure S3, related to Figure 4

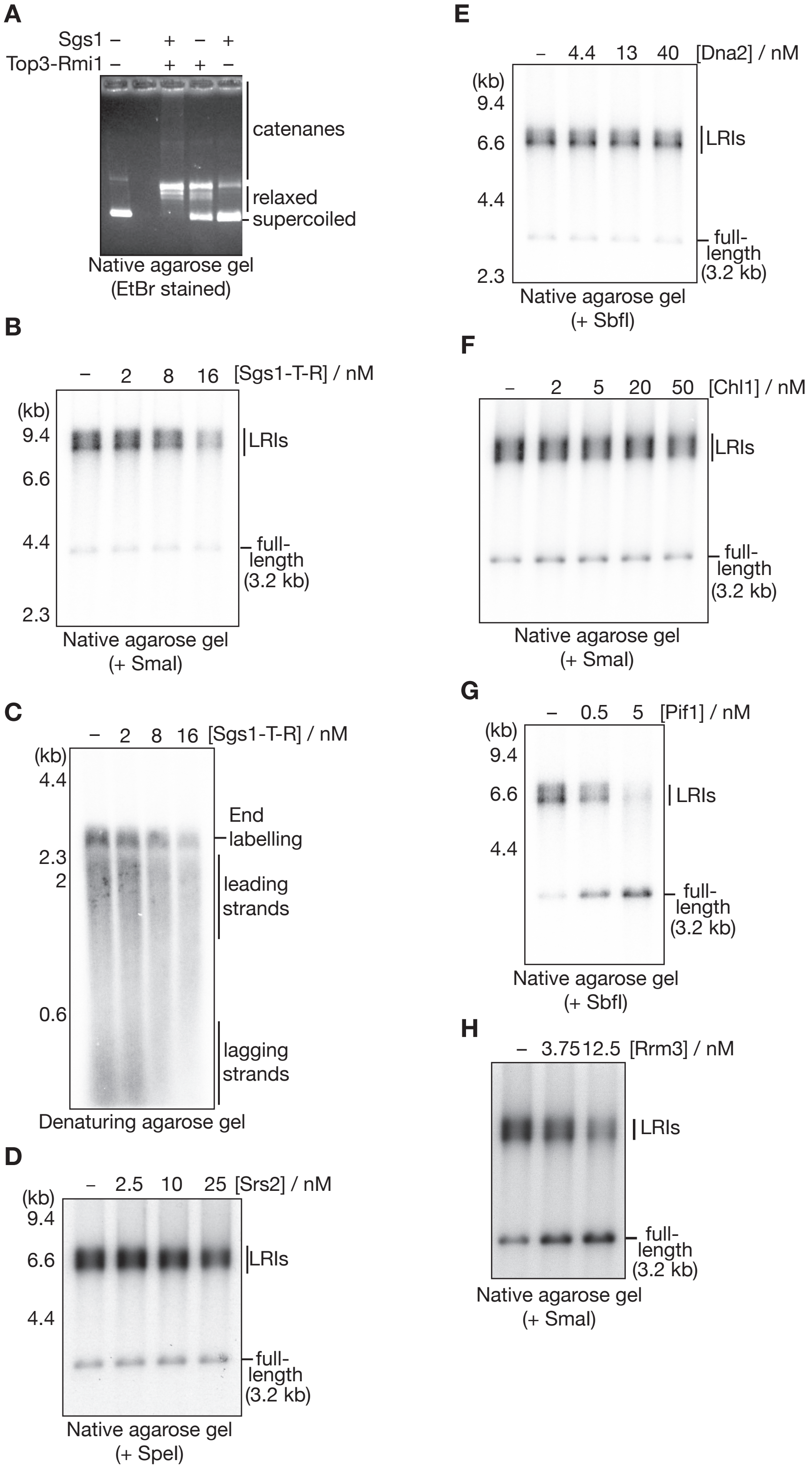

Figure S4, related to Figure 4

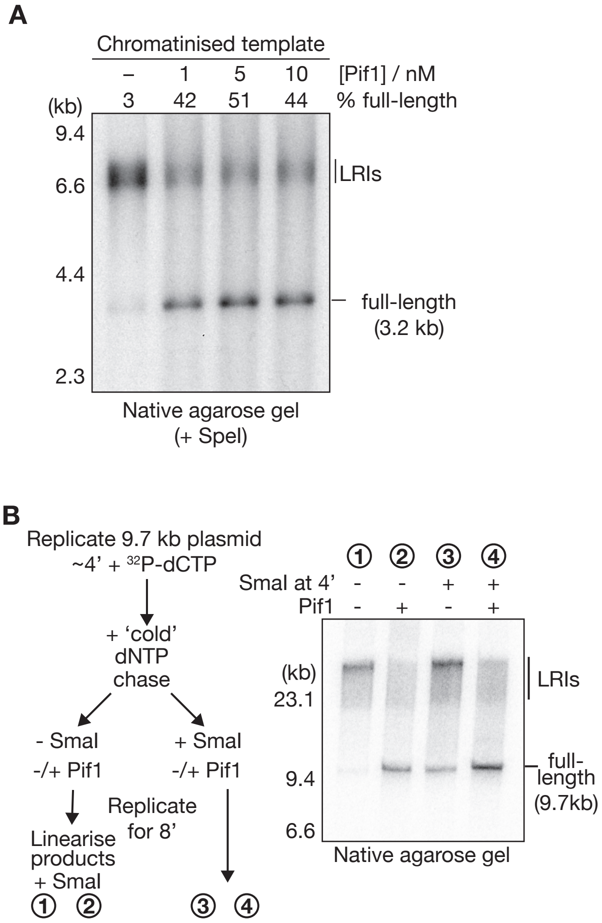

Figure S5, related to Figure 5

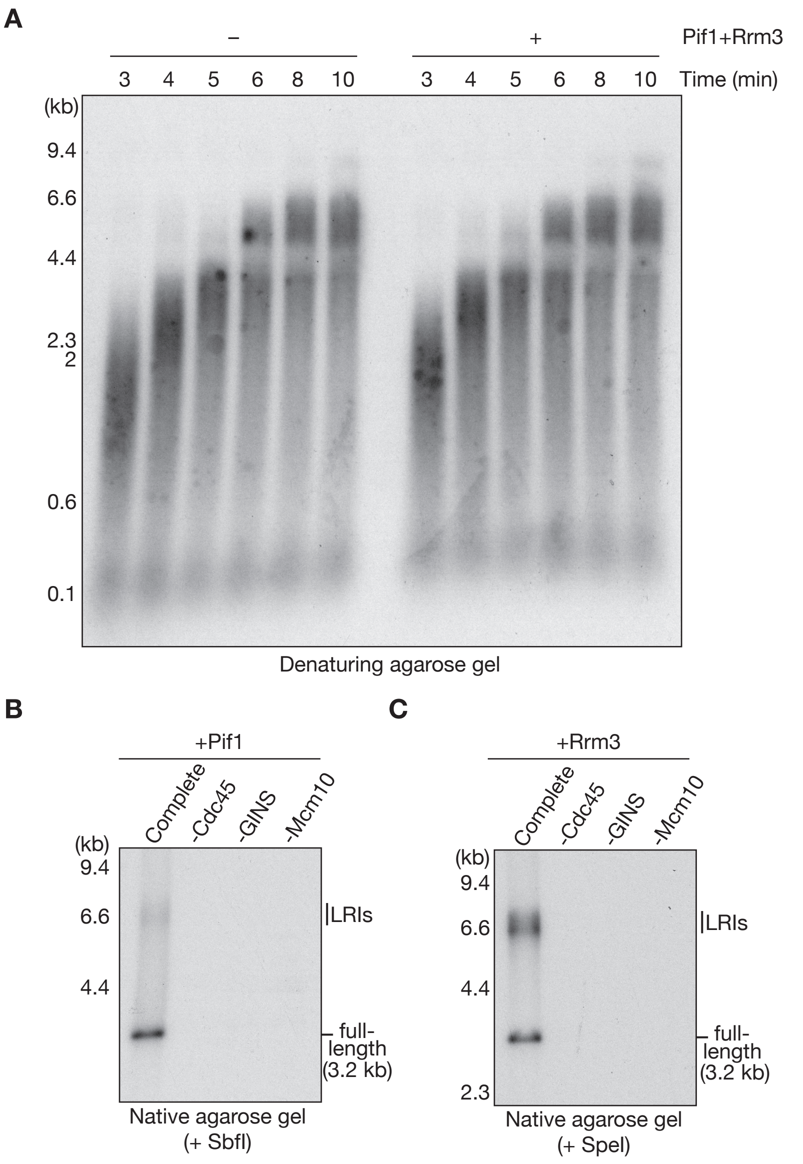

Figure S6, related to Figure 6

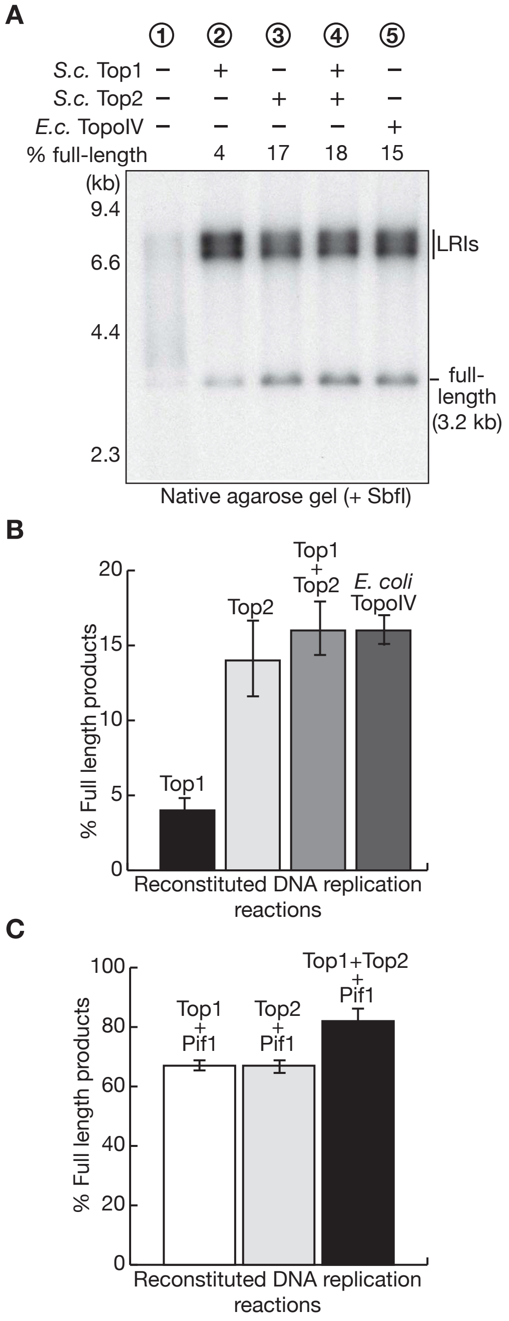

Figure S7, related to Figure 7

A

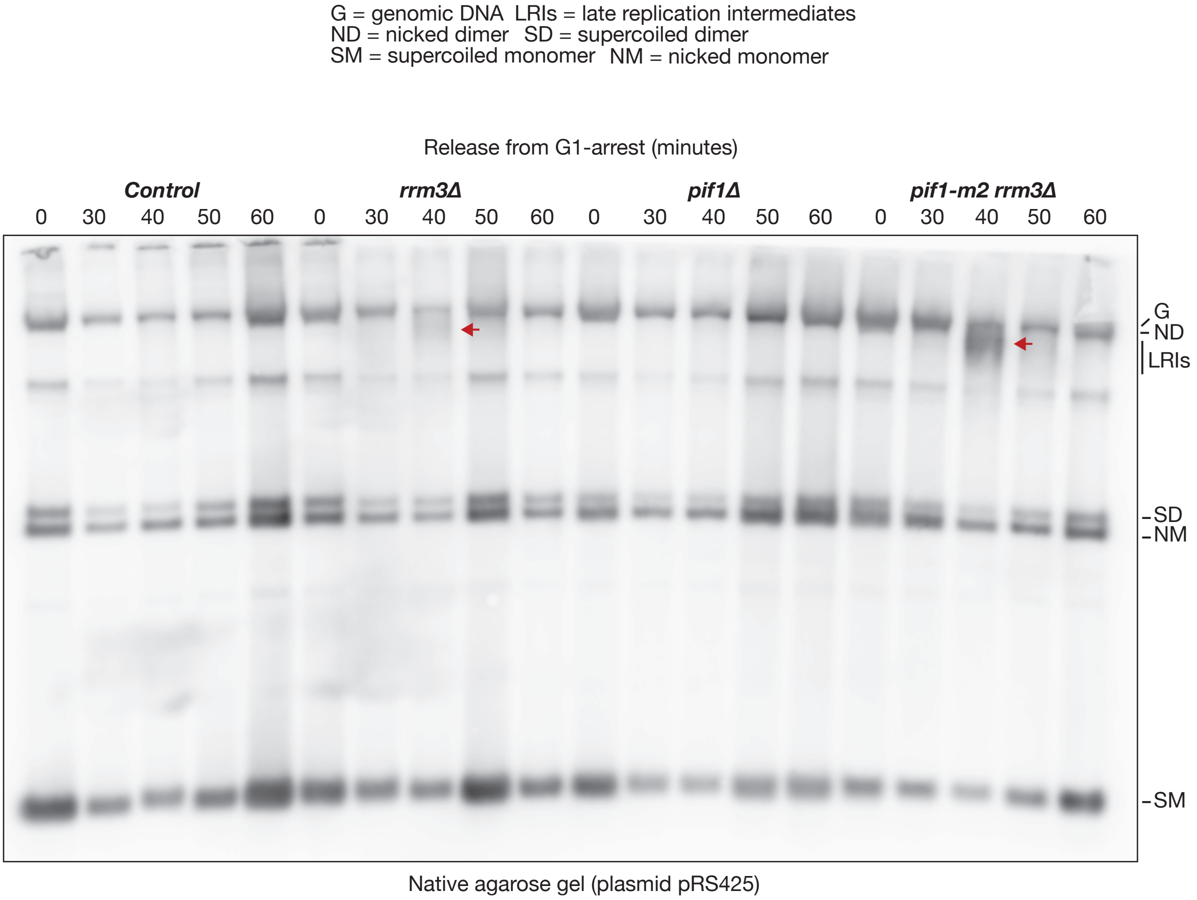

B

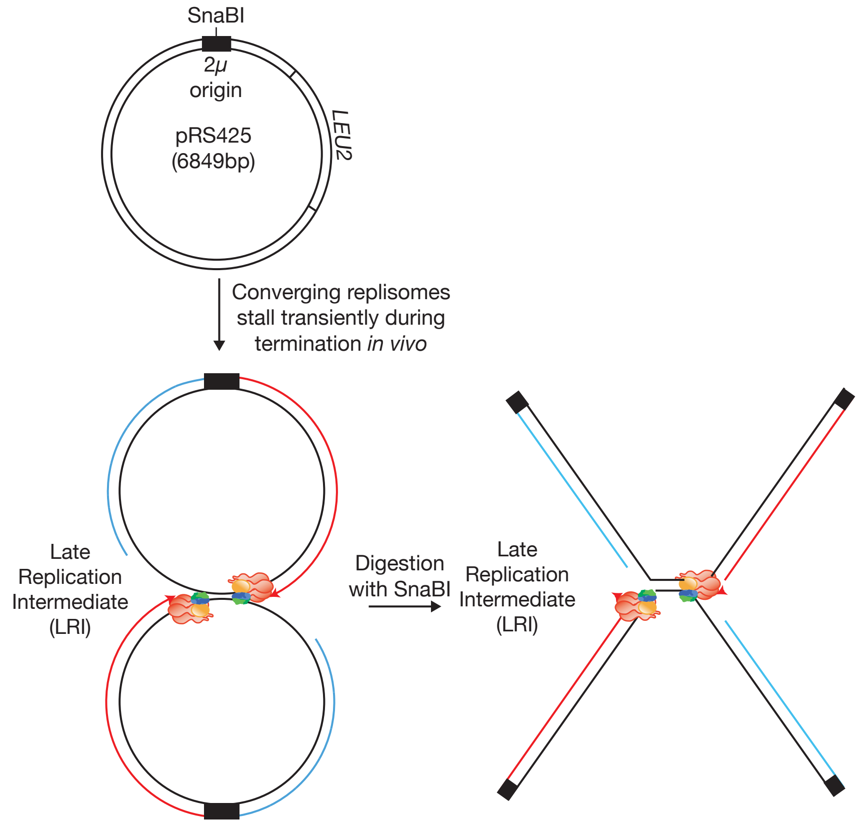

Plasmid	Original vector	Insert	Plasmid construction
pTDK4	pRS305	*Tof1-GAL1,10-CBP-TEV-Csm3*	Synthetic SacI-XmaI construct
pTDK8	pRS305	*Gal1,10-CBP-TEV-Top1*	Synthetic SacI-XmaI construct
pTDK13	pTDK8	*CBP-TEV-Sgs1*	Synthetic SpeI-XmaI construct
pTDK15	pTDK8	*3Flag-TEV-Rrm3*	Synthetic SpeI-XmaI construct
pTDK18	pTDK8	*Fen1-2Flag*	Synthetic SpeI-XmaI construct
pTDK19	pTDK8	*Cdc9-2Flag*	Synthetic SpeI-XmaI construct
pTDK34	pTDK15	*3Flag-TEV-Rrm3 K260A*	Site directed mutagenesis of pTDK15 using primers 7813 and 7814
pTDK10	pET28c	*Pif1 nuclear isoform (Pif1Δ2-40)*	PCR amplification of Pif1Δ2-40 from W303 genomic DNA using primers 6912 and 6913 cloned 5' NdeI, 3' SalI
pTDK24	pTDK10	*Pif1 nuclear isoform (Pif1Δ2-40) K264A*	Site directed mutagenesis of pTDK10 using primers 7103 and 7104
pTDK31	pET28c	*Pif1 from Bacteroides sp 2 1 16 (BacPif1)*	Synthetic NdeI-NotI construct
pFV36	pKL120	*PrA-CBP-TEV-Dna2*	Synthetic SpeI-XmaI construct

Table S1, Related to STAR Methods. Protein expression plasmids generated in this study. Codon usage of genes in pTDK4, pTDK8, pTDK13, pTDK15, pTDK18, pTDK19, pTDK34 and pFV36 was optimised for protein expression in *S. cerevisiae* as described previously (Yeeles et al., 2015).

Oligonucleotide name	Usage	Sequence
6912	Pif1 forward primer for construction of pTDK10	ACGCACATATGAGTAGTCGTGGT TTCAGG
6913	Pif1 reverse primer for construction of pTDK10	TAGTCGTCGACTTATTCTAAGAT GTGGTCTTCG
7080	Oligonucleotide used for annealing to M13 ssDNA for generation of helicase assay substrate with 40bp duplex	GATCGACTACGTCAGCTCTAGAG GATCCCCGGGTACCGAGCTCGA ATTCGTAATC
7103	Forward primer for mutagenesis of Pif1 to generate pTDK24	GGGAGTGCCGGTACCGGTGCAT CCATTCTTTTACGTG
7104	Reverse primer for mutagenesis of Pif1 to generate pTDK24	CACGTAAAAGAATGGATGCACC GGTACCGGCACTCCC
7272	Forward primer for generation of molecular weight markers	ATGGTTACTAAGCCATCCC
7273	Reverse primer for generation of 2.9 kb molecular weight marker	GCTTGTCAATACCCATACC
7274	Reverse primer for generation of 3 kb molecular weight marker	CAGTAGGAGTAGTTACCG
7275	Reverse primer for generation of 3.1 kb molecular weight marker	CTTGTTCAAGTGCTTTTCC
7813	Forward primer for mutagenesis of Rrm3 to generate pTDK34	GGTTCCGCTGGTACTGGTGCTT CTGTTATTTTGCAAACC
7814	Reverse primer for mutagenesis of Rrm3 to generate pTDK34	GGTTTGCAAAATAACAGAAGCAC CAGTACCAGCGGAACC
8371	Oligonucleotide used for annealing to M13 ssDNA for generation of helicase assay substrate with 25bp duplex	ACTACGTCAGTACCGAGCTCGAA TTCGTAATCATG

Table S2, Related to STAR Methods. Oligonucleotides used in this study. All oligonucleotides were generated for this study.

Protein	Tag	Purification steps
ORC	CBP-TEV (Orc1)	1. Calmodulin affinity purification<br>2. HiTrap Q chromatography
Cdc6	GST	1. GST affinity purification<br>2. Elution by cleavage with 3C protease<br>3. Hydroxyapatite chromatography
Cdt1-Mcm2-7	CBP-TEV (Mcm3)	1. Calmodulin affinity purification<br>2. Gel filtration (Superdex 200)
DDK	CBP-TEV (Dbf4)	1. Calmodulin affinity purification<br>2. Dephosphorylation (Lambda protein phosphatase)<br>3. Gel filtration (Superdex 200)
S-CDK	CBP-TEV (Clb5)	1. Calmodulin affinity purification<br>2. Elution by cleavage with TEV protease<br>3. Gel filtration (Superdex 200)
Sld3-7	TEV-CBP-PrA (Sld3)	1. IgG affinity purification<br>2. Elution by cleavage with TEV protease<br>3. Gel filtration (Superdex 200)
Cdc45	Internal 2FLAG	1. Anti-FLAG affinity purification<br>2. HiTrap Q chromatography
Dpb11	3FLAG	1. Anti-Flag affinity purification<br>2. MonoS chromatography
Pol ε	TEV-CBP (Dpb4)	1. Calmodulin affinity purification<br>2. HiTrap heparin chromatography<br>3. Gel filtration (Superdex 200)
Sld2	3FLAG	1. Ammonium sulphate precipitation<br>2. Anti-Flag affinity purification<br>3. HiTrap SP chromatography
GINS	6HIS (Psf3)	1. Ni-NTA affinity purification<br>2. HiTrap Q chromatography<br>3. Gel filtration (Superdex 200)
Mcm10	6HIS	1. Ni-NTA affinity purification

		2. MonoS chromatography (two rounds)<br>3. Gel filtration (Superdex 200)
Ctf4	CBP-TEV	1. Calmodulin affinity purification<br>2. Gel filtration (Superdex 200)
Top2	TEV-CBP	1. Calmodulin affinity purification<br>2. Gel filtration (Superdex 200)
PCNA	Untagged	1. Polymin P precipitation of nucleic acids<br>2. Ammonium sulphate precipitation of proteins<br>3. HiTrap SP and HiTrap heparin chromatography (in tandem)<br>4. HiTrap DEAE chromatography<br>5. HiTrap Q chromatography<br>6. Gel filtration (Superdex 200)
RPA	CBP-TEV (Rfa1)	1. Calmodulin affinity purification<br>2. HiTrap heparin chromatography<br>3. HiTrap Q chromatography
ISW1	3FLAG (Ioc3)	1. Anti-Flag affinity purification<br>2. MonoQ chromatography
Nap1	GST	1. GST affinity purification<br>2. Elution by cleavage with 3C protease<br>3. MonoQ chromatography
Nhp6	Untagged	1. Trichloroacetic acid precipitation<br>2. HiTrap SP chromatography
FACT	HIS	1. TALON affinity purification<br>2. MonoQ chromatography
Histones	Untagged	1. HiTrap heparin chromatography<br>2. Gel filtration (Superdex 200)

Table S3, Related to STAR Methods. Protein purification strategies used in this study.

Protein	Source
ORC	(Frigola et al., 2013)
Cdc6	(Frigola et al., 2013)
Cdt1-Mcm2-7	(Coster et al., 2014)
DDK	(On et al., 2014)
Sld3/7	(Yeeles et al., 2015)
Cdc45	(Yeeles et al., 2015)
Dpb11	(Yeeles et al., 2015)
Sld2	(Yeeles et al., 2015)
Pol ε	(Yeeles et al., 2015)
GINS	(Yeeles et al., 2015)
S-CDK	(Yeeles et al., 2015)
Mcm10	(Yeeles et al., 2015)
Pol α	(Yeeles et al., 2015)
RPA	(Yeeles et al., 2015)
Ctf4	(Yeeles et al., 2015)
Mrc1	(Yeeles et al., 2017)
Csm3-Tof1	This study
RFC	(Yeeles et al., 2017)
PCNA	(Yeeles et al., 2017)
Pol δ	(Yeeles et al., 2017)
Top1	This study
Top2	(Yeeles et al., 2015)
Fen1	This study
Cdc9	This study
ISWI	(Kurat et al., 2017)
Nap1	(Kurat et al., 2017)
Nhp6	(Kurat et al., 2017)
FACT	(Kurat et al., 2017)
Histones	(Kurat et al., 2017)
Sgs1	Kowalczykowski lab
Top3-Rmi1	Kowalczykowski lab
Pif1	This study
BacPif1	This study
Rrm3	This study
Chl1	This study
Dna2	This study
Srs2	This study
TopoIV	Inspiralis (T4001)

Table S4, Related to STAR Methods. Recombinant proteins used in this study.

Strain	Source
*W303-1a: MAT***a** *ade2-1 ura3-1 his3-11,15 trp1-1 leu2-3,112 can1-100*	Labib Laboratory
*yJF1: MAT***a** *ade2-1 ura3-1 his3-11,15 trp1-1 leu2-3,112 can1-100 bar1Δ::hphNT pep4Δ::kanMX*	(Frigola et al., 2013)
*ySDORC (ORC purification): MAT***a** *ade2-1 ura3-1 his3-11,15 trp1-1 leu2-3,112 can1-100 bar1::hphNT pep4::kanMx his3::pRS303-ORC3+ORC4 ura3::pRS306-CBP-TEV-ORC1+ORC2 trp1::pRS304-ORC5+ORC6*	(Frigola et al., 2013)
*yAM33 (Cdt1-Mcm2-7 purification): MAT***a** *ade2-1 ura3-1 his3-11,15 trp1-1 leu2-3,112 can1-100 bar1::hphNT pep4::kanMx his3::pRS303-CDT1+GAL4 ura3::pRS306-MCM2+CBP-TEV-MCM3 trp1::pRS304-MCM4+MCM5 leu2::pRS305-MCM6+MCM7*	(Coster et al., 2014)
*ySDK8 (DDK purification): MAT***a** *ade2-1 ura3-1 his3-11,15 trp1-1 leu2-3,112 can1-100 bar1::hphNT pep4::kanMX trp1::pRS304-CDC7+CBP-TEV-DBF4*	(On et al., 2014)
*yTD6 (Sld3-7 purification): MAT***a** *ade2-1 ura3-1 his3-11,15 trp1-1 leu2-3,112 can1-100 bar1::hphNT pep4::kanMX leu2::pRS305-SLD7 his3::pRS303-SLD3-TCP+GAL4*	(Yeeles et al., 2015)
*yTD8 (Sld2 purification): MAT***a** *ade2-1 ura3-1 his3-11,15 trp1-1 leu2-3,112 can1-100 bar1::hphNT pep4::kanMX his3::pRS303-SLD2-3FLAG(nat-NT2)+GAL4*	(Yeeles et al., 2015)
*yJY13 (Cdc45 purification): MAT***a** *ade2-1 ura3-1 his3-11,15 trp1-1 leu2-3,112 can1-100 bar1::hphNT pep4::kanMX his3::pRS303-CDC45-iFLAG2+GAL4*	(Yeeles et al., 2015)
*yJY26 (Dpb11 purification): MAT***a** *ade2-1 ura3-1 his3-11,15 trp1-1 leu2-3,112 can1-100 bar1::hphNT pep4::kanMX his3::pRS303-DPB11-3FLAG(nat-NT2)+GAL4*	(Yeeles et al., 2015)
yAJ2 (Pol ε *purification): MAT***a** *ade2-1 ura3-1 his3-11,15 trp1-1 leu2-3,112 can1-100 bar1::hphNT pep4::kanMX trp1::pRS304-POL2+DPB4-TEV-CBP ura3::pRS306DPB2+DPB3*	(Yeeles et al., 2015)
*yAE37(S-CDK purification): MAT***a** *ade2-1 ura3-1 his3-11,15 trp1-1 leu2-3,112 can1-100 bar1::hphNT pep4::kanMX ura3::pRS306-CKS1+CDC28 his3::pRS303-CBP-TEV-CLB5+GAL4*	(Yeeles et al., 2015)
*yAE40 (Ctf4 purification): MAT***a** *ade2-1 ura3-1 his3-11,15 trp1-1 leu2-3,112 can1-100 bar1::hphNT pep4::kanMX his3::pRS303-CBP-TEV-CTF4+GAL4*	(Yeeles et al., 2015)
yJY23 (Pol α */ primase purification): MAT***a** *ade2-1 ura3-1 his3-11,15 trp1-1 leu2-3,112 can1-100 bar1::hphNT pep4::kanMX trp1::pRS304-POL1+POL12 ura3:: pRS306-CBP-TEV-PRI1+PRI2*	(Yeeles et al., 2015)

*yAE34 (Pol δ purification): MAT***a** *ade2-1 ura3-1 his3-11,15 trp1-1 leu2-3,112 can1-100 bar1::hphNT pep4::kanMX ura3::pRS306-POL31+POL3 his3::pRS303-POL32-CBP+GAL4*	(Yeeles et al., 2017)
*yAE41 (RFC purification): MAT***a** *ade2-1 ura3-1 his3-11,15 trp1-1 leu2-3,112 can1-100 bar1::hphNT pep4::kanMX ura3::pRS306-RFC2+CBP-RFC3 trp1::pRS304-RFC4+RFC5 his3::pRS303-RFC1+GAL4*	(Yeeles et al., 2017)
*yAE71 (Mrc1 purification): MAT***a** *ade2-1 ura3-1 his3-11,15 trp1-1 leu2-3,112 can1-100 bar1::hphNT pep4::kanMX his3::pRS303-MRC1-5FLAG*	Diffley laboratory
*yAE31 (RPA purification): MAT***a** *ade2-1 ura3-1 his3-11,15 trp1-1 leu2-3,112 can1-100 bar1::hphNT pep4::kanMX his3::pRS303-CBP-TEV-RFA1+GAL4 ura3::pRS306-RFA2+RFA3*	(Yeeles et al., 2017)
*yAE46 (Top2 purification): MAT***a** *ade2-1 ura3-1 his3-11,15 trp1-1 leu2-3,112 can1-100 bar1::hphNT pep4::kanMX trp1::pRS304-TOP2-TEV-CBP+GAL4*	(Yeeles et al., 2015)
*yCFK1 (Isw1a purification): MAT***a** *ade2-1 ura3-1 his3-11,15 trp1-1 leu2-3,112 can1-100 bar1Δ::hphNT pep4Δ::kanMX IOC3-3FLAG(nat-NT2)*	(Kurat et al., 2017)
*yTDK4 (Csm3-Tof1 purification): MAT***a** *ade2-1 ura3-1 his3-11,15 trp1-1 leu2-3,112 can1-100 bar1Δ::hphNT pep4Δ::kanMX leu2::pRS305-TOF1+CBP-TEV-CSM3*	This study
*yTDK9 (Rrm3 purification): MAT***a** *ade2-1 ura3-1 his3-11,15 trp1-1 leu2-3,112 can1-100 bar1Δ::hphNT pep4Δ::kanMX leu2::pRS305-3FLAG-TEV-RRM3*	This study
*yTDK17 (Rrm3-K260A purification): MAT***a** *ade2-1 ura3-1 his3-11,15 trp1-1 leu2-3,112 can1-100 bar1Δ::hphNT pep4Δ::kanMX leu2::pRS305-3FLAG-TEV-rrm3-K260A*	This study
*yTDK6 (Top1 purification): MAT***a** *ade2-1 ura3-1 his3-11,15 trp1-1 leu2-3,112 can1-100 bar1Δ::hphNT pep4Δ::kanMX leu2::pRS305-CBP-TEV-TOP1*	This study
*yFV43 (Dna2 purification): MAT***a** *ade2-1 ura3-1 his3-11,15 trp1-1 leu2-3,112 can1-100 pep4Δ::ADE2 ura3::pRS306-ProteinA-CBP-TEV-DNA2*	This study
*yTDK18 (Fen1 purification): MAT***a** *ade2-1 ura3-1 his3-11,15 trp1-1 leu2-3,112 can1-100 bar1Δ::hphNT pep4Δ::kanMX leu2::pRS305-FEN1-2FLAG*	This study
*yTDK19 (Cdc9 purification): MAT***a** *ade2-1 ura3-1 his3-11,15 trp1-1 leu2-3,112 can1-100 bar1Δ::hphNT pep4Δ::kanMX leu2::pRS305-CDC9-2FLAG*	This study
*yBH131: MAT***a** *ade2-1 ura3-1 his3-11,15 trp1-1 leu2-3,112 can1-100 rrm3Δ::hphNT*	(Hodgson et al., 2007)
*yMO294: MAT***a** *ade2-1 ura3-1 his3-11,15 trp1-1 leu2-3,112 can1-100 pif1Δ::URA3CP*	This study
*yMO291: MAT***a** *ade2-1 ura3-1 his3-11,15 trp1-1 leu2-3,112 can1-100 pif1-m2 rrm3Δ::hphNT*	This study

Table S5, Related to STAR Methods. *S. cerevisiae* strains used in this study.
